# Development of a Brain‐Penetrant Nurr1 Agonist Tool

**DOI:** 10.1002/cmdc.70296

**Published:** 2026-06-14

**Authors:** Jan Vietor, Tanja Stiller, Christian Gege, Wael Saeb, Úrsula López‐García, Hella Kohlhof, Daniel Vitt, Daniel Merk

**Affiliations:** ^1^ Department of Pharmacy Ludwig‐Maximilians‐Universität München Munich Germany; ^2^ Immunic AG Gräfelfing Germany; ^3^ RebisLab R&D GmbH Planegg‐Martinsried Germany

**Keywords:** blood‐brain‐barrier, NR4A, nuclear receptor‐related 1, transcription factor

## Abstract

The ligand‐sensing transcription factor nuclear receptor‐related 1 (Nurr1), is thought to mediate neuroprotective activity and is implicated in various neurodegenerative diseases. Nurr1 ligands have been developed as tools to capture the receptor's potential in vitro and in vivo, but a dedicated brain‐targeting agonist has been lacking. Here, we employed the scaffold of the dihydroorotate dehydrogenase (DHODH) inhibitor and strong Nurr1 activator vidofludimus to develop a descendant with a high brain‐to‐blood ratio. Scaffold‐hopping from cyclopentene to thiophene, enhanced fluorination, and replacement of the carboxylic acid by an amide provided a highly potent, selective, and brain‐penetrant Nurr1 agonist to study the effects of Nurr1 activation in the central nervous system (CNS).

## Graphical abstract

Nuclear receptor‐related 1 (Nurr1) is a ligand‐activated transcription factor with neuroprotective properties, thought to hold therapeutic potential in neurodegeneration. Here, we developed a potent, brain‐penetrant Nurr1 agonist to study the effects of pharmacological Nurr1 activation in the CNS in vivo.

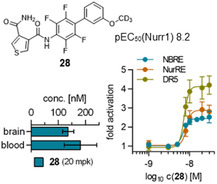

**Abbreviations**
AD: Alzheimer's disease
AQ: Amodiaquine
BBB: Blood–brain barrier
CNS: Central nervous system
DHI: 5,6‐dihydroxyindole
DHODH: Dihydroorotate dehydrogenase
ITC: Isothermal titration calorimetry
LBD: Ligand binding domain
MS: Multiple sclerosis
NOR1: Neuron‐derived orphan receptor
Nur77: Nerve growth factor‐induced protein I‐B
Nurr1: Nuclear receptor‐related 1
PD: Parkinson's diseases
PGA1: Prostaglandin A1
PK: Pharmacokinetics

## 1 Introduction

Nuclear receptor‐related 1 (Nurr1, NR4A2) [1] is an orphan nuclear receptor whose pharmacological activation is considered a promising therapeutic approach in neurodegenerative diseases [2, 3, 4, 5, 6, 7]. It acts as a neuroprotective and anti‐inflammatory transcription factor, and is mainly found in the central nervous system (CNS) with high expression levels in (dopaminergic) neurons and astrocytes [8, 9, 10, 11]. Patients with neurodegenerative pathology (Alzheimer's disease (AD) and Parkinson's disease (PD)) displayed reduced Nurr1 expression levels [12], and recently developed Nurr1 agonists enhanced neuroprotective gene expression [13, 14, 15, 16], supporting a protective role of the receptor. Prostaglandin A1 (PGA1) [17] and the dopamine metabolite 5,6‐dihydroxyindole (DHI) [18] have been identified as endogenously occurring Nurr1 ligands, aligning with the receptor's role in regulating inflammatory responses [11] and dopamine metabolism [19, 20].

**SCHEME 1:**
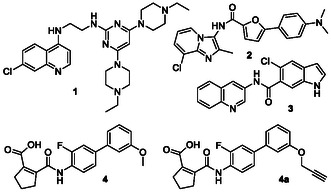
Nurr1 Modulators.

The transcription factor's promising potential as an antineurodegenerative target has fueled growing interest in the development of synthetic Nurr1 modulators [21]. Potent Nurr1 agonists have been developed based on DHI [14, 16, 18, 22] and the antimalarial amodiaquine (AQ) [13, 23, 24, 25, 26] (e.g., **1**‐**3**, Scheme 1). Additionally, we discovered strong Nurr1 activation by the dihydroorotate dehydrogenase (DHODH) inhibitor vidofludimus (**4**) [27, 28], which has been evaluated in late‐stage clinical trials for multiple sclerosis (MS) [29, 30], and developed analogs [15, 27] (e.g., **4a**) with enhanced potency and selectivity. The available Nurr1 ligands have been employed as chemical tools to explore the receptor's biological role and for target validation in in vitro models [13, 26]. However, a Nurr1 modulator with optimized brain penetration for focused in vivo studies on Nurr1 activation in the CNS is lacking. Here, we optimized the scaffold of **4** to a Nurr1 agonist (**28**) with single‐digit nanomolar potency, favorable selectivity, and high brain exposure for in vivo studies of the CNS role of Nurr1.

## 2 Results and Discussion

The blood‐brain barrier (BBB) is a semipermeable cellular membrane that presents an obstacle to small molecules, preventing many xenobiotics from entering the CNS from the blood, thereby, protecting the brain [31]. Empirical rules derived from the features of known brain‐penetrant drugs [31, 32] imply that the presence of less than or equal to five N + O atoms or less than three H‐bond donor (HBD) centers, a *pK* *_a_* > 6.0, a topological polar surface area (TPSA) below 90 Å^2^, and a logP between 2 and 4 are indicative for the ability of a molecule to pass the BBB, i.e., CNS bioavailability. The Nurr1 agonist **4** (N + O = 4, HBD = 2, *pK* *_a_* = 3.1, log*P* = 3.8, TPSA = 75.6 Å^2^) [33] deviates from these features mainly by the presence of an acidic motif. In our endeavor to develop an improved brain‐penetrant Nurr1 agonist based on the vidofludimus (**4**) scaffold, we thus aimed to replace the carboxylic acid, while additionally tuning physicochemical features for brain penetration and on‐target potency. Our previous structure‐activity relationship (SAR) studies [15, 27, 28] on this chemotype demonstrated the importance of the carboxylic acid but indicated that the presence of an HBD, rather than acidity, was critical. We hypothesized that an amide analog of **4** with strong Nurr1 agonist potency and sufficient preference over DHODH might hence be suitable to study the effects of Nurr1 activation in the CNS in vivo.

Our initial efforts to develop a non‐acidic analog of **4** (Table 1) focused on the original scaffold. Its corresponding amide **5** retained submicromolar Nurr1 agonism and displayed improved preference over DHODH, but nevertheless, its potency and selectivity were too low to be considered as a tool to study Nurr1 biology. Attempts to enhance potency with structurally diverse *N*‐substituted amides (**6**‐**10**) were not productive. Cyanomethyl (**6**), 2,2,2‐trifluoroethyl (**7**), and cyclopropyl (**8**) groups, along with oxetane (**9**) and thietane (**10**) substitution, were not tolerated. The azetidine analog **11** exhibited weak Nurr1 agonism, but its fluorination (**12**) was not tolerated, and also the *N*‐hydroxy amide **13** activated Nurr1 but lacked sufficient potency for further consideration.

**TABLE 1:** Initial attempts to replace the carboxylate motif in 4 by an amide.

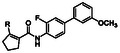			
ID	R=	EC_50_ (Nurr1) (max. act.)^a^	IC_50_ (DHODH)^b^
**4**	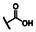	0.4 ± 0.2 µM (3.1 ± 0.4‐fold)^c^	0.61 ± 0.07 µM^c^
**5**	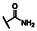	0.50 ± 0.03 µM (1.71 ± 0.02‐fold)	2.6 ± 0.5 µM
**6**	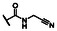	>3 µM^d^	19 ± 2% inhibition at 100 µM
**7**	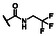	>3 µM^d^	14 ± 6% inhibition at 100 µM
**8**	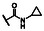	>3 µM^d^	39 ± 14% inhibition at 100 µM
**9**	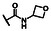	>3 µM^d^	34 ± 10% inhibition at 100 µM
**10**	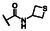	>3 µM^d^	43 ± 9% inhibition at 100 µM
**11**	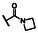	2.3‐fold act. at 3 µM	26 ± 4 µM
**12**	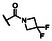	>3 µM^d^	7 ± 4% inhibition at 100 µM
**13**	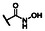	1.8 ± 0.1 µM (2.4 ± 0.1‐fold)	7.5 ± 0.5 µM

a Nurr1 agonism (mean±S.E.M., *n* = 3) was determined in a Gal4 hybrid reporter gene assay [34].

b DHODH inhibition (mean±S.E.M., *n *≥ 3) was determined in a colorimetric assay on recombinant human protein [35].

c Data for **4** from ref [27].

d Compounds were tested only up to 10 µM.

These initial SAR results demonstrated that an amide could be tolerated to replace the carboxylic acid in vidofludimus‐derived Nurr1 agonists, but this structural modification was accompanied by a loss in Nurr1 agonist potency and efficacy, and further *N*‐substitution of the amide mostly abolished activity on Nurr1. To design an amide analog activating Nurr1 with low nanomolar potency, we explored other potency‐driving structural variations from our previous SAR studies [27, 28] (Table 2). We have observed previously that deuteration of the methoxy group in the terminal anisole motif (**14**) enhanced Nurr1 agonist potency by a factor of five, potentially due to a subtle isotope‐dependent shift in noncovalent ligand‐target interactions [37, 38, 39, 40]. Additionally, fluorination of the central aromatic ring (**16**) emerged as a favored motif, further boosting potency. Despite being slightly less potent on Nurr1 than the carboxylic acids (**14** and **16**), the amides **15** and **17** complied with the SAR and benefited from deuteration and fluorination. Thus, we retained these structural modifications in further optimization.

**TABLE 2:** Incorporation of potency‐driving structural modifications in unsubstituted amides.

ID	structure	EC_50_ (Nurr1) (max. act.)^a^	IC_50_ (DHODH)^b^
**4**	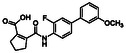	0.4 ± 0.2 µM (3.1 ± 0.4‐fold)^c^	0.61 ± 0.07 µM^c^
**5**	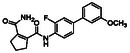	0.50 ± 0.03 µM (1.7 ± 0.1‐fold)	3.8 ± 2.0 µM
**14**	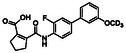	0.079 ± 0.001 µM (2.6 ± 0.1‐fold)^c^	0.4 ± 0.1 µM^c^
**15**	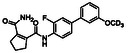	0.32 ± 0.03 µM (2.2 ± 0.1‐fold)	15 ± 2 µM
**16**	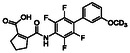	0.020 ± 0.004 µM (1.9 ± 0.1‐fold)^d^	0.008 ± 0.002 µM^e^
**17**	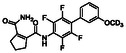	0.044 ± 0.003 µM (2.1 ± 0.1‐fold)	0.25 ± 0.03 µM

a Nurr1 agonism (mean±S.E.M., *n* = 3) was determined in a Gal4 hybrid reporter gene assay [34].

b DHODH inhibition (mean±S.E.M., *n *≥ 3) was determined in a colorimetric assay on recombinant human protein [35].

c Data from ref [27].

d Data from ref [15].

e Data from ref [36].

Previous SAR studies on **4** and analogs have mainly focused on the terminal methoxyphenyl group and the central aromatic ring [27], while the potential of variations in the cyclopentene carboxylic acid residue to enhance potency has not been systematically explored. Incorporation of an oxygen atom in this motif in the dihydrofuran analog **18** was not favored [15, 27], but aromatization to the furan **19** slightly enhanced Nurr1 agonist potency compared to the cyclopentene analog **16,** suggesting aromatic groups as favored (Table 3).

**TABLE 3:** Variation of the cyclopentene carboxylic acid motif.

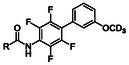			
ID	R=	EC_50_ (Nurr1) (max. act.)^a^	IC_50_ (DHODH)^b^
**16**	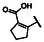	0.020 ± 0.004 µM (1.88 ± 0.07‐fold)	0.008 ± 0.002 µM^c^
**18**	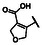	0.48 ± 0.06 µM (2.48 ± 0.09‐fold)	0.058 ± 0.016 µM^c^
**19**	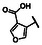	0.010 ± 0.003 µM (2.2 ± 0.4‐fold)	0.015 ± 0.002 µM^c^
**20**	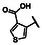	0.0025 ± 0.0001 µM (2.20 ± 0.08‐fold)	0.0022 ± 0.0002 µM^c^
**21**	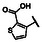	0.005 ± 0.002 µM (2.2 ± 0.1‐fold)^d^	0.0019 ± 0.0005 µM^d^
**22**	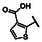	0.039 ± 0.005 µM (2.0 ± 0.1‐fold)	0.003 ± 0.002 µM
**23**	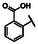	0.06 ± 0.02 µM (2.1 ± 0.2‐fold)^d^	0.015 ± 0.003 µM^d^
**24**	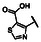	0.0033 ± 0.0002 µM (1.93 ± 0.04‐fold)	0.0010 ± 0.0006 µM
**25**	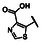	0.14 ± 0.02 µM (2.11 ± 0.09‐fold)	0.003 µM^e^
**26**	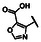	1.4 ± 0.1 µM (1.75 ± 0.04‐fold)	0.019 ± 0.006 µM
**27**	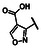	0.31 ± 0.04 µM (1.8 ± 0.1‐fold)	0.032 ± 0.005 µM

a Nurr1 agonism (mean±S.E.M., *n* = 3) was determined in a Gal4 hybrid reporter gene assay [34].

b DHODH inhibition (mean±S.E.M., *n *≥ 3) was determined in a colorimetric assay on recombinant human protein [35].

c Data from ref [36].

d Data from ref [15].

e *n* = 1 (**25** was unstable and tended to decarboxylate [41]).

The corresponding 3,4‐substituted thiophene **20** indeed gained further in potency, and the isomeric thiophene derivatives **21** and **22** were highly active too. The benzoic acid analog **23,** despite being a very potent Nurr1 agonist, was substantially less active than the thiophene **20**. These results highlighted a preference for aromatic five‐membered rings and prompted us to study further sulfur‐containing heterocycles, which revealed a distinctive SAR with pronounced differences between isomeric rings. The thiazole‐5‐carboxylic acid **24** exhibited very potent Nurr1 agonism resembling the thiophenes **20**–**22**, while the isomeric thiazole‐4‐carboxylic acid **25** was markedly less active. This drop in potency may indicate an impact of regiochemistry, but the limited stability of thiazole‐4‐carboxylic acid due to decarboxylation [41] prevented full interpretation. The oxazole‐5‐carboxylic acid analog **26** of the preferred stable thiazole isomer and the corresponding isoxazole (**27**) were detrimental for Nurr1 agonism, highlighting sulfur‐containing five‐membered rings as favored. Also, considering the presumably negative impact of additional heteroatoms on brain penetration [32], we thus focused on **20** for further SAR evaluation.

Deuteration of the methoxy motif (**14**), incorporation of additional fluorine substituents (**16**), and a scaffold hop from cyclopentene to thiophene (**20**) together enhanced Nurr1 agonist potency by more than 100‐fold compared to **4**, which seemed sufficient to compensate for the loss in activity observed for the amide group. With an EC_50_ of 7 nM, the amide analog **28** of **20** indeed retained strong potency on Nurr1 and appeared suitable as a potential brain‐penetrant agonist (Table 4). Concomitantly, **28** displayed markedly reduced DHODH inhibition compared to **20** and, hence, a favorable preference for Nurr1. Thiophene carboxamides thus seemed promising, prompting us to explore modifications of the amide group. As observed with the original scaffold (**5**–**13**), the SAR in this region was strict and most *N*‐substituents were detrimental. Increasing alkylation of the amide adversely affected Nurr1 activation, with the monomethyl (**29**), dimethyl (**30**), iso‐ (**31**), and cyclopropylamides (**32**) being markedly less potent on Nurr1 than the primary amide **28**. Installation of oxetane (**33**) and azetidine (**34**) substituents was also not favored, though both compounds retained submicromolar Nurr1 agonism and strong preference over DHODH. Difluoro (**35**) and hydroxy (**36**) substitutions on the azetidine were tolerated but failed to improve the activity profile. Hydrophobic mono‐, di‐, and trifluoroethyl motifs (**37**‐**39**), possibly enhancing brain penetration, also preserved submicromolar potency and favorable selectivity over DHODH, while propynyl (**40**) and cyanomethyl (**41**) substituents were tolerated but diminished Nurr1 preference. The homologous cyanoethyl derivative **42** displayed markedly reduced potency on Nurr1, while the cyanamide **43** was a highly potent Nurr1 agonist and DHODH inhibitor.

**TABLE 4:** Evaluation of thiophene carboxamides.

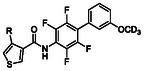			
ID	R=	EC_50_ (Nurr1) (max. act.)^a^	IC_50_ (DHODH)^b^
**20**	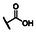	0.0025 ± 0.0001 µM (2.20 ± 0.08‐fold)	0.0022 ± 0.0002 µM
**28**	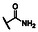	0.007 ± 0.003 µM (1.9 ± 0.1‐fold)	0.23 ± 0.08 µM
**29**	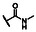	0.8 ± 0.1 µM (1.84 ± 0.07‐fold)	16 ± 2 µM
**30**	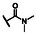	>3 µM^c^	37 ± 7 µM
**31**	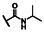	>3 µM^c^	4.2 ± 0.3 µM
**32**	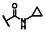	>3 µM^c^	2.4 ± 0.9 µM
**33**	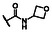	0.99 ± 0.05 µM (1.82 ± 0.05‐fold)	7.5 ± 0.3 µM
**34**	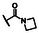	0.4 ± 0.1 µM (2.2 ± 0.2‐fold)	14.6 ± 0.4 µM
**35**	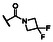	1.0 ± 0.2 µM (2.3 ± 0.3‐fold)	12 ± 1 µM
**36**	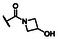	0.5 ± 0.1 µM (2.0 ± 0.1‐fold)	16.0 ± 0.3 µM
**37**	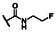	0.54 ± 0.09 µM (2.4 ± 0.3‐fold)	0.25 ± 0.08 µM
**38**	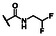	1.2 ± 0.2 µM (1.8 ± 0.1‐fold)	28.4 ± 0.8 µM
**39**	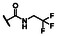	>3 µM^c^	43.6 ± 0.8% inhibition at 100 µM
**40**	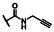	0.5 ± 0.1 µM (2.3 ± 0.1‐fold)	2.0 ± 0.3 µM
**41**	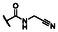	0.24 ± 0.02 µM (2.1 ± 0.1‐fold)	0.28 ± 0.03 µM
**42**	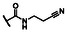	>3 µM^c^	10.8 ± 0.8 µM
**43**	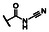	0.005 ± 0.001 µM (1.8 ± 0.1‐fold)	0.004 ± 0.001 µM

a Nurr1 agonism (mean±S.E.M., *n* = 3) was determined in a Gal4 hybrid reporter gene assay [34].

b DHODH inhibition (mean±S.E.M., *n* ≥ 3) was determined in a colorimetric assay on recombinant human protein [35].

c Compounds were tested only up to 10 µM.

Considering the desired profile of high Nurr1 agonist potency, selectivity over DHODH, and favorable features for brain penetration, the primary amide **28** thus emanated from this restrictive SAR as the most promising descendant of **4** and its derivatives. Eventually, we explored the incorporation of other favored modifications in the scaffold with the primary amide group (Table 5). A 4,4‐difluorocyclopentene (**44**) emerged from our previous SAR evaluation [27] of **4** as a viable alternative to the cyclopentene or thiophene motifs. Combination of the 4,4‐difluorocyclopentene with the primary amide and the central tetrafluorobenzene in **45** provided a potent and selective Nurr1 agonist that was only slightly less active than **28**. Previous studies [27] additionally revealed a propynyloxy substituent (**46**) as a highly favored replacement for the methoxy group in **4**. Installation of the thiophene‐3‐carboxamide (**47**) in this scaffold reduced Nurr1 agonism, but **47** nevertheless retained submicromolar Nurr1 agonism and strong selectivity over DHODH.

**TABLE 5:** Structural fusion of the favored carboxamide motif with potency‐driving modifications in the scaffold.

ID	structure	EC_50_ (Nurr1)^a^ (max. act.)	IC_50_ (DHODH)^b^
**28**	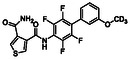	0.007 ± 0.003 µM (1.9 ± 0.1‐fold)	0.23 ± 0.08 µM
**44**	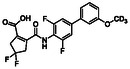	0.3 ± 0.2 µM(3.7 ± 0.8‐fold)^c^	0.013 ± 0.001 µM^c^
**45**	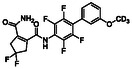	0.026 ± 0.001 µM (1.9 ± 0.1‐fold)	0.533 ± 0.009 µM
**4a**	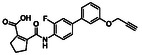	0.11 ± 0.05 µM (6.2 ± 0.4‐fold)^c^	1.7 ± 0.4 µM^c^
**47**	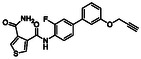	0.5 ± 0.1 µM (2.1 ± 0.1‐fold)	21 ± 1 µM

a Nurr1 agonism (mean±S.E.M., *n* = 3) was determined in a Gal4 hybrid reporter gene assay [34].

b DHODH inhibition (mean±S.E.M., *n *≥ 3) was determined in a colorimetric assay on recombinant human protein [35].

c Data from ref [27].

**FIGURE 1:**
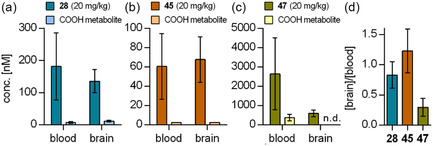
Comparative evaluation of blood and brain levels of **28** (a), **45** (b), **47** (c) in Sprague Dawley (SD) rats, and the corresponding brain‐to‐blood ratios (d). The compounds were administered p.o. at a 20 mg/kg dose each, and concentrations in blood and brain were determined 2 h after application. Data are the mean ± SD, *n* = 3.

With submicromolar potency on Nurr1, >30‐fold selectivity over DHODH, and chemical diversity within the studied scaffold, the amides **28**, **45**, and **47** appeared most promising as brain‐penetrant Nurr1 agonist candidates, prompting us to determine their CNS bioavailability in vivo. Preliminary comparative pharmacokinetic (PK) evaluation of **28**, **45**, and **47** in rats (Figure 1) indicated different PK properties after oral application of a 20 mg/kg dose. **47** displayed the highest blood concentration but a poor brain‐to‐blood ratio 2 h after application. **45** achieved only low blood and brain concentrations (<100 nM) and thus insufficient exposure despite a high brain‐to‐blood ratio. **28** offered the best overall PK profile with intermediate exposure and a favorable brain‐to‐blood ratio. The acid metabolites of the amides **28**, **45**, and **47** were not detected in relevant concentrations in both blood and brain, supporting our approach of employing a primary amide motif for brain penetration.

**FIGURE 2:**
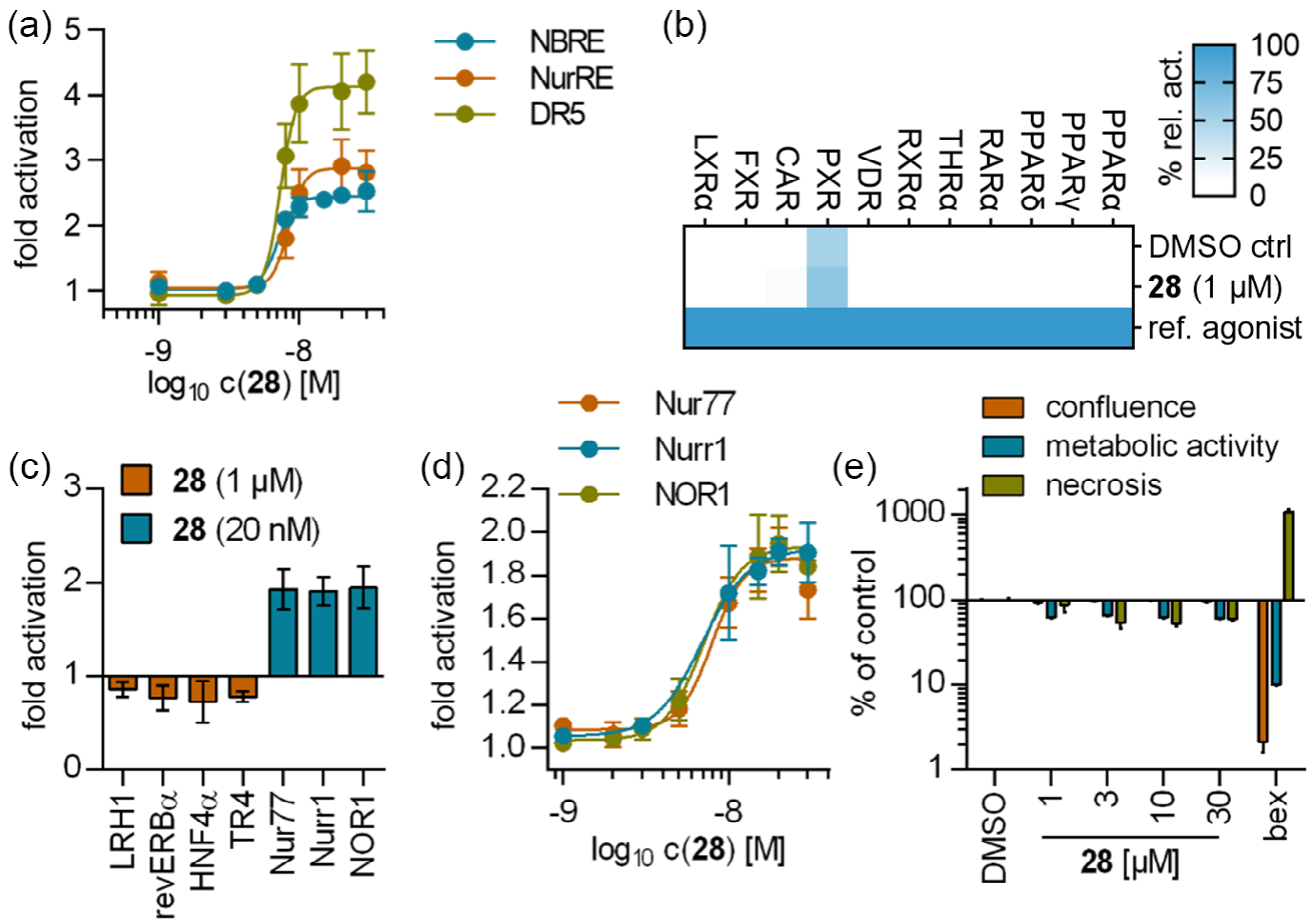
In vitro profiling of **28**. (a) **28** activated full‐length human Nurr1 on the response elements for the Nurr1 monomer (NBRE, EC_50_ = 0.0070 ± 0.0007 µM), the Nurr1 homodimer (NurRE, EC_50_ = 0.008 ± 0.001 µM), and the Nurr1:RXR heterodimer (DR5, EC_50_ = 0.0073 ± 0.0007 µM). Data are the mean ± S.E.M., *n *≥ 3. Activation of Nurr1 REs was studied with minor receptor overexpression. (b) Selectivity profiling in a panel of lipid‐sensing and promiscuous nuclear receptors revealed no activity of **28**. The heat map shows the mean relative activation compared to reference agonists; *n* = 3. (c) **28** was selective for NR4A receptors over other NRs with constitutive activator (LRH1, HNF4α) and repressor (revERBα, TR4) properties. Data are the mean ± S.E.M., *n* = 3. (d) **28** activated all NR4A receptors with similar potencies. Data are the mean ± S.E.M., *n* = 3. (e) **28** showed no toxicity in a multiplex toxicity assay monitoring confluence, metabolic activity, and necrosis in HEK293T cells. Bexarotene (100 µM) as a positive control. Data are the mean ± S.E.M., *n* = 4.

The amide **28** thus qualified as the most promising brain‐targeted Nurr1 agonist with brain concentrations exceeding the EC_50_ for Nurr1 activation by more than 10‐fold 2 h after oral dosing. Testing of **28** in reporter gene assays for activation of the human Nurr1 response elements NBRE (Nurr1 monomer), NurRE (Nurr1 homodimer), and DR5 (Nurr1:RXR heterodimer) demonstrated similar potency on all oligomers (Figure 2a), supporting Nurr1 agonism in native cellular settings. Direct interaction of **28** with the Nurr1 LBD was validated by isothermal titration calorimetry (ITC; Figure S1), which confirmed high‐affinity binding (K_d_ < 0.1 µM), and profiling in uniform hybrid reporter gene assays for a representative panel of nuclear receptors demonstrated selective agonism on Nurr1 and the related NR4A receptors Nur77 and NOR1 (Figure 2b,c). Dose‐response characterization showed similar potency of **28** on all NR4A receptors (Figure 2d), which is typical for Nurr1 agonists [21]. Due to species differences of DHODH but not Nurr1 in rats and lower potency on rDHODH (IC_50_ > 100 µM), **28** displayed improved Nurr1 selectivity in rats for in vivo applications. A multiplex toxicity assay in HEK293T cells additionally revealed no cytotoxic effects of **28** up to 30 µM (Figure 2e). Overall, this favorable profile supported the suitability of **28** as a chemical tool for biological studies.

## 3 Conclusion

Nurr1 is an emerging target for neurodegenerative disease treatment based on its neuroprotective potential and reports of dysfunction in AD and PD. Nurr1 modulators, from screening campaigns and early ligand development, have been studied in animal models of neurodegeneration with encouraging results [42]. However, several early Nurr1 ligand tools were devalidated [21], putting some putative in vivo effects of Nurr1 modulation into question. New chemical tools are needed to further explore and validate the transcription factor as a target, especially in animal models. The brain‐penetrant Nurr1 agonist **28** is thus an important addition to the available collection of chemical tools to study the receptor's therapeutic potential in vivo.

## 4 Chemistry

**SCHEME 2:**
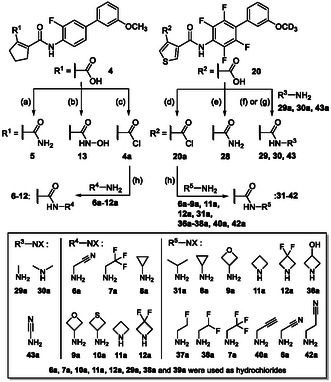
Synthesis of amides **5**–**13** and **28**–**43** ^a^. ^a^Reagents and Conditions: (a) NH_4_Cl, HATU, Hünig's base, DMF, rt, 4 h, 16%; (b) CDI, THF, rt, 1 h, then H_2_NOH•HCl, rt, overnight, 34%; (c) oxalyl chloride, cat. DMF, CH_2_Cl_2_, 0°C to 50°C, 1 h, quantitative; (d) oxalyl chloride, cat. DMF, CH_2_Cl_2_, 0°C to 50°C, 1 h, 96%; (e) NH_4_Cl, DMAP, EDC•HCl, DMF, 60°C, overnight, 50%; (f) **29a**/**30a**, Hünig's base, HATU, DMF, rt, 10 min, then addition of R^3^NH_2_, 5°C to rt, overnight, 59%–89%; (g) **43a**, NMP, EDC•HCl, THF, rt, 4 h, 24%; (h) R^4^‐NH_2_ or R^5^‐NH_2_, Hünig's base, THF/DMF, 5°C to rt, overnight, 9%–99%.

The synthesis of compounds **4**, **14**, **16**, **18–21**, **23**, **44**, and **46** has been described previously [15, 27, 36]. The amides **5–** **13** and **29–** **43** were synthesized according to Scheme 2. The primary amides **5** and **28** were prepared by treating the corresponding carboxylic acids **4** and **20** with ammonium chloride and suitable coupling reagents (HATU/Hünig's base for **5** and EDC/DMAP for **28**) in DMF. The *N*‐hydroxy amide **13** was obtained by reacting carboxylic acid **4** after CDI activation with hydroxylamine hydrochloride in THF, and reaction of **20** with cyanamide, NMP, and EDC in THF yielded **43**. The methylated amides **29** and **30** were synthesized by treating the carboxylic acid **20** with methylamine hydrochloride **29a** or a solution of dimethylamine in ethanol **30a** in the presence of Hünig's base and HATU in DMF. Other substituted amides were prepared via the acid chlorides **4a** and **20a,** which were formed by the treatment of the corresponding carboxylic acids **4** and **20** with oxalyl chloride in CH_2_Cl_2_ and DMF. Amides **6–** **12** and **31–** **42** were obtained by reacting the acid chloride **4a** or **20a** with the respective amine (hydrochloride) **6a–** **12a**, **31a, 36a–38a, 40a,** and **42a** in the presence of Hünig's base in THF and DMF overnight.

**SCHEME 3:**
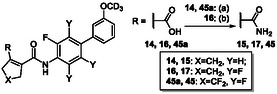
Synthesis of primary amides **15**, **17**, and **45** ^a^. ^a^Reagents and Conditions: (a) Hünig's base, HATU, DMF, rt, 10 min, then addition of ammonia (aqueous, 25%), 5°C to rt, overnight, 60%–78%; (b) NMI, HATU, rt, 30 min, then addition of ammonia (aqueous, 25%), MeCN, 5°C to rt, 16 h, 87%.

The primary amides **15**, **17**, and **45** were obtained from the respective carboxylic acids **14**, **16**, and **45a** [36] by treatment with aqueous ammonia and Hünig's base/HATU (**14**, **45a**) or NMI/HATU (**16**), according to Scheme 3.

**SCHEME 4:**
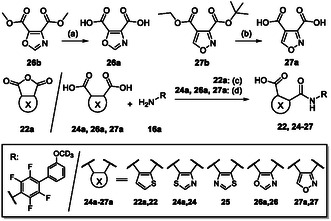
Synthesis of carboxylic acids **22**, **24**–**27** ^a^. ^a^Reagents and Conditions: (a) LiOH (aqueous, 2M), THF/MeOH, 0°C, 1 h, 71%; (b) HCl (10%), AcOH, 70°C, 2 h, 61%; (c) toluene, reflux, 15 h, 5%; (d) SOCl_2_, CH_2_Cl_2_ (**24a**, **27a**) or oxalyl chloride, THF (**26a**), 0°C, 2 h, evap.; **16a**, NaH, THF, 0°C, 10–30 min, then addition of acid chloride, 0°C to rt, 2–16 h, 3%–20%.

The heterocyclic carboxylic acids **22** and **24–** **27** were prepared according to Scheme 4. **22** was synthesized by reacting thiophene‐2,3‐dicarboxylic acid anhydride (**22a**) with aniline **16a** [36] in toluene. Oxazole‐4,5‐dicarboxylic acid (**26a**) and isoxazole‐4,5‐dicarboxylic acid (**27a**) were obtained by basic (**26a**) or acidic (**27a**) ester hydrolysis of the respective alkylesters **26b** and **27b**. Amide formation of the dicarboxylic acids **24a**, **26a**, and **27a** with aniline **16a** via in situ acid chloride formation with thionyl chloride in CH_2_Cl_2_ (**24a**, **27a**) or oxalyl chloride in THF (**26a**) yielded the heterocyclic maleamic acid derivatives **24–** **27**.

**SCHEME 5:**
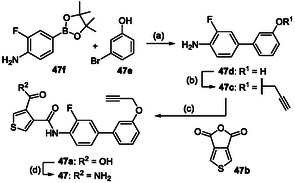
Synthesis of **47** ^a^. ^a^Reagents and Conditions: (a) Pd(dppf)Cl_2_, Cs_2_CO_3_, 1,4‐dioxane/H_2_O (10:1), 90°C, 16 h, 83%; (b) 3‐bromoprop‐1‐yne, K_2_CO_3_, DMF, rt, 6 h, 50%; (c) MeCN, 60°C, 2 h, 78%; (d) NH_4_Cl, HATU, Hünig's base, DMF, rt, overnight, 50%.

The propynyl ether derivative **47** was prepared over four steps according to Scheme 5. Suzuki coupling of the fluorinated aminobenzene boronic acid pinacol ester **47f** with 3‐bromophenol **47e** using Pd(dppf)Cl_2_ in 1,4‐dioxane/H_2_O provided the biphenyl **47d**. Subsequent Williamson ether synthesis with 3‐bromoprop‐1‐yne and K_2_CO_3_ in DMF afforded **47c**. Reacting aniline **47c** with thiophene‐3,4‐dicarboxylic acid anhydride (**47b**) in acetonitrile yielded carboxylic acid **47a**, from which **47** was available by amination with ammonium chloride, HATU, and Hünig's base in DMF.

## 5 Experimental Procedures

### 5.1 Chemistry

*General*. All chemicals were of reagent grade, purchased from commercial sources, and used without further purification unless otherwise specified. All reactions were conducted in oven‐dried glassware under Ar or N_2_ atmosphere and in absolute solvents (where appropriate). Other solvents, especially for work‐up procedures, were of reagent grade. Compounds were purified with a Biotage Isolera One combiflash chromatography system (SEPAFLASH, 40–63 Å) or Büchi Reveleris Prep system with FlashPure cartridges (EcoFlex Silica 50 µm irregular 80 g, Reveleris HP Silica 20 µm 40 g, or Reveleris HP Silica 20 µm 12 g) and with the solvent mixtures specified in the corresponding experiment. Preparative HPLC was performed using a combiflash reversed‐phase chromatography (C18), Boston ODS 40 g Flash, 35mL–50mL/min at 200 psi, with gradient A: 0.1% TFA in water, 10%–100% MeCN. Alternatively, preparative HPLC was performed on a Büchi Pure C‐850 FlashPrep system and C18 column [10 µm, 250 x 10.0 ID mm, or 150 x 30.0 ID mm] using gradient B: 10%–90% MeOH gradient in water or on Xtimate Prep C18 10 µm 21.2 x 250 mm using water (10 mM NH_4_CO_3_) and MeCN 25% to 55% as a gradient (gradient C). Mass spectra were obtained on a puriFlash‐CMS system (Advion) using atmospheric pressure chemical ionization (APCI), an Ion Trap Esquire 3000+ instrument (Bruker Corporation, Billerica, MA, USA) using electrospray ionization (ESI; liquid chromatography‐mass spectrometry (LCMS)‐ESI), or using a *Waters Acquity SQ Detector* coupled to a UPLC system. The instrument was operated in electrospray ionization (ESI) mode in both positive and negative ionization modes. Data acquisition and processing were performed using Waters MassLynx software. Using a [BEH C18, 1.7 µm, 2.1 × 50 mm], with a mobile phase of [water/acetonitrile + 0.1% formic acid] at a flow rate of [0.5 mL/min], gradient: 2%–98% B in 6.0 min; oven temperature: 10°C; mass range: 110–1000; detection: UV (214 nm, 254 nm) and MS (ESI, Pos mode, 2–2000 Da (amu)). NMR spectra were recorded on Bruker Avance 300 MHz, 400 MHz, or 500 MHz spectrometers equipped with CryoProbe Prodigy broadband probe (Bruker) or a Magritek Spinsolve 80 spectrometers (80 MHz). Chemical shifts are reported in *δ* values (ppm), coupling constants (*J*) in Hertz (Hz). Signals are described as br for broad. Purity of all compounds was analyzed on an Agilent Technologies 1200 Series machine under the following conditions: LC‐Mass Method 1: column: Sunfire C18, 4.6*50 mm, 3.5 µm; mobile phase: A: water (0.01% TFA), B: MeCN (0.01% TFA); gradient: 5%–95% B in 1.5 min; flow rate: 2.0 mL/min; oven temperature: 50°C; mass range: 110–1000; detection: UV (214 nm, 254 nm); or LC‐Mass Method 2: column: Xbridge C18(2) (4.6*50 mm, 3.5 µm); mobile phase: A: H_2_O (10 mmol NH_4_HCO_3_), B: MeCN; elution program: gradient from 10% to 95% of B in 1.5 min at 1.8 mL/min; temperature: 50ºC; detection: UV (214 nm, 254 nm) and MS (ESI, Pos mode, 103 to 800 amu). CD_3_I used for deuteration had ≥99% isotopic purity; the deuterated products had ≥98% isotopic purity according to NMR. All compounds for biological testing had a purity >95% based on the 254 nm ultraviolet (UV) trace. NMR and LCMS/UV data are provided in the Supporting Information.

*General procedure for amide coupling of amines with acid chlorides* (*GP1*)*.* To a stirred solution of an acid chloride (1.0 eq) in THF/DMF (5 mL; 2:1) at 5°C were added Hünig's base (1.5 to ≈ 3 eq) and the respective amine (optionally as hydrochloride). The mixture was stirred at rt overnight, diluted with water, and extracted with EtOAc three times. The combined organic layer was washed with brine, dried over anhydrous Na_2_SO_4_, filtered, concentrated under reduced pressure, and purified by preparative HPLC using a 10% to 90% MeOH gradient in water (gradient B).

*General procedure for amide coupling of amines with carboxylic acid* **20a** (*GP2*)*.* To a stirred solution of the carboxylic acid **20a** (1.0 eq.) in DMF was added Hünig's base (≈ 3 equiv), followed by HATU (1.2 equiv). The mixture was stirred at rt for 10 min, followed by the addition of the respective amine (optionally as hydrochloride) at 5°C. The mixture was stirred at rt overnight, diluted with water, and extracted with EtOAc three times. The combined organic layer was washed with brine, dried over Na_2_SO_4_, filtered, concentrated under reduced pressure, and purified with the method stated in the respective example.

*2‐*[(*3‐Fluoro‐3′‐methoxy‐*[*1*,*1′‐biphenyl*]*‐4‐yl*)*carbamoyl*]*cyclopent‐1‐ene‐1‐carbonyl chloride* (***4a***)*.* To a stirred mixture of **4** (20 g, 56 mmol) in anhydrous CH_2_Cl_2_ (150 mL) and DMF (three drops), oxalyl chloride (8.0 mL, 93 mmol) was added dropwise at 0°C–5°C over 1 min. The mixture was stirred at rt for 30 min, then heated to 50°C and stirred for another 30 min. Initially a suspension, the mixture gradually changed to a colorless, and then a gold‐colored solution. The mixture was concentrated in vacuo, and the remaining solid residue was dissolved in CH_2_Cl_2_ (50 mL) before being concentrated again in vacuo. The resulting solid was treated with petroleum ether (20 mL), filtered, and washed with petroleum ether. The solid was dried on a rotary evaporator at 50°C, yielding crude acid chloride **4a** (21 g, yield: quantitative) as a beige solid, which was used directly in the next reaction without further purification or characterization.

*N‐*(*3‐Fluoro‐3′‐methoxy‐*[*1*,*1′‐biphenyl*]*‐4‐yl*)*cyclopent‐1‐ene‐1*,*2‐dicarboxamide* (***5***)*.* To a solution of vidofludimus **4** (0.15 g, 0.42 mmol) in DMF (3 mL) were added NH_4_Cl (0.11 g, 2.1 mmol), HATU (0.19 g, 0.50 mmol), and Hünig's base (0.14 g, 1.1 mmol). The mixture was stirred at rt for 4 h, diluted with water, and extracted with EtOAc (3 x 20 mL). The combined organic layer was washed with brine, dried over Na_2_SO_4_, filtered, concentrated, and purified by preparative HPLC (gradient A) to obtain compound **5** (24 mg, yield: 16%) as a colorless solid. ^1^H NMR (400 MHz, CD_3_OD) **δ** = 8.21 (t, *J =* 8.4 Hz, 1H), 7.45–7.41 (m, 2H), 7.34 (t, *J = *8.2 Hz, 1H), 7.19–7.13 (m, 2H), 6.93–6.90 (m, 1H), 3.85 (s, 3H), 2.92–2.87 (m, 4H), 2.00–1.92 (m, 2H) ppm. ^13^C NMR (126 MHz, DMSO‐*d*_6_) *δ* = 168.2, 162.6, 159.8, 153.0 (d, *J = *244.9 Hz), 143.3, 139.9 (d, *J = *1.9 Hz), 139.5, 136.3 (d, *J = *7.1 Hz), 130.0, 126.0 (d, *J = *11.5 Hz), 122.5 (d, *J = *2.9 Hz), 122.5, 118.7, 113.4, 113.3 (d, *J = *20.0 Hz), 111.8, 55.2, 36.6, 36.3, 20.4 ppm. LCMS (ESI): *m/z* 355.2 ([M + H]^+^).

*N* ^1^ *‐Cyanomethyl‐N* ^2^ *‐*(*3‐fluoro‐3′‐methoxy‐*[*1*,*1′‐biphenyl*]*‐4‐yl*)*cyclopent‐1‐ene‐1*,*2‐dicarboxamide* (***6***)*.* Preparation according to GP1, using vidofludimus acid chloride **4a** (0.20 g, 0.54 mmol) and aminoacetonitril hydrochloride **6a** (75 mg, 0.81 mmol, 1.5 eq) to give compound **6** (0.11 g, yield: 52%) as a light‐yellow powder. ^1^H NMR (300 MHz, DMSO‐*d*_6_) ***δ*** = 11.07 (s, 1H), 8.88 (t, *J =* 5.4 Hz, 1H), 8.14 (t, *J =* 8.4 Hz, 1H), 7.63 (dd, *J =* 12.4, 2.1 Hz, 1H), 7.53 (dd, *J =* 8.5, 2.1 Hz, 1H), 7.37 (t, *J =* 7.9 Hz, 1H), 7.30–7.21 (m, 2H), 6.94 (ddd, *J =* 8.1, 2.5, 1.0 Hz, 1H), 4.23 (d, *J =* 5.5 Hz, 2H), 3.83 (s, 3H), 2.86–2.72 (m, 4H), 1.89 (p, *J =* 7.6 Hz, 2H) ppm. ^13^C NMR (126 MHz, DMSO‐*d*_6_) *δ* = 165.7, 163.5, 159.8, 153.6 (d, *J =* 245.4 Hz), 144.4, 139.9 (d, *J =* 1.9 Hz), 137.4, 137.1 (d, *J =* 7.1 Hz), 130.0, 125.4 (d, *J =* 11.6 Hz), 123.6, 122.5 (d, *J =* 2.8 Hz), 118.8, 117.2, 113.6 (d, *J =* 20.2 Hz), 113.4, 111.9, 55.2, 36.0, 35.0, 27.5, 21.1 ppm. LCMS (ESI): *m/z* 394.0 ([M + H]^+^).

*N* ^1^ *‐*(*3‐Fluoro‐3′‐methoxy‐*[*1*,*1′‐biphenyl*]*‐4‐yl*)*‐N* ^2^ *‐*(*2*,*2*,*2‐trifluoroethyl*)*cyclopent‐1‐ene‐1*,*2‐dicarboxamide* (***7***)*.* Preparation according to GP1, using vidofludimus acid chloride **4a** (0.20 g, 0.54 mmol) and 2,2,2‐trifluoroethan‐1‐amine hydrochloride **7a** (0.11 g, 0.81 mmol, 1.5 eq) to obtain compound **7** (0.10 g, yield: 43%) as a yellow powder. ^1^H NMR (80 MHz, DMSO‐*d*_6_) ***δ*** = 10.82 (s, 1H), 8.86 (s, 1H), 8.10 (t, *J =* 8.5 Hz, 1H), 7.79–7.17 (m, 5H), 6.93 (d, *J =* 7.3 Hz, 1H), 4.30–3.85 (m, 2H), 3.83 (s, 3H), 2.81 (t, *J =* 7.4 Hz, 4H), 1.89 (t, *J =* 7.6 Hz, 2H) ppm. ^13^C NMR (126 MHz, DMSO‐*d*_6_) *δ* = 166.2, 163.6, 159.8, 153.6 (d, *J =* 245.4 Hz), 143.3, 139.9 (d, *J =* 1.9 Hz), 138.5, 137.1 (d, *J =* 7.0 Hz), 130.0, 125.3 (d, *J =* 11.8 Hz), 124.6 (q, *J =* 279.2 Hz), 123.7, 122.5 (d, *J =* 2.8 Hz), 118.8, 113.5 (d, *J =* 20.4 Hz), 113.5, 111.9, 55.2, 39.6 (q, *J =* 33.7 Hz), 35.8, 35.3, 21.1 ppm. LCMS (ESI): *m/z* 437.1 ([M + H]^+^).

*N* ^1^ *‐Cyclopropyl‐N* ^2^ *‐*(*3‐fluoro‐3′‐methoxy‐*[*1*,*1′‐biphenyl*]*‐4‐yl*)*cyclopent‐1‐ene‐1*,*2‐dicarboxamide* (***8***)*.* Preparation according to GP1, using vidofludimus acid chloride **4a** (0.20 g, 0.54 mmol) and cyclopropylamine **8a** (57 µL, 0.81 mmol) to obtain compound **8** (0.14 g, yield: 66%) as a yellow powder. ^1^H NMR (80 MHz, DMSO‐*d*_6_) ***δ*** = 11.77 (s, 1H), 8.45–8.07 (m, 2H), 7.87–7.15 (m, 5H), 6.93 (d, *J =* 7.3 Hz, 1H), 3.76 (s, 3H), 2.82–2.64 (m, 5H), 1.86 (q, *J =* 7.3 Hz, 2H), 0.75–0.35 (m, 4H) ppm. ^13^C NMR (126 MHz, DMSO‐*d*_6_) *δ* = 167.2, 162.9, 159.8, 153.2 (d, *J =* 245.2 Hz), 142.3, 139.9 (d, *J =* 1.7 Hz), 139.5, 136.6 (d, *J =* 7.1 Hz), 130.0, 125.8 (d, *J =* 11.6 Hz), 122.9, 122.5 (d, *J =* 2.8 Hz), 118.8, 113.4 (d, *J =* 20.2 Hz), 113.4, 111.9, 55.2, 36.1, 36.0, 22.9, 20.7, 5.7 ppm. LCMS (ESI): *m/z* 393.0 ([M – H]^–^).

*N* ^1^ *‐*(*3‐Fluoro‐3′‐methoxy‐*[*1*,*1′‐biphenyl*]*‐4‐yl*)*‐N* ^2^ *‐*(*oxetan‐3‐yl*)*cyclopent‐1‐ene‐1*,*2‐dicarboxamide* (***9***)*.* Preparation according to GP1, using vidofludimus acid chloride **4a** (0.20 g, 0.54 mmol) and 3‐aminooxetane **9a** (56 µL, 0.81 mmol) to obtain compound **9** (0.12 g, yield: 55%) as a colorless powder. ^1^H NMR (80 MHz, DMSO‐*d*_6_) ***δ*** = 11.31 (s, 1H), 8.80 (d, *J =* 5.3 Hz, 1H), 8.09 (t, *J =* 8.5 Hz, 1H), 7.70–7.10 (m, 5H), 6.84 (d, *J =* 7.3 Hz, 1H), 5.90–4.30 (m, 5H), 3.74 (s, 3H), 3.06–2.60 (m, 4H), 1.77 (q, *J =* 7.4 Hz, 2H) ppm. ^13^C NMR (126 MHz, DMSO‐*d*_6_) δ = 165.5, 163.1, 159.8, 153.4 (d, *J =* 245.3 Hz), 142.8, 139.9 (d, *J =* 1.7 Hz), 139.1, 136.8 (d, *J =* 7.8 Hz), 130.0, 125.6 (d, *J =* 11.4 Hz), 123.2, 122.5 (d, *J =* 2.9 Hz), 118.8, 113.5 (d, *J =* 20.3 Hz), 113.4, 111.9, 76.5, 55.2, 44.4, 35.9, 35.8, 20.9 ppm. LCMS (ESI): *m/z* 411.1 ([M + H]^+^).

*N* ^1^ *‐*(*3‐Fluoro‐3′‐methoxy‐*[*1*,*1′‐biphenyl*]*‐4‐yl*)*‐N* ^2^ *‐*(*thietan‐3‐yl*)*cyclopent‐1‐ene‐1*,*2‐dicarboxamide* (***10***)*.* Preparation according to the GP1, using vidofludimus acid chloride **4a** (0.20 g, 0.54 mmol) and thietan‐3‐amine hydrochloride **10a** (29 mg, 0.54 mmol) to obtain compound **10** (84 mg, yield: 37%) as an off‐colorless powder. ^1^H NMR (300 MHz, DMSO‐*d*_6_) ***δ*** = 11.39 (s, 1H), 8.78 (d, *J =* 7.7 Hz, 1H), 8.18 (t, *J =* 8.4 Hz, 1H), 7.63 (dd, *J =* 12.5, 2.1 Hz, 1H), 7.52 (dd, *J =* 8.5, 2.1 Hz, 1H), 7.37 (t, *J =* 7.9 Hz, 1H), 7.30–7.19 (m, 2H), 6.93 (ddd, *J =* 8.0, 2.7, 1.0 Hz, 1H), 5.22–4.95 (m, 1H), 3.82 (s, 3H), 3.63–3.42 (m, 2H), 3.20 (td, *J =* 8.1, 1.7 Hz, 2H), 2.79 (d, *J =* 6.8 Hz, 4H), 1.85 (p, *J =* 7.6 Hz, 2H) ppm. ^13^C NMR (126 MHz, DMSO‐*d*_6_) *δ* = 164.4, 163.1, 159.8, 153.4 (d, *J =* 245.2 Hz), 143.1, 139.9 (d, *J =* 1.7 Hz), 139.0, 136.8 (d, *J =* 7.1 Hz), 130.0, 125.6 (d, *J =* 11.5 Hz), 123.2, 122.5 (d, *J =* 3.1 Hz), 118.8, 113.5 (d, *J =* 20.3 Hz), 113.4, 111.9, 55.2, 47.0, 35.9, 35.6, 34.2, 20.9 ppm. LCMS (ESI): *m/z* 425.0 ([M – H]^–^).

*2‐*(*Azetidine‐1‐carbonyl*)*‐N‐*(*3‐fluoro‐3′‐methoxy‐*[*1*,*1′‐biphenyl*]*‐4‐yl*)*cyclopent‐1‐ene‐1‐carboxamide* (***11***). Preparation according to GP1, using vidofludimus acid chloride **4a** (0.20 g, 0.54 mmol) and azetidine hydrochloride **11a** (56 µL, 0.81 mmol) to obtain compound **11** (83 mg, yield: 39%) as a colorless powder. ^1^H NMR (80 MHz, DMSO‐*d*_6_) ***δ*** = 10.69 (s, 1H), 8.01 (t, *J =* 8.4 Hz, 1H), 7.91–7.15 (m, 5H), 6.90 (d, *J =* 7.4 Hz, 1H), 4.31–3.79 (m, 7H), 2.72–1.74 (m, 8H) ppm. ^13^C NMR (126 MHz, DMSO‐*d*_6_) δ = 167.4, 162.8, 159.8, 153.9 (d, *J =* 245.6 Hz), 140.4, 140.2, 139.9 (d, *J =* 1.7 Hz), 137.2 (d, *J =* 7.2 Hz), 130.0, 125.4 (d, *J =* 11.8 Hz), 124.0, 122.5 (d, *J =* 3.0 Hz), 118.8, 113.6 (d, *J =* 20.6 Hz), 113.5, 111.9, 55.2, 50.8, 48.2, 35.5, 34.4, 21.8, 15.2 ppm. LCMS (ESI): *m/z* 395.1 ([M + H]^+^).

*2‐*(*3*,*3‐Difluoroazetidine‐1‐carbonyl*)*‐N‐*(*3‐fluoro‐3′‐methoxy‐*[*1*,*1′‐biphenyl*]*‐4‐yl*)*cyclopent‐1‐ene‐1‐carboxamide* (***12***)*.* Preparation according to GP1, using vidofludimus acid chloride **4a** (0.20 g, 0.54 mmol) and 3,3‐difluoroazetidin hydrochloride **12a** (0.14 g, 0.81 mmol) to obtain compound **12** (80 mg, yield: 35%) as a colorless powder. ^1^H NMR (300 MHz, DMSO‐*d*_6_) ***δ*** = 10.12 (s, 1H), 7.84 (t, *J =* 8.3 Hz, 1H), 7.64 (dd, *J =* 12.2, 2.0 Hz, 1H), 7.53 (dd, *J =* 8.4, 2.0 Hz, 1H), 7.38 (t, *J =* 7.9 Hz, 1H), 7.30–7.21 (m, 2H), 6.95 (ddd, *J =* 8.2, 2.6, 1.0 Hz, 1H), 4.45 (d, *J =* 43.8 Hz, 4H), 3.83 (s, 3H), 2.83–2.63 (m, 4H), 1.95 (p, *J =* 7.6 Hz, 2H) ppm. ^13^C NMR (126 MHz, DMSO‐*d*_6_) *δ* = 168.2, 163.0, 159.8, 154.7 (d, *J =* 246.2 Hz), 140.8, 139.9 (d, *J =* 2.0 Hz), 139.8, 138.1 (d, *J =* 7.4 Hz), 130.0, 125.3, 124.9 (d, *J =* 12.2 Hz), 122.5 (d, *J =* 2.9 Hz), 118.9, 116.2 (t, *J =* 271.6 Hz), 113.7 (d, *J =* 20.6 Hz), 113.6, 112.0, 62.2–61.1 (m), 60.3–59.3 (m), 55.2, 35.1, 33.8, 22.1 ppm. LCMS (ESI): *m/z* 431.1 ([M + H]^+^).

*N* ^1^ *‐*(*3‐Fluoro‐3′‐methoxy‐*[*1*,*1′‐biphenyl*]*‐4‐yl*)*‐N* ^2^ *‐hydroxycyclopent‐1‐ene‐1*,*2‐dicarboxamide* (***13***)*.* To a solution of vidofludimus **4** (0.10 g, 0.28 mmol) in dry THF (2 mL) was added 1,1′‐carbonyldiimidazole (90 mg, 0.56 mmol). After 1 h at rt, H_2_NOH·HCl (39 mg, 0.56 mmol) was added, and the mixture was stirred at rt overnight, diluted with water, and extracted with EtOAc (3 x 20 mL). The combined organic layer was washed with brine, dried over Na_2_SO_4_, filtered, concentrated under reduced pressure, and purified by preparative HPLC (gradient A) to afford compound **13** (35 mg, yield: 34%) as a colorless solid. ^1^H NMR (400 MHz, CD_3_OD) ***δ*** = 8.21–8.15 (m, 1H), 7.46–7.41 (m, 2H), 7.34 (t, *J =* 7.8 Hz, 1H), 7.20–7.14 (m, 2H), 6.91 (dd, *J =* 8.0, 2.1 Hz, 1H), 3.85 (s, 3H), 2.90–2.80 (m, 4H), 1.97–1.90 (m, 2H) ppm. ^13^C NMR (126 MHz, DMSO‐*d*_6_) *δ* = 163.1, 162.4, 159.8, 153.3 (d, *J =* 245.0 Hz), 140.7, 140.0 (d, *J =* 1.6 Hz), 138.9, 135.9 (d, *J =* 7.1 Hz), 130.0, 126.6 (d, *J =* 11.5 Hz), 122.8, 122.4 (d, *J =* 2.8 Hz), 118.7, 113.3, 113.3 (d, *J =* 20.3 Hz), 111.8, 55.2, 36.0, 35.7, 20.7 ppm. LCMS (ESI): *m/z* 371.1 ([M + H]^+^).

*N‐*[*3‐Fluoro‐3′‐*(^2^ *H*_3_)*‐methoxy‐*[*1*,*1′‐biphenyl*]*‐4‐yl*]*cyclopent‐1‐ene‐1*,*2‐dicarboxamide* (***15***)*.* Preparation according to GP2, using carboxylic acid **14** (0.15 g, 0.42 mmol) and 25% aqueous ammonia (0.5 mL) to obtain compound **15** (90 mg, yield: 60%) as a yellow solid after purification by flash column chromatography on silica gel (petroleum ether/EtOAc = 1:0 to 6:4). ^1^H NMR (80 MHz, DMSO‐*d*_6_) ***δ*** = 12.34 (s, 1H), 8.27 (t, *J =* 8.3 Hz, 1H), 7.87–7.17 (m, 7H), 6.94–6.83 (m, 1H), 2.78 (t, *J =* 7.5 Hz, 4H), 1.76 (q, *J =* 7.4 Hz, 2H) ppm. ^13^C NMR (126 MHz, DMSO‐*d*_6_) *δ* = 168.2, 162.6, 159.8, 153.0 (d, *J =* 244.7 Hz), 143.3, 139.9 (d, *J =* 2.0 Hz), 139.5, 136.3 (d, *J =* 7.1 Hz), 130.0, 126.0 (d, *J =* 11.4 Hz), 122.5 (d, *J =* 2.9 Hz), 122.5, 118.7, 113.4, 113.3 (d, *J =* 19.7 Hz), 111.8, 54.7–53.8 (m), 36.6, 36.3, 20.4 ppm. LCMS (ESI): *m/z* 358.1 ([M + H]^+^).

*N‐*[*2*,*3*,*5*,*6‐Tetrafluoro‐3′‐*(^2^ *H*_3_)*‐methoxy‐*[*1*,*1′‐biphenyl*]*‐4‐yl*]*cyclopent‐1‐ene‐1*,*2‐dicarboxamide* (***17***)*.* To a solution of acid **16** (0.25 g, 0.61 mmol) in MeCN (25 mL) was added *N*‐methylimidazole (0.17 g, 2.1 mmol), followed by HATU (0.20 g, 0.70 mmol). The mixture was stirred at rt for 30 min, after which 25% aqueous ammonia (0.5 mL) was added dropwise at 5°C. The mixture was stirred at rt for 16 h, diluted with water (100 mL), and concentrated under reduced pressure. The resulting precipitate was collected by filtration, washed with water (3 × 50 mL), and dried to afford product **17** (0.22 g, yield: 87%) as a colorless solid. ^1^H NMR (80 MHz, DMSO‐*d*_6_) ***δ*** = 12.20 (s, 1H), 8.11–7.67 (m, 2H), 7.65–7.29 (m, 1H), 7.24–6.89 (m, 3H), 2.81 (t, *J =* 7.2 Hz, 4H), 1.84 (q, *J =* 7.1 Hz, 2H) ppm. ^13^C NMR (126 MHz, DMSO‐*d*_6_) *δ* = 167.8, 162.7, 159.3, 144.5–142.3 (m), 143.1–140.8 (m), 141.4, 141.1, 129.9, 127.7, 122.2, 117.1 (t, *J =* 17.7 Hz), 116.3 (t, *J =* 15.2 Hz), 115.7, 114.9, 54.9–54.1 (m), 36.3, 36.0, 20.6 ppm. LCMS (ESI): *m/z* 412.1 ([M + H]^+^).

*4‐{*[*2*,*3*,*5*,*6‐Tetrafluoro‐3′‐*(^2^ *H*_3_)*‐methoxy‐*[*1*,*1′‐biphenyl*]*‐4‐yl*]*carbamoyl}thiophene‐3‐carbonyl chloride* (**20a**)*.* To a stirred mixture of carboxylic acid **20** (8.0 g, 19 mmol) and DMF (three drops) in anhydrous CH_2_Cl_2_ (25 mL) was added oxalyl chloride (3.0 mL, 35 mmol) at 0°C–5°C over 1 min. The mixture was stirred for 30 min at rt then for 30 min at 50°C. The mixture was concentrated under reduced pressure, and the remaining residue was coevaporated three times with anhydrous CH_2_Cl_2_ (25 mL) on a rotary evaporator. To the solid was added 20 mL of petroleum ether and collected by filtration, washed with petroleum ether, and dried in a vacuum to give the intermediate **20a** (8.0 g, yield: 96%) as a yellow solid, which was used in the next step without further purification or characterization.

*2‐{*[*2*,*3*,*5*,*6‐Tetrafluoro‐3′‐*(^2^ *H*_3_)*‐methoxy‐*[*1*,*1′‐biphenyl*]*‐4‐yl*]*carbamoyl}thiophene‐3‐carboxylic acid* (***22***)*.* A solution of thiophene‐2,3‐dicarboxylic anhydride **22a** (0.10 g, 0.65 mmol) and aniline **16a** (0.18 g, 0.66 mmol) in toluene (10 mL) was refluxed for 15 hr, cooled to rt, concentrated under reduced pressure, diltued with water (20 mL), and extracted with EtOAc (3 x 20 mL). The combined organic layer was washed with water, dried over anhydrous MgSO_4_, filtered, concentrated under reduced pressure, and purified by flash chromatography on silica gel (petroleum ether/EtOAc = 9:1) to afford compound **22** (13 mg, yield: 5%) as a colorless solid beside the other regioisomer (0.13 g). ^1^H NMR (500 MHz, DMSO‐*d*_6_) *δ* = 12.42 (s, 1H), 7.89 (d, *J =* 5.2 Hz, 1H), 7.58 (d, *J =* 5.2 Hz, 1H), 7.47 (t, *J =* 7.9 Hz, 1H), 7.16–7.06 (m, 3H) ppm. ^13^C NMR (126 MHz, DMSO‐*d*_6_) *δ* = 165.5, 159.4, 159.3, 144.6–142.4 (m), 142.6, 143.1–140.9 (m), 132.9, 131.3, 130.0, 129.9, 127.7, 122.2, 117.6 (t, *J =* 17.7 Hz), 116.1–115.7 (m), 115.8, 115.0, 55.1–53.8 (m) ppm. LCMS (ESI): *m/z* 428.9 ([M + H]^+^).

*4‐{*[*2*,*3*,*5*,*6‐Tetrafluoro‐3′‐*(^2^ *H*_3_)*‐methoxy‐*[*1*,*1′‐biphenyl*]*‐4‐yl*]*carbamoyl}thiazole‐5‐carboxylic acid* (***24***) and *5‐{*[*2*,*3*,*5*,*6‐Tetrafluoro‐3′‐*(^2^ *H*_3_)*‐methoxy‐*[*1*,*1′‐biphenyl*]*‐4‐yl*]*carbamoyl}thiazole‐4‐carboxylic acid* (***25***)*.* To a solution of thiazole‐4,5‐dicarboxylic acid **24a/25a** (0.10 g, 0.58 mmol) in dry CH_2_Cl_2_ (2 mL) was added SOCl_2_ (0.14 g, 1.2 mmol) at 0°C. The mixture was stirred at 0°C for 2 h and concentrated under vacuum to afford the crude acid chloride intermediate. To a solution of aniline **16a** (0.13 g, 0.47 mmol) in dry THF (2 mL) was added NaH (0.11 g, 60%wt, 2.8 mmol) at 0°C, and the mixture was stirred at 0°C for 30 min. Then, the acid chloride intermediate was added at 0°C, and the mixture was stirred at rt for 16 h, carefully quenched with saturated aq. NH_4_Cl and extracted with EtOAc (3 x 5 mL). The combined organic layer was dried over Na_2_SO_4_, filtered, concentrated under reduced pressure, and purified by preparative HPLC (gradient C) to give compound **24** (50 mg, yield: 20%) and compound **25** (26 mg, yield: 10%) as a yellow solid, respectively. **24**: ^1^H NMR (400 MHz, CD_3_OD) ***δ*** = 8.94 (s, 1H), 7.45–7.41 (m, 1H), 7.07–7.03 (m, 3H) ppm. ^13^C NMR (126 MHz, DMSO‐*d*_6_) *δ* = 162.4, 159.8, 159.7, 154.1, 146.6, 145.3, 145.1–142.8 (m), 143.6–141.3 (m), 130.3, 128.5, 122.8, 118.4 (t, *J =* 15.1 Hz), 116.8 (t, *J =* 17.9 Hz), 116.2, 115.3, 55.4–54.5 (m) ppm. LCMS (ESI): *m/z* 430.1 ([M + H]^+^). **25**: ^1^H NMR (400 MHz, CD_3_OD) *δ* = 9.03 (s, 1H), 7.45–7.41 (m, 1H), 7.06–7.04 (m, 3H) ppm. ^13^C NMR (126 MHz, DMSO‐*d*_6_) *δ* = 163.5, 159.3, 159.3, 154.7, 153.0–151.0 (m), 144.6–142.4 (m), 142.9–140.6 (m), 138.1–136.5 (m), 129.9, 128.0, 122.3, 118.0–117.4 (m), 116.8–116.1 (m), 115.8, 114.9, 54.9–53.9 (m) ppm. LCMS (ESI): *m/z* 430.2 ([M + H]^+^).

*4‐{*[*2*,*3*,*5*,*6‐Tetrafluoro‐3′‐*(^2^ *H*_3_)*‐methoxy‐*[*1*,*1′‐biphenyl*]*‐4‐yl*]*carbamoyl}oxazole‐5‐carboxylic acid* (***26***)*.* To a solution of intermediate **26a** (0.60 g, 3.8 mmol) in dry THF (10 mL) was added oxalyl chloride (0.96 g, 7.6 mmol) at 0°C. The mixture was stirred at 0°C for 2 h and concentrated under vacuum to afford the crude acid chloride intermediate. To a solution of aniline **16a** (1.1 g, 4.0 mmol) in dry THF (15 mL) was added NaH (0.79 g, 60%wt, 20 mmol) at 0°C. The mixture was stirred at 0°C for 30 min, then the acid chloride intermediate was added at 0°C. The mixture was stirred at 0°C for 2 h, carefully quenched with saturated aq. NH_4_Cl and extracted with EtOAc (3 x 50 mL). The combined organic layer was dried over Na_2_SO_4_, filtered, concentrated under reduced pressure, and purified by combiflash reversed‐phase flash chromatography (0.1% NH_4_HCO_3_ in water, 10% to 100% MeCN) to give compound **26** (50 mg, yield: 3%) as a colorless solid beside the other isomer (18 mg). ^1^H NMR (400 MHz, CD_3_OD) *δ* = 8.34 (s, 1H), 7.43 (dd, *J =* 7.8, 8.6 Hz, 1H), 7.06–7.04 (m, 3H) ppm. ^13^C NMR (126 MHz, DMSO‐*d*_6_) *δ* = 159.3, 158.7, 158.4, 150.9, 149.0, 144.6–142.4 (m), 143.1–140.8 (m), 132.9, 129.9, 127.9, 122.3, 117.1 (t, *J =* 14.9 Hz), 116.7 (t, *J =* 17.8 Hz), 115.8, 114.9, 54.9–54.1 (m) ppm. LCMS (ESI): *m/z* 414.1 ([M + H]^+^).

*Oxazole‐4*,*5‐dicarboxylic acid* (***26a***)*.* To a solution of dimethyl oxazole‐4,5‐dicarboxylate **26b** (1.0 g, 5.4 mmol) in THF (8 mL) and MeOH (8 mL) was added aqueous LiOH (2M, 10 mL). The mixture was stirred at 0°C for 1 h, acidified to pH 5 to 6 by the addition of 2N HCl. The mixture was purified by combiflash reversed‐phase chromatography (C18) (0.1% TFA in water, 10% to 100% MeCN) to give intermediate **26a** (0.60 g, yield: 71%) as a colorless solid. LCMS (ESI): *m/z* 158.1 ([M + H]^+^).

*3‐{*[*2*,*3*,*5*,*6‐Tetrafluoro‐3′‐*(^2^ *H*_3_)*‐methoxy‐*[*1*,*1′‐biphenyl*]*‐4‐yl*]*carbamoyl}isoxazole‐4‐carboxylic acid* (***27***)*.* To a solution of intermediate **27a** (0.20 g, 1.3 mmol) in dry CH_2_Cl_2_ (5 mL) was added SOCl_2_ (0.30 g, 2.5 mmol) at 0°C. The mixture was stirred at 0°C for 2 h and concentrated under vacuum to afford the crude acid chloride intermediate. To a solution of aniline **16a** (0.35 g, 1.3 mmol) in dry THF (5 mL) was added NaH (0.25 g, 60%wt, 6.4 mmol) at 0°C, and the mixture was stirred at 0°C for 10 min. Then, the acid chloride intermediate was added at 0°C, and the mixture was stirred at rt for 16 h, carefully quenched with saturated aq. NH_4_Cl and extracted with EtOAc (3 x 50 mL). The combined organic layer was dried over Na_2_SO_4_, filtered, concentrated under reduced pressure, and purified by combiflash reversed‐phase flash chromatography (C18) (0.1% TFA in water, 10% to 100% MeCN) to give compound **27** (43 mg, yield: 8%) as a colorless solid. ^1^H NMR (400 MHz, CD_3_OD) *δ* = 9.49 (s, 1H), 7.44 (dd, *J =* 7.2, 9.2 Hz, 1H), 7.07–7.05 (m, 3H) ppm. ^13^C NMR (126 MHz, DMSO‐*d*_6_) *δ* = 164.9, 161.0, 159.3, 157.5, 156.4, 144.6–142.4 (m), 143.4–141.1 (m), 129.9, 127.6, 122.2, 118.4 (t, *J =* 17.7 Hz), 115.8, 115.1, 114.9 (t, *J =* 14.9 Hz), 114.1, 54.9–53.9 (m) ppm. LCMS (ESI): *m/z* 414.1 ([M + H]^+^).

*Isoxazole‐3*,*4‐dicarboxylic* (***27a***)*.* To a solution of 3‐(*tert*‐butyl) 4‐ethyl isoxazole‐3,4‐dicarboxylate **27b** (0.50 g, 2.1 mmol) in aqueous HCl (10%, 10 mL) was added HOAc (3 mL), and the mixture was stirred at 70°C for 2 h, cooled to rt, and extracted with EtOAc (3 x 50 mL). The combined organic layer was dried over Na_2_SO_4_, filtered, concentrated under reduced pressure, and purified by reversed‐phase flash chromatography (C18) (0.1% TFA in water, 10% to 100% MeCN) to give intermediate **27a** (0.20 g, yield: 61%) as a colorless solid. LCMS (ESI): *m/z* 158.1 ([M + H]^+^).

*N‐*[*2*,*3*,*5*,*6‐Tetrafluoro‐3′‐*(^2^ *H*_3_)*‐methoxy‐*[*1*,*1′‐biphenyl*]*‐4‐yl*]*thiophene‐3*,*4‐dicarboxamide* (***28***)*.* To a solution of acid **20** (10 g, 23 mmol), DMAP (2.8 g, 23 mmol), and NH_4_Cl (12 g, 0.22 mol) in DMF (100 mL) was added EDC•HCl (8.9 g, 46 mmol). The mixture was stirred at 60°C overnight, cooled to rt, diluted with water (150 mL), and extracted with EtOAc (3 x 100 mL). The combined organic layer was dried over Na_2_SO_4_, filtered, concentrated under reduced pressure, and purified by flash chromatography (0% to 8% MeOH in CH_2_Cl_2_) to furnish an off‐colorless solid (9 g). MeCN (200 mL) was added to the solid and stirred at rt for 2 h. The mixture was filtered, the solid was washed with MeCN twice, and dried in vacuum at 60°C to afford compound **28** (5.0 g, yield: 50%) as a colorless solid. ^1^H NMR (400 MHz, DMSO‐*d*_6_) *δ* = 12.22 (s, 1H), 8.38 (d, *J =* 3.3 Hz, 2H), 8.26 (d, *J =* 3.3 Hz, 1H), 7.86 (s, 1H), 7.48 (t, *J =* 7.9 Hz, 1H), 7.23–7.02 (m, 3H) ppm. ^13^C NMR (126 MHz, DMSO‐*d*_6_) *δ* = 166.4, 160.8, 159.3, 144.6–142.3 (m), 143.2–140.9 (m), 135.2, 134.7, 134.5, 132.6, 129.9, 127.7, 122.2, 117.3 (t, *J* = 17.7 Hz), 116.4 (t, *J* = 14.9 Hz), 115.7, 114.9, 54.9–54.0 (m) ppm. LCMS (ESI): *m/z* 428.0 ([M + H]^+^).

*N* ^3^ *‐Methyl‐N* ^4^ *‐*[*2*,*3*,*5*,*6‐tetrafluoro‐3′‐*(^2^ *H*_3_)*‐methoxy‐*[*1*,*1′‐biphenyl*]*‐4‐yl*]*thiophene‐3*,*4‐dicarboxamide* (***29***)*.* Preparation according to GP2, using carboxylic acid **2** **0** (0.10 g, 0.23 mmol) and methylamine hydrochloride **29a** (47 mg, 0.69 mmol) to obtain compound **2** **9** (61 mg, yield: 59%) as a colorless powder after purification by flash column chromatography on silica gel (petroleum ether/EtOAc = 9:1). ^1^H NMR (300 MHz, DMSO‐*d*_6_) *δ* = 11.87 (s, 1H), 8.81 (d, *J =* 6.0 Hz, 1H), 8.39 (d, *J =* 6.0 Hz, 2H), 8.15 (d, *J =* 3.0 Hz, 1H), 7.50 (td, *J =* 6.0, 3.0 Hz, 1H), 7.16–7.10 (m, 3H), 2.82 (d, *J =* 5.7 Hz, 3H) ppm. ^13^C NMR (126 MHz, DMSO‐*d*_6_) *δ* = 164.8, 160.8, 159.3, 144.6–142.3 (m), 143.3–140.9 (m), 135.2, 134.6, 134.5, 131.3, 129.9, 127.7, 122.2, 117.4 (t, *J*  = 17.7 Hz), 116.3 (t, *J*  = 15.1 Hz), 115.8, 114.9, 54.7–54.1 (m), 26.3 ppm. LCMS (ESI): *m/z* 441.9 ([M + H]^+^).

*N* ^3^,*N* ^3^ *‐Dimethyl‐N* ^4^ *‐*[*2*,*3*,*5*,*6‐tetrafluoro‐3′‐*(^2^ *H*_3_)*‐methoxy‐*[*1*,*1′‐biphenyl*]*‐4‐yl*)*thiophene‐3*,*4‐dicarboxamide* (***30***). Preparation according to GP2, using carboxylic acid **20** (0.15 g, 0.42 mmol) and a 25% solution of dimethylamine in ethanol **30a** (0.3 mL) to obtain compound **30** (94 mg, yield: 89%) as a colorless powder after purification by flash column chromatography on silica gel (petroleum ether/EtOAc = 9:1). ^1^H NMR (300 MHz, DMSO‐*d*_6_) *δ* = 10.65 (s, 1H), 8.45 (d, *J =* 3.0 Hz, 1H), 7.71 (d, *J =* 3.0 Hz, 1H), 7.49 (td, *J =* 6.0, 3.0 Hz, 1H), 7.16–7.09 (m, 3H), 2.93 (s, 3H), 2.80 (s, 3H) ppm. ^13^ ^C^ NMR (126 MHz, DMSO‐*d*_6_) *δ* = 165.9, 160.6, 159.3, 144.5–142.3 (m), 143.6–141.4 (m), 137.9, 133.4, 131.3, 129.9, 127.7, 125.6, 122.2, 117.8 (t, *J* = 17.6 Hz), 116.2 (t, *J*  = 15.3 Hz), 115.8, 115.0, 54.9–54.1 (m), 38.0, 34.3 ppm. LCMS (ESI): *m/z* 455.9 ([M + H]^+^).

*N* ^3^ *‐Isopropyl‐N* ^4^ *‐*[*2*,*3*,*5*,*6‐tetrafluoro‐3′‐*(^2^ *H*_3_)*‐methoxy‐*[*1*,*1′‐biphenyl*]*‐4‐yl*]*thiophene‐3*,*4‐dicarboxamide* (***31***)*.* Preparation according to GP1, using acid chloride **20a** (0.20 g, 0.46 mmol) and isopropylamine **31a** (59 µL, 0.69 mmol) to obtain compound **31** (0.20 g, yield: 98%) as a colorless powder. ^1^H NMR (300 MHz, DMSO‐*d*_6_) *δ* = 11.73 (s, 1H), 8.69 (d, *J =* 7.6 Hz, 1H), 8.35 (d, *J =* 3.3 Hz, 1H), 8.12 (d, *J =* 3.3 Hz, 1H), 7.44 (td, *J =* 7.6, 0.6 Hz, 1H), 7.20–7.04 (m, 3H), 4.13–3.95 (m, 1H), 1.18 (s, 3H), 1.15 (s, 3H) ppm. ^13^C NMR (126 MHz, DMSO‐*d*_6_) *δ* = 163.5, 161.0, 159.3, 144.6–142.3 (m), 143.2–141.0 (m), 135.6, 134.4, 134.3, 131.3, 129.9, 127.7, 122.2, 117.5 (t, *J =* 17.7 Hz), 116.3 (t, *J =* 14.8 Hz), 115.8, 114.9, 54.8–54.0 (m), 41.4, 22.0 ppm. LCMS (ESI): *m/z* 470.0 ([M + H]^+^).

*N* ^3^ *‐Cyclopropyl‐N* ^4^ *‐*[*2*,*3*,*5*,*6‐tetrafluoro‐3′‐*(^2^ *H*_3_)*‐methoxy‐*[*1*,*1′‐biphenyl*]*‐4‐yl*]*thiophene‐3*,*4‐dicarboxamide* (***32***)*.* Preparation according to GP1, using acid chloride **20a** (0.20 g, 0.46 mmol) and cyclopropylamine **32a** (48 µL, 0.69 mmol) to obtain compound **32** (0.20 g, yield: 96%) as a colorless powder. ^1^H NMR (300 MHz, DMSO‐*d*_6_) *δ* = 11.68 (s, 1H), 8.81 (d, *J =* 4.2 Hz, 1H), 8.34 (d, *J =* 3.4 Hz, 1H), 8.09 (d, *J =* 3.4 Hz, 1H), 7.48 (dd, *J =* 9.7, 6.0 Hz, 1H), 7.16–7.05 (m, 3H), 2.85–2.79 (m, 1H), 0.74–0.63 (m, 4H) ppm. ^13^C NMR (126 MHz, DMSO‐*d*_6_) *δ* = 165.6, 160.9, 159.3, 144.6–142.3 (m), 143.2–141.0 (m), 135.4, 134.5, 134.2, 131.2, 129.9, 127.7, 122.3, 117.4 (t, *J =* 17.7 Hz), 116.3 (t, *J =* 15.2 Hz), 115.8, 114.9, 54.8–53.9 (m), 23.0, 5.7 ppm. LCMS (ESI): *m/z* 468.0 ([M + H]^+^).

*N* ^3^ *‐*(*Oxetan‐3‐yl*)*‐N* ^4^ *‐*[*2*,*3*,*5*,*6‐tetrafluoro‐3′‐*(^2^ *H*_3_)*‐methoxy‐*[*1*,*1′‐biphenyl*]*‐4‐yl*]*thiophene‐3*,*4‐dicarboxamide* (***33***)*.* Preparation according to GP1, using acid chloride **20a** (0.20 g, 0.46 mmol) and 3‐aminooxetane **33a** (48 µL, 0.69 mmol) to obtain compound **33** (19 mg, yield: 9%) as a colorless powder. ^1^H NMR (300 MHz, DMSO‐*d*_6_) *δ* = 11.30 (s, 1H), 9.39 (d, *J =* 6.4 Hz, 1H), 8.33 (d, *J =* 3.2 Hz, 1H), 8.15 (d, *J =* 3.2 Hz, 1H), 7.48 (dd, *J =* 9.7, 6.0 Hz, 1H), 7.17–7.04 (m, 3H), 5.00–4.88 (m, 1H), 4.77 (dd, *J =* 7.4, 6.2 Hz, 2H), 4.56 (t, *J =* 6.3 Hz, 2H) ppm. ^13^C NMR (126 MHz, DMSO‐*d*_6_) *δ* = 163.7, 161.0, 159.3, 144.5–142.3 (m), 143.3–141.0 (m), 135.7, 134.7, 133.4, 130.9, 129.9, 127.7, 122.2, 117.5 (t, *J =* 17.6 Hz), 116.3 (t, *J =* 15.3 Hz), 115.8, 114.9, 76.7, 55.0–54.0 (m), 44.6 ppm. LCMS (ESI): *m/z* 484.0 ([M + H]^+^).

*4‐*(*Azetidine‐1‐carbonyl*)*‐N‐*[*2*,*3*,*5*,*6‐tetrafluoro‐3′‐*(^2^ *H*_3_)*‐methoxy‐*[*1*,*1′‐biphenyl*]*‐4‐yl*]*thiophene‐3‐carboxamide* (***34***)*.* Preparation according to GP1, using acid chloride **20a** (0.30 g, 0.67 mmol) and azetidine hydrochloride **34a** (98 mg, 1.0 mmol) to obtain compound **34** (0.17 g, yield: 54%) as a colorless powder. ^1^H NMR (300 MHz, DMSO‐*d*_6_) *δ* = 11.25 (s, 1H), 8.37 (d, *J =* 3.1 Hz, 1H), 7.96 (d, *J =* 3.1 Hz, 1H), 7.53–7.41 (m, 1H), 7.17–7.05 (m, 3H), 4.14 (t, *J =* 7.6 Hz, 2H), 4.01 (t, *J =* 7.7 Hz, 2H), 2.23 (p, *J =* 7.8 Hz, 2H) ppm. ^13^C NMR (126 MHz, DMSO‐*d*_6_) *δ* = 165.4, 160.7, 159.3, 144.6–142.3 (m), 143.4–141.1 (m), 134.6, 133.6, 133.0, 129.9, 129.4, 127.7, 122.3, 117.6 (t, *J =* 17.6 Hz), 116.3 (t, *J =* 15.1 Hz), 115.8, 115.0, 55.0–54.0 (m), 51.7, 48.3, 15.1 ppm. LCMS (ESI): *m/z* 468.0 ([M + H]^+^).

*4‐*(*3*,*3‐Difluoroazetidine‐1‐carbonyl*)*‐N‐*[*2*,*3*,*5*,*6‐tetrafluoro‐3′‐*(^2^ *H*_3_)*‐methoxy‐*[*1*,*1′‐biphenyl*]*‐4‐yl*]*thiophene‐3‐carboxamide* (***35***)*.* Preparation according to GP1, using acid chloride **20a** (0.30 g, 0.67 mmol) and 3,3‐difluorazetidine hydrochloride **35a** (0.14 g, 1.0 mmol) to obtain compound **35** (0.14 g, yield: 41%) as a colorless powder. ^1^H NMR (300 MHz, DMSO‐*d*_6_) *δ* = 10.86 (s, 1H), 8.36 (d, *J =* 3.0 Hz, 1H), 8.03 (d, *J =* 3.0 Hz, 1H), 7.47 (dd, *J =* 8.3, 7.4 Hz, 1H), 7.23–7.05 (m, 3H), 4.48 (br s, 4H) ppm. ^13^C NMR (126 MHz, DMSO‐*d*_6_) *δ* = 165.9 (t, *J =* 3.6 Hz), 161.0, 159.3, 144.6–142.3 (m), 143.5–141.2 (m), 134.5, 133.9, 132.1, 129.9, 129.2, 127.7, 122.2, 117.8 (t, *J =* 17.6 Hz), 116.2 (t, *J =* 14.8 Hz), 116.0 (t, *J =* 272.1 Hz), 115.8, 115.0, 63.0–59.6 (m), 54.9–53.8 (m) ppm. LCMS (ESI): *m/z* 504.0 ([M + H]^+^).

*4‐*(*3‐Hydroxyazetidine‐1‐carbonyl*)*‐N‐*[*2*,*3*,*5*,*6‐tetrafluoro‐3′‐*(^2^ *H*_3_)*‐methoxy‐*[*1*,*1′‐biphenyl*]*‐4‐yl*]*thiophene‐3‐carboxamide* (***36***)*.* Preparation according to GP1, using acid chloride **20a** (0.30 g, 0.67 mmol) and azetidin‐3‐ol **36a** (77 mg, 1.0 mmol) to obtain compound **36** (0.18 g, yield: 55%) as a colorless powder. ^1^H NMR (300 MHz, DMSO‐*d*_6_) *δ* = 11.22 (s, 1H), 8.35 (d, *J =* 3.1 Hz, 1H), 7.97 (d, *J =* 3.1 Hz, 1H), 7.53–7.36 (m, 1H), 7.17–7.04 (m, 3H), 5.79 (d, *J =* 5.9 Hz, 1H), 4.58–4.42 (m, 1H), 4.32–4.07 (m, 2H), 3.95–3.91 (m, 1H), 3.77–3.72 (m, 1H) ppm. ^13^C NMR (126 MHz, DMSO‐*d*_6_) *δ* = 165.3, 160.7, 159.3, 144.6–142.3 (m), 143.4–141.1 (m), 134.6, 133.8, 132.9, 129.9, 129.6, 127.7, 122.3, 117.5 (t, *J =* 17.7 Hz), 116.3 (t, *J =* 15.1 Hz), 115.8, 115.0, 61.6, 60.0, 58.1, 54.9–53.9 (m) ppm. LCMS (ESI): *m/z* 484.0 ([M + H]^+^).

*N* ^3^ *‐*(*2‐Fluoroethyl*)*‐N* ^4^ *‐*[*2*,*3*,*5*,*6‐tetrafluoro‐3′‐*(^2^ *H*_3_)*‐methoxy‐*[*1*,*1′‐biphenyl*]*‐4‐yl*]*thiophene‐3*,*4‐dicarboxamide* (***37***)*.* Preparation according to GP1, using acid chloride **20a** (0.20 g, 0.45 mmol) and 2‐fluorethylamine hydrochloride **37a** (69 mg, 0.69 mmol) to obtain compound **37** (0.11 g, yield: 52%) as a colorless powder. ^1^H NMR (300 MHz, DMSO‐*d*_6_) *δ* = 11.50 (s, 1H), 9.05 (t, *J =* 5.6 Hz, 1H), 8.35 (d, *J =* 3.3 Hz, 1H), 8.15 (d, *J =* 3.2 Hz, 1H), 7.47 (td, *J =* 7.7, 0.6 Hz, 1H), 7.17–7.04 (m, 3H), 4.62 (t, *J =* 5.1 Hz, 1H), 4.46 (t, *J =* 5.0 Hz, 1H), 3.59 (q, *J =* 5.2 Hz, 1H), 3.51 (q, *J =* 5.2 Hz, 1H) ppm. ^13^C NMR (126 MHz, DMSO‐*d*_6_) *δ* = 164.4, 161.2, 159.3, 144.6–142.3 (m), 143.3–141.0 (m), 135.4, 134.5, 133.9, 131.4, 129.9, 127.7, 122.2, 117.5 (t, *J =* 17.7 Hz), 116.3 (t, *J =* 15.1 Hz), 115.8, 114.9, 82.0 (d, *J =* 165.7 Hz), 55.1–53.9 (m), 39.7 (d, *J =* 20.8 Hz) ppm. LCMS (ESI): *m/z* 474.0 ([M + H]^+^).

*N* ^3^ *‐*(*2*,*2‐Difluoroethyl*)*‐N* ^4^ *‐*[*2*,*3*,*5*,*6‐tetrafluoro‐3′‐*(^2^ *H*_3_)*‐methoxy‐*[*1*,*1′‐biphenyl*]*‐4‐yl*]*thiophene‐3*,*4‐dicarboxamide* (***38***)*.* Preparation according to GP1, using acid chloride **20a** (0.30 g, 0.67 mmol) and 2,2‐difluoroethan‐1‐amine hydrochloride **38a** (0.12 g, 1.0 mmol) to obtain compound **38** (0.19 g, yield: 58%) as a colorless powder. ^1^H NMR (300 MHz, DMSO‐*d*_6_) *δ* = 11.22 (s, 1H), 9.15 (t, *J =* 6.0 Hz, 1H), 8.33 (d, *J =* 3.2 Hz, 1H), 8.14 (d, *J =* 3.2 Hz, 1H), 7.46 (t, *J =* 7.9 Hz, 1H), 7.14–7.07 (m, 3H), 6.10 (tt, *J =* 57.0, 3.9 Hz, 1H), 4.13–3.16 (m, 2H) ppm. ^13^C NMR (126 MHz, DMSO‐*d*_6_) *δ* = 164.4, 161.5, 159.3, 144.6–142.3 (m), 143.3–141.1 (m), 135.4, 134.5, 133.4, 131.4, 129.9, 127.7, 122.3, 117.6 (t, *J =* 17.7 Hz), 116.3 (t, *J =* 15.5 Hz), 115.7, 115.0, 114.4 (t, *J =* 240.1 Hz), 55.1–54.1 (m), 41.5 (t, *J =* 26.1 Hz) ppm. LCMS (ESI): *m/z* 491.9 ([M + H]^+^).

*N* ^3^ *‐*[*2*,*3*,*5*,*6‐Tetrafluoro‐3′‐*(^2^ *H*_3_)*‐methoxy‐*[*1*,*1′‐biphenyl*]*‐4‐yl*]*‐N* ^4^ *‐*(*2*,*2*,*2‐trifluoroethyl*)*thiophene‐3*,*4‐dicarboxamide* (***39***)*.* Preparation according to GP1, using acid chloride **20a** (0.20 g, 0.45 mmol) and 2,2,2‐trifluorethylamine hydrochloride **39a** (94 mg, 0.69 mmol) to obtain compound **39** (0.12 g, yield: 53%) as a colorless powder. ^1^H NMR (300 MHz, DMSO‐*d*_6_) *δ* = 11.04 (s, 1H), 9.32 (t, *J =* 6.3 Hz, 1H), 8.30 (d, *J =* 3.2 Hz, 1H), 8.14 (d, *J =* 3.2 Hz, 1H), 7.47 (td, *J =* 7.7, 0.6 Hz, 1H), 7.14–7.08 (m, 3H), 4.12–4.00 (m, 2H) ppm. ^13^C NMR (126 MHz, DMSO‐*d*_6_) *δ* = 164.1, 161.6, 159.3, 144.5–142.3 (m), 143.4–141.1 (m), 135.4, 134.6, 132.8, 131.5, 129.9, 127.7, 124.7 (q, *J =* 279.4 Hz), 122.2, 117.7 (t, *J =* 17.7 Hz), 116.2 (t, *J =* 14.9 Hz), 115.8, 115.0, 54.9–54.0 (m), 40.3 (q, *J =* 33.4 Hz) ppm. LCMS (ESI): *m/z* 509.9 ([M + H]^+^).

*N* ^3^ *‐*(*Prop‐2‐yn‐1‐yl*)*‐N* ^4^ *‐*[*2*,*3*,*5*,*6‐tetrafluoro‐3′‐*(^2^ *H*_3_)*‐methoxy‐*[*1*,*1′‐biphenyl*]*‐4‐yl*]*thiophene‐3*,*4‐dicarboxamide* (***40***)*.* Preparation according to GP1, using acid chloride **20a** (0.30 g, 0.67 mmol) and 2‐propynylamine **40a** (67 µL, 1.0 mmol) to obtain compound **40** (61 mg, yield: 59%) as a colorless powder. ^1^H NMR (300 MHz, DMSO‐*d*_6_) ***δ*** = 11.41 (s, 1H), 9.23 (t, *J =* 5.5 Hz, 1H), 8.33 (d, *J =* 3.3 Hz, 1H), 8.15 (d, *J =* 3.2 Hz, 1H), 7.47 (t, *J =* 7.9 Hz, 1H), 7.17–7.04 (m, 3H), 4.05 (dd, *J =* 5.5, 2.6 Hz, 2H), 3.17 (t, *J =* 2.5 Hz, 1H) ppm. ^13^C NMR (126 MHz, DMSO‐*d*_6_) *δ* = 163.6, 161.3, 159.3, 144.6–142.3 (m), 143.3–141.0 (m), 135.1, 134.6, 133.7, 131.6, 129.9, 127.7, 122.2, 117.5 (t, *J =* 17.7 Hz), 116.3 (t, *J =* 15.1 Hz), 115.8, 114.9, 80.6, 73.4, 55.0–53.9 (m), 28.6 ppm. LCMS (ESI): *m/z* 466.0 ([M + H]^+^).

*N* ^3^ *‐*(*Cyanomethyl*)*‐N* ^4^ *‐*[*2*,*3*,*5*,*6‐tetrafluoro‐3′‐*(^2^ *H*_3_)*‐methoxy‐*[*1*,*1′‐biphenyl*]*‐4‐yl*]*thiophene‐3*,*4‐dicarboxamide* (***41***)*.* Preparation according to GP1, using acid chloride **20a** (0.30 g, 0.67 mmol) and aminoacetonitrile hydrochloride **41a** (96 mg, 1.0 mmol) to obtain compound **41** (0.31 g, yield: 99%) as a colorless powder. ^1^H NMR (300 MHz, DMSO‐*d*_6_) *δ* = 11.06 (s, 1H), 9.33 (t, *J =* 5.5 Hz, 1H), 8.30 (d, *J =* 3.1 Hz, 1H), 8.14 (d, *J =* 3.2 Hz, 1H), 7.47 (t, *J =* 7.9 Hz, 1H), 7.15–7.07 (m, 3H), 4.30 (d, *J =* 5.6 Hz, 2H) ppm. ^13^C NMR (126 MHz, DMSO‐*d*_6_) *δ* = 163.9, 161.5, 159.3, 144.6–142.3 (m), 143.4–141.1 (m), 135.1, 134.7, 132.8, 131.4, 129.9, 127.7, 122.3, 117.6 (t, *J =* 17.7 Hz), 117.4, 116.2 (t, *J =* 14.9 Hz), 115.8, 115.0, 55.1–53.9 (m), 27.6 ppm. LCMS (ESI): *m/z* 467.0 ([M + H]^+^).

*N* ^3^ *‐Cyanoethyl‐N* ^4^ *‐*[*2*,*3*,*5*,*6‐tetrafluoro‐3′‐*(^2^ *H*_3_)*‐methoxy‐*[*1*,*1′‐biphenyl*]*‐4‐yl*]*thiophene‐3*,*4‐dicarboxamide* (***42***)*.* Preparation according to GP1, using acid chloride **20a** (0.20 g, 0.45 mmol) and 3‐aminopropionitrile **42a** (stabilized with K_2_CO_3_) (50 µL, 0.69 mmol) to obtain compound **42** (0.17 g, yield: 79%) as a colorless powder. ^1^H NMR (300 MHz, DMSO‐*d*_6_) *δ* = 11.20 (br s, 1H), 9.15 (t, *J =* 5.8 Hz, 1H), 8.34 (d, *J =* 3.2 Hz, 1H), 8.12 (d, *J =* 3.2 Hz, 1H), 7.53–7.42 (m, 1H), 7.17–7.04 (m, 3H), 3.48 (q, *J =* 6.3 Hz, 2H), 2.76 (t, *J =* 6.5 Hz, 2H) ppm. ^13^C NMR (126 MHz, DMSO‐d_6_) *δ* = 164.4, 161.1, 159.3, 144.5–142.3 (m), 143.3–141.0 (m), 135.4, 134.6, 133.8, 131.2, 129.9, 127.7, 122.2, 119.2, 117.4 (t, *J =* 17.6 Hz), 116.4 (t, *J =* 15.1 Hz), 115.8, 114.9, 55.3–53.6 (m), 35.5, 17.3 ppm. LCMS (ESI): *m/z* 480.9 ([M + H]^+^).

*N* ^3^ *‐Cyano‐N* ^4^ *‐*[*2*,*3*,*5*,*6‐tetrafluoro‐3′‐*(^2^ *H*_3_)*‐methoxy‐*[*1*,*1′‐biphenyl*]*‐4‐yl*]*thiophene‐3*,*4‐dicarboxamide* (***43***)*.* To a solution of carboxylic acid **20** (0.20 g, 0.47 mmol) and cyanamide (39 mg, 0.93 mmol) in THF (1 mL) and NMP (1 mL) was added EDC•HCl (0.18 g, 0.93 mmol) in three portions. The mixture was stirred at rt for 4 h, diluted with water, and extracted with EtOAc (3 x 10 mL). The combined organic layer was dried over Na_2_SO_4_, filtered, concentrated under reduced pressure, and purified by combiflash reversed‐phase chromatography (C18) (0.1% TFA in water, 10% to 100% ACN) to afford compound **43** (51 mg, yield: 24%) as a colorless solid. ^1^H NMR (400 MHz, CD_3_OD) *δ* = 8.39 (d, *J =* 3.8 Hz, 1H), 8.30 (d, *J =* 3.8 Hz, 1H), 7.55–7.32 (m, 1H), 7.07–7.04 (m, 3H) ppm. ^13^C NMR (126 MHz, DMSO‐d_6_) *δ* = 173.1, 160.2, 159.3, 144.6–142.4 (m), 143.0–140.8 (m), 137.0, 136.5, 134.9, 134.7, 129.9, 127.9, 122.3, 119.6, 117.3 (t, *J =* 15.0 Hz), 116.7 (t, *J =* 17.7 Hz), 115.7, 114.9, 55.0–53.8 (m) ppm. LCMS (ESI): *m/z* 453.2 ([M + H]^+^).

*4*,*4‐Difluoro‐N‐*[*2*,*3*,*5*,*6‐tetrafluoro‐3′‐*(^2^ *H*_3_)*‐methoxy‐*[*1*,*1′‐biphenyl*]*‐4‐yl*]*cyclopent‐1‐ene‐1*,*2‐dicarboxamide* (***45***)*.* Preparation according to GP2, using the corresponding carboxylic acid **45a** (0.10 g, 0.22 mmol) and 25% aqueous ammonia (0.3 mL) to obtain compound **45** (86 mg, yield: 86%) as a colorless powder after purification by flash column chromatography on silica gel (petroleum ether/EtOAc = 9:1). ^1^H NMR (300 MHz, DMSO‐*d*_6_) *δ* = 12.23 (s, 1H), 8.10 (s, 1H), 7.99 (s, 1H), 7.46 (t, *J =* 6.0 Hz, 1H), 7.12–7.06 (m, 3H), 3.52–3.33 (m, 4H) ppm. ^13^C NMR (75 MHz, DMSO‐*d*_6_) *δ* = 165.6, 161.1, 159.3, 136.8, 136.4, 129.9, 127.9, 127.7, 122.2, 117.5, 115.7, 114.9, 44.9, 44.7, 44.4 ppm. LCMS (ESI): *m/z* 447.9 ([M + H]^+^).

*N‐*[*3‐Fluoro‐3′‐*(*prop‐2‐yn‐1‐yloxy*)*‐*[*1*,*1′‐biphenyl*]*‐4‐yl*]*thiophene‐3*,*4‐dicarboxamide* (***47***). To a solution of carboxylic acid **47a** (0.10 g, 0.25 mmol) and HATU (0.14 g, 0.37 mmol) in DMF (4 mL) were added NH_4_Cl (81 mg, 1.5 mmol) and Hünig's base (0.19 g, 1.5 mmol). The mixture was stirred at rt overnight, diluted with water, and extracted with EtOAc (3 x 10 mL). The combined organic layer was dried over Na_2_SO_4_, filtered, concentrated under reduced pressure, and purified by reversed‐phase flash chromatography (C18) (0.1% NH_4_HCO_3_ in water, 10% to 100% MeCN) to afford compound **47** (50 mg, yield: 50%) as a colorless solid. ^1^H NMR (500 MHz, CD_3_OD) ***δ*** = 8.33 (d, *J =* 3.0 Hz, 1H), 8.22 (t, *J =* 8.0 Hz, 1H), 8.16 (d, *J =* 3.5 Hz, 1H), 7.48–7.45 (m, 2H), 7.37 (t, *J =* 7.8 Hz, 1H), 7.26–7.23 (m, 2H), 7.00–6.97 (m, 1H), 4.80 (d, *J =* 2.0 Hz, 2H), 2.97 (t, *J =* 2.3 Hz, 1H) ppm. ^13^C NMR (126 MHz, DMSO‐*d*_6_) *δ* = 166.9, 160.7, 157.7, 153.5 (d, *J =* 245.2 Hz), 139.9 (d, *J =* 1.7 Hz), 136.5 (d, *J =* 7.4 Hz), 136.2, 135.7, 133.8, 132.6, 130.0, 126.1 (d, *J =* 11.4 Hz), 123.4, 122.5 (d, *J =* 2.9 Hz), 119.5, 114.4, 113.5 (d, *J =* 20.3 Hz), 112.8, 79.3, 78.3, 55.5 ppm. LCMS (ESI): *m/z* 395.1 ([M + H]^+^).

*4‐{*[*3‐Fluoro‐3′‐*(*prop‐2‐yn‐1‐yloxy*)*‐*[*1*,*1′‐biphenyl*]*‐4‐yl*]*carbamoyl}thiophene‐3‐carboxylic acid* (***47a***). A solution of thiophene‐3,4‐dicarboxylic anhydride **47b** (0.39 g, 2.4 mmol) and aniline **47c** (0.49 g, 2.0 mmol) in MeCN (10 mL) was heated at 60°C for 2 h, cooled to rt, and filtered. The filter cake was washed with MeCN (2 x 20 mL) and dried in vacuum at 50°C to afford compound **47a** (0.63 g, yield: 78%) as an off‐colorless solid. LCMS (ESI): *m/z* 395.9 ([M + H]^+^).

*3‐Fluoro‐3′‐*(*prop‐2‐yn‐1‐yloxy*)*‐*[*1*,*1′‐biphenyl*]*‐4‐amine* (***47c***)*.* A solution of compound **4** **7d** (2.0 g, 9.8 mmol), 3‐bromoprop‐1‐yne (1.2 g, 10 mmol), and K_2_CO_3_ (4.1 g, 30 mmol) in DMF (20 mL) was stirred at rt for 6 h, then concentrated under reduced pressure and purified by flash chromatography (0% to 25% EtOAc in petroleum ether) to give compound 47c (1.2 g, yield: 50%) as a yellow oil. LCMS (ESI): *m/z* 242.1 ([M + H]^+^) ppm.

*4′‐Amino‐3′‐fluoro‐*[*1*,*1′‐biphenyl*]*‐3‐ol* (***47d*** **)** ***.*** To a solution of 2‐fluoro‐4‐(4,4,5,5‐tetramethyl‐1,3,2‐dioxaborolan‐2‐yl)aniline **4** **7f** (5.0 g, 21 mmol) and 3‐bromophenol 47e (3.6 g, 21 mmol) in 1,4‐dioxane (50 mL) and H_2_O (5 mL) were added Cs_2_CO_3_ (22 g, 68 mmol) and Pd(dppf)Cl_2_ (0.14 g, 1.9 mmol). The mixture was heated at 90°C for 16 h, cooled, filtered through a pad of Celite, concentrated under reduced pressure, and purified by flash chromatography (0% to 30% EtOAc in petroleum ether) to give compound **47d** (3.5 g, yield: 83%) as an off‐colorless solid. LCMS (ESI): *m/z* 204.1 ([M + H]^+^).

### 5.2 In Vitro Characterization

*Hybrid reporter gene assays*. Nuclear receptor modulation was determined in Gal4 hybrid reporter gene assays in HEK293T cells (German Collection of Microorganisms and Cell Culture GmbH, DSMZ; RRID:CVCL_0063) using pFR‐Luc (Stratagene, La Jolla, CA, USA; reporter), pRL‐SV40 (Promega, Madison, WI, USA; internal control), and pFA‐CMV‐hNR‐LBD [43, 44] plasmids coding for the hinge region and ligand‐binding domain of the canonical isoform of the respective NR. Cells were cultured in Dulbecco's modified Eagle's medium (DMEM), high glucose, supplemented with 10% fetal calf serum (FCS), sodium pyruvate (1 mM), penicillin (100 U/mL), and streptomycin (100 μg/mL) at 37°C and 5% CO_2_, and seeded in 96‐well plates (3 × 10^4^ cells/well). After 24 h, medium was changed to Opti‐MEM without supplements, and cells were transiently transfected using Lipofectamine LTX reagent (Invitrogen, Carlsbad, CA, USA) according to the manufacturer's protocol. Five hours after transfection, cells were incubated with the test compounds in Opti‐MEM supplemented with penicillin (100 U/mL), streptomycin (100 μg/mL), and 0.1% DMSO for 16 h before luciferase activity was measured using the Dual‐Glo Luciferase Assay System (Promega) according to the manufacturer's protocol on a Tecan Spark luminometer (Tecan Deutschland GmbH, Crailsheim, Germany). Firefly luminescence was divided by Renilla luminescence and multiplied by 1000, resulting in relative light units (RLU), to normalize for transfection efficiency and cell growth. Fold activation was obtained by dividing the mean RLU of the test compound by the mean RLU of the untreated control, and relative activation was calculated by dividing the fold activation of a test sample by the fold activation of the respective reference agonist. All samples were tested in at least three biologically independent experiments in duplicates. For dose–response curve fitting and calculation of EC_50_ values, the equation “[Agonist] vs. response – Variable slope (four parameters)” was used in GraphPad Prism (version 7.00, GraphPad Software, La Jolla, CA, USA).

*Reporter gene assays for full‐length human Nurr1.* Activation of full‐length human Nurr1 was studied in transiently transfected HEK293T cells using the reporter plasmids pFR‐Luc‐NBRE, pFR‐LUC‐POMC, or pFR‐Luc‐DR5, each containing one copy of the respective human Nurr1 response element NBRE Nl3, NurRE, or DR5 [34]. The full‐length human nuclear receptor Nurr1 (pcDNA3.1‐hNurr1‐NE; Addgene plasmid #102363) and, for DR5, RXRα (pSG5‐hRXR) were overexpressed. pRL‐SV40 (Promega) was used for normalization of transfection efficacy and to observe test compound toxicity. Cells were cultured in Dulbecco's modified Eagle's medium (DMEM), high glucose, supplemented with 10% fetal calf serum (FCS), sodium pyruvate (1 mM), penicillin (100 U/mL), and streptomycin (100 μg/mL) at 37°C and 5% CO_2_ and seeded in 96‐well plates (3 × 10^4^ cells/well). After 24 h, medium was changed to Opti‐MEM without supplements, and cells were transiently transfected using Lipofectamine LTX reagent (Invitrogen) according to the manufacturer's protocol. Five hours after transfection, cells were incubated with the test compounds in Opti‐MEM supplemented with penicillin (100 U/mL), streptomycin (100 μg/mL), and 0.1% DMSO for 16 h before luciferase activity was measured using the Dual‐Glo Luciferase Assay System (Promega) according to the manufacturer's protocol on a Tecan Spark luminometer (Tecan Deutschland GmbH, Germany). Firefly luminescence was divided by Renilla luminescence and multiplied by 1000, resulting in relative light units (RLU) to normalize for transfection efficiency and cell growth. Fold activation was obtained by dividing the mean RLU of test compound by the mean RLU of the untreated control. All samples were tested in at least three biologically independent experiments in duplicates. For dose–response curve fitting and calculation of EC_50_ values, the equation “[Agonist] versus response (three parameters)” was used in GraphPad Prism (version 7.00, GraphPad Software, La Jolla, CA, USA).

*DHODH inhibition assay*. Inhibition of DHODH was measured in vitro using an *N‐*terminally truncated recombinant DHODH enzyme similar to that described previously [35]. The final assay mixture contained 60 μM 2,6‐dichloroindophenol, 50 μM decylubiquinone, 100 μM dihydroorotate, and the DHODH protein, whose concentration was adjusted in a way that an average slope of ≈ 0.2 AU/min served as the positive control (no inhibitor). Measurements were performed in 50 mM Tris•HCl, 150 mM KCl, and 1% (v/v) Triton X‐100 at pH 8.0 and at 30°C, with at least six different concentrations of a test compound. The reaction was started by adding dihydroorotate and measuring the absorption at 600 nm for 2 min. Each test compound concentration used for IC_50_ calculation was tested in at least three independent experiments (except for the unstable compound **25**). IC_50_ was calculated with GraphPad Prism by normalizing the data and using a variable‐slope four‐parameter fit with top (DMSO control) and bottom (positive control) constraints.

*Isothermal titration calorimetry* (*ITC*). ITC experiments were conducted on an Affinity ITC instrument (TA Instruments, New Castle, DE, USA) at 25°C with a stirring rate of 75 rpm. Nurr1 LBD protein (15 μM, expressed as described previously [45]) in buffer (20 mM Tris, pH 7.5, 100 mM NaCl, 5% glycerol) containing 4% DMSO was titrated with **28** (80 μM in the same buffer containing 4% DMSO) in 21 injections (1 × 1 µL and 20 × 4 μL) with an injection interval of 120 s. As control experiments, the test compound was titrated to the buffer, and the buffer was titrated to the Nurr1 LBD protein under otherwise identical conditions. The heats of the compound−protein titration were analyzed using NanoAnalyze software (version 3.11.0, TA Instruments) with an independent binding model.

*Multiplex toxicity assay.* HEK293T cells were grown in DMEM high glucose, supplemented with 10% FCS, sodium pyruvate (1 × 10^−3 ^M), penicillin (100 U/mL), and streptomycin (100 μg/mL) at 37°C and 5% CO_2_ and seeded in 96‐well plates (2 × 10^4^ cells per well) in culture medium with reduced serum content (0.2%). The next day, low‐serum medium was refreshed and additionally contained 0.1% DMSO with **28** (1, 3, 10, or 30 µM), 0.1% DMSO with bexarotene (100 µM) as positive control, or 0.1% DMSO alone as untreated control. Each sample was prepared in 4 biologically independent replicates. After incubation for 24 h, the medium was changed to 90 μL culture medium without phenol red (0.2% FCS) and 10 μL Cell Counting Kit‐8 solution (CCK‐8, MedChem Express #HY‐K0301), and absorbance was measured after 2 h incubation at 450 nm on a Tecan Spark Cyto (Tecan Group AG) to assess the metabolic activity of the cells. Thereafter, Hoechst33342 (10 μM, #ab228551, Abcam Limited, Cambridge, UK), and Live‐or‐Dye Nuc‐Fix Red (0.05×, Biotium, Inc., Fremont, CA, 1691 USA) were added and incubated for 30 min to detect necrosis. After incubation, a total of 3 fluorescence images per well at 10× magnification were taken to detect Hoechst33342‐positive cell nuclei (Ex: 381−400 nm, Em: 414−450 nm) and Live‐or‐Dye positive cells (Ex: 543−566 nm, Em: 580−611 nm), respectively, using a Tecan Spark Cyto (Tecan Group AG). Necrotic cells were counted using CellProfiler (Version 4.2.6). Reference readings for background correction and detection of autofluorescence were taken at the same wavelengths prior to staining. Before drug administration, after the first medium exchange, 24 h after drug administration, and after fluorescence imaging, cell confluence was assessed using the Tecan Spark Cyto, to account for changes in cell confluence due to drug administration and cell handling. Data were normalized to the untreated (DMSO) control for each biological replicate.

### 5.3 In Vivo PK Study

PK and brain exposure of **28**, **45**, and **47** were evaluated in 3 female Sprague Dawley rats (8‐week‐old from Janvier, France) after oral cassette dosing. Rats were housed in a temperature‐controlled room (20°C–24°C) and maintained in a 12 h light/12 h dark cycle. Food (ssniff R/M‐H, 10 mm) and water were provided *ad libitum* before and during the study. All experimental procedures were approved by and conducted in accordance with the regulations of the local Animal Welfare authorities (Landesamt für Gesundheit und Verbraucherschutz, Abteilung Lebensmittel‐ und Veterinärwesen, Saarbrücken). The compounds were dosed at 20 mg/kg with an application volume of 5 mL/kg. 5% Solutol/95% NaCl solution (at 0.9% saline concentration) was used as a vehicle, yielding a milky suspension. Animals showed normal behavior, and no clinical signs were observed after dosing. 2 h post dose, the animals were sacrificed, blood samples (about 500 μL) were collected from the retrobulbar venous plexus, and stored at ‐20°C until analysis. Animals were then perfused with phosphate‐buffered saline (PBS) until PBS was transparent, and the brain was collected and stored at ‐20°C until analysis. Blood and brain concentrations were quantified by LCMS analysis.

### 5.4 Statistical Evaluation

In vitro assays were conducted with at least three independent repeats, which were increased when necessary to improve R^2^ for dose–response curves. Compounds/samples were considered active when *p *< 0.05 (two‐sided *t*‐test). Fitting of dose–response curves and statistical evaluation were performed in GraphPad Prism (version 7, GraphPad software).

## Funding

European Research Council (101040355).

## Conflicts of Interest

Christian Gege, Hella Kohlhof, and Daniel Vitt are inventors of WO2024/200872 claiming some compounds described in this study.

## Supporting information

Supporting Information (pdf) containing Supplementary Figures as well as NMR spectra, MS spectra and LCMS data of test compounds. Molecular formula strings (csv) containing chemical structures and activity data of 5–47.
