# Supplementary material for: Development of a Brain‐Penetrant Nurr1 Agonist Tool
Source: ChemMedChem. 2026 Jun 14;21(11):e70296. doi: 10.1002/cmdc.70296 (PMC13265399; doi:10.1002/cmdc.70296)
Supplement: Supplementary file 1 — Supporting Information (pdf) containing Supplementary Figures as well as NMR spectra, MS spectra and LCMS data of test compounds. Molecular formula strings (csv) containing chemical structures and activity data of 5–47. [file CMDC-21-e70296-s001.pdf]

- Supporting Information -

Development of a Brain-Penetrant Nurr1 Agonist Tool

Jan Vietor[1#], Tanja Stiller[1#], Christian Gege[2], Wael Saeb[3], Úrsula López-García[1], Hella Kohlhof[2], Daniel Vitt[2], and Daniel Merk[1*]

[1] Ludwig-Maximilians-Universität (LMU) München, Department of Pharmacy, 81377 Munich, Germany

[2] Immunic AG, 82166 Gräfelfing, Germany

[3] RebisLab R&D GmbH, 82152 Planegg-Martinsried, Germany

[#] J.V. and T.S. contributed equally to this study

* daniel.merk@cup.lmu.de

Table of Contents

Supplementary Figures S2

NMR spectra, MS spectra and LCMS data S3

Supplementary Figures

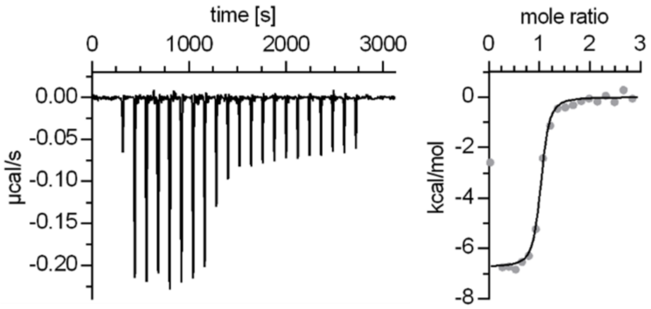

Figure S1. Isothermal titration calorimetry (ITC) confirmed binding of **28** to the recombinant Nurr1 ligand binding domain (K_d < 0.1 µM). The isotherm at 25 °C is shown on the left and the fitting of the heat of binding is shown on the right.

NMR spectra, MS spectra and LCMS data of **5-13**, **15**, **17**, **22**, **24-43**, **45** and **47**

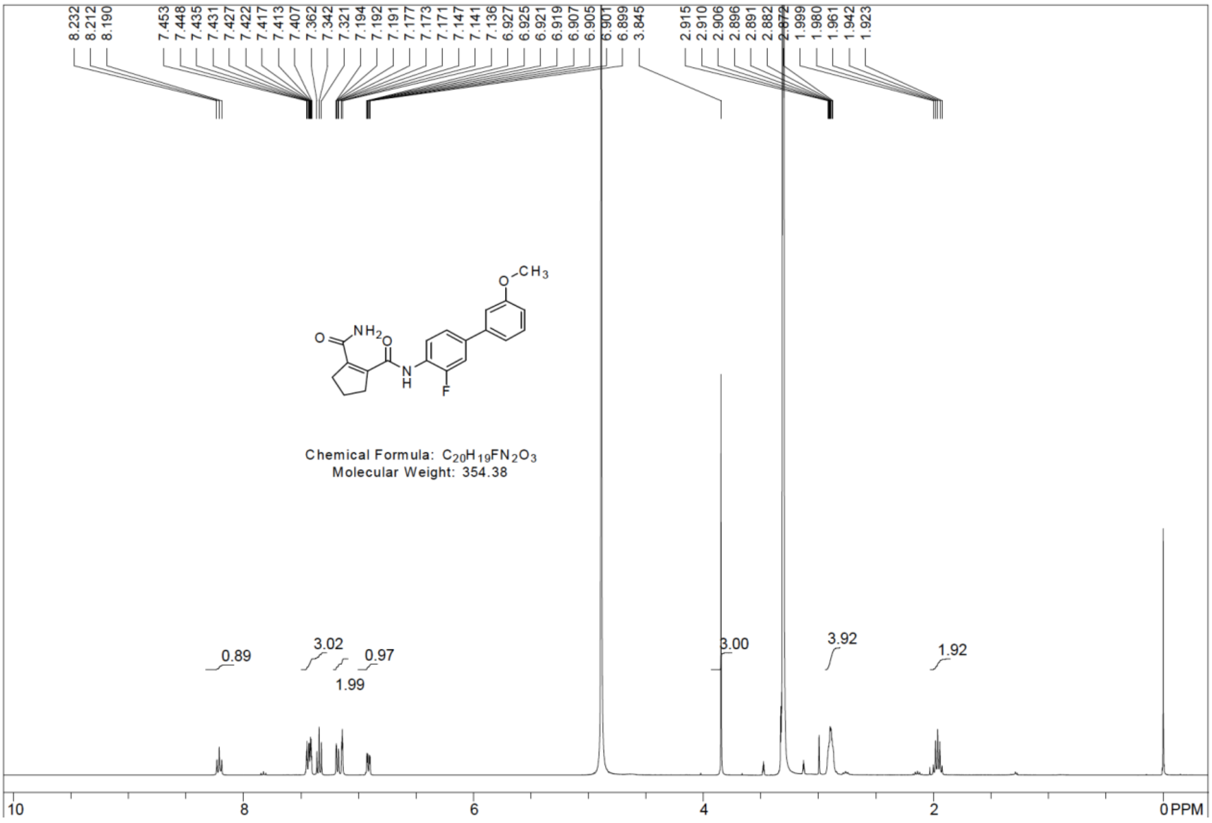

^{1}H-NMR (400 MHz, CD_3OD) of compound **5**

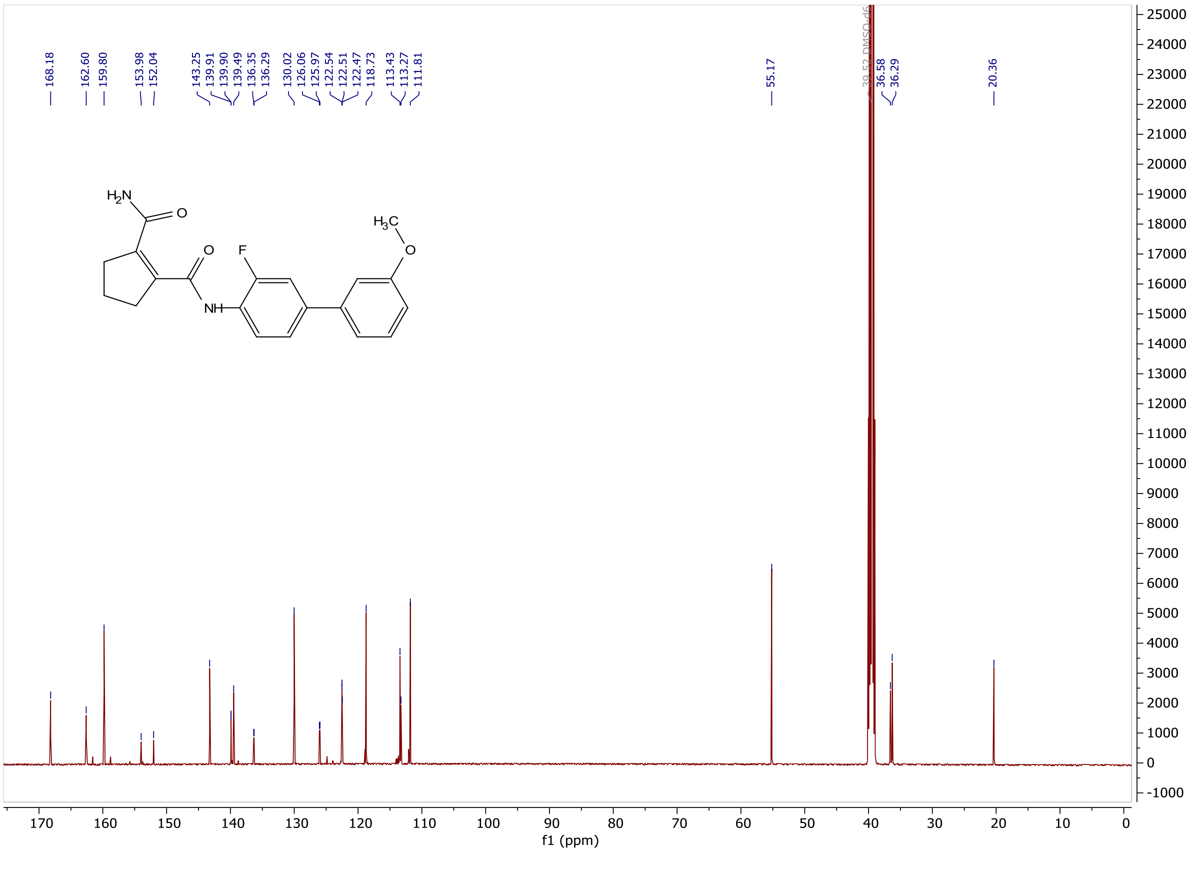

^{13}C-NMR (126 MHz, DMSO-d_6) of compound **5**

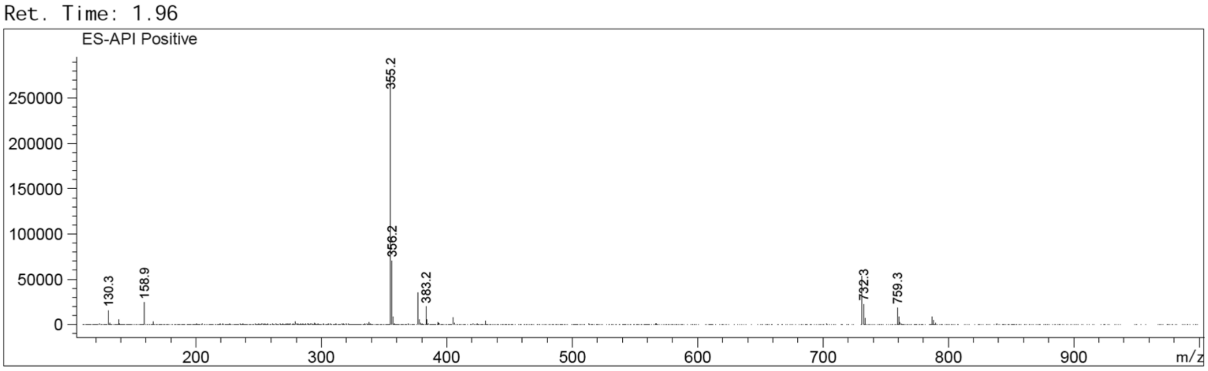

MS of compound **5**

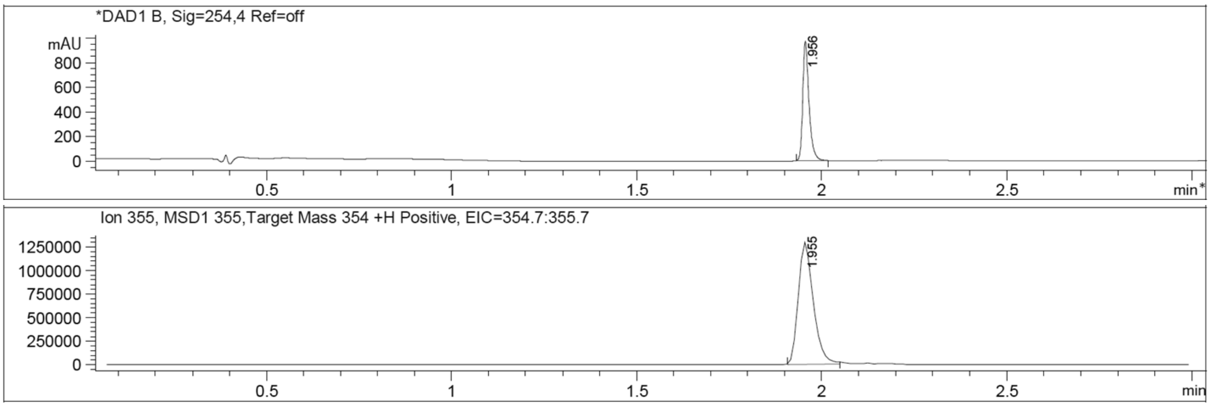

Chromatographic purity analysis of compound **5**

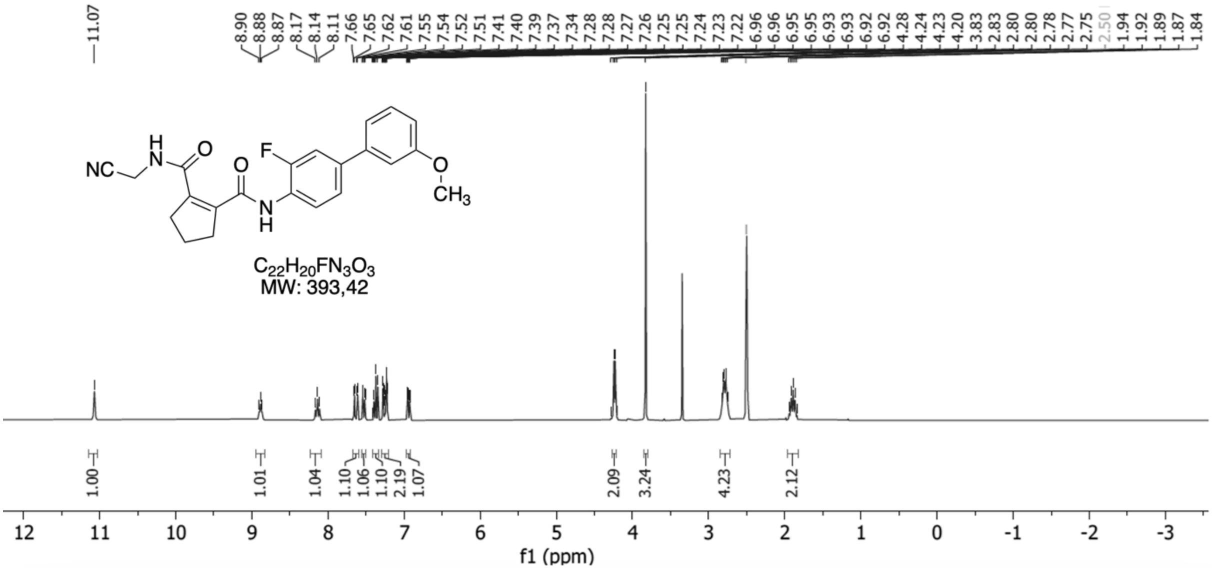

^{1}H-NMR (300 MHz, DMSO-d_6) of compound **6**

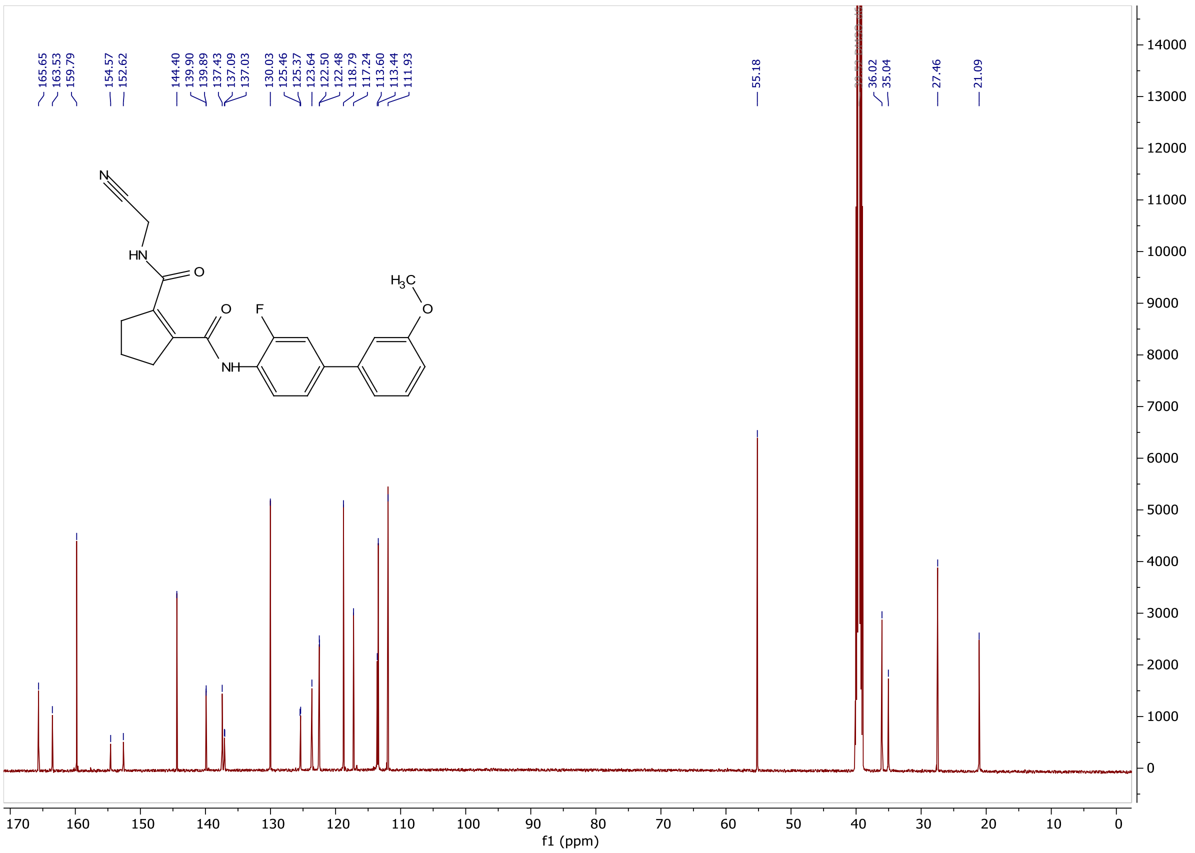

^{13}C-NMR (126 MHz, DMSO-d_6) of compound **6**

Peak ID	Compound	Time	Mass Found
7	Found	4.19	416.10,394.10

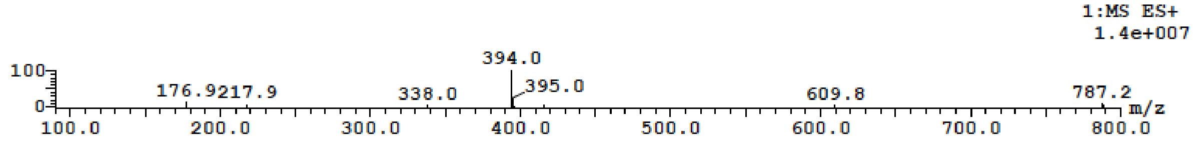

MS of compound **6**

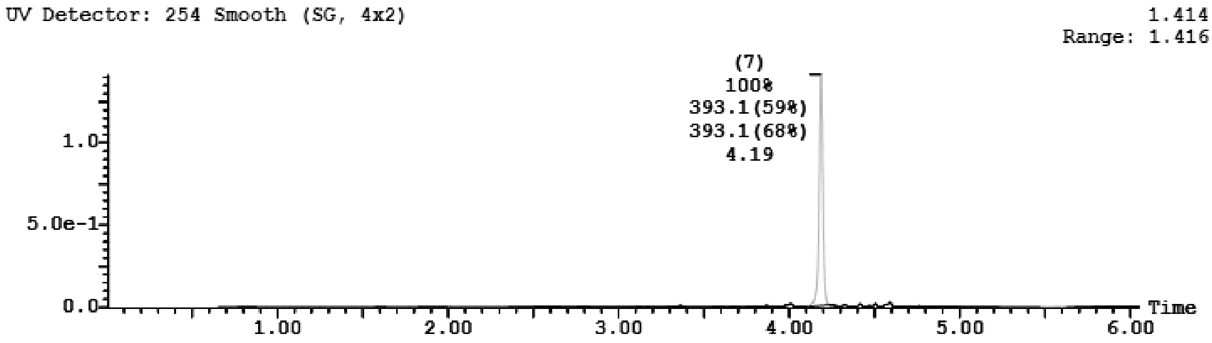

Peak Number	Compound	Time	AreaAbs	Area %Total	Width	Height	Mass Found
7	Found	4.19	4e+004	100.00	0	1e+006	393.10, 393.10

Chromatographic purity analysis of compound **6**

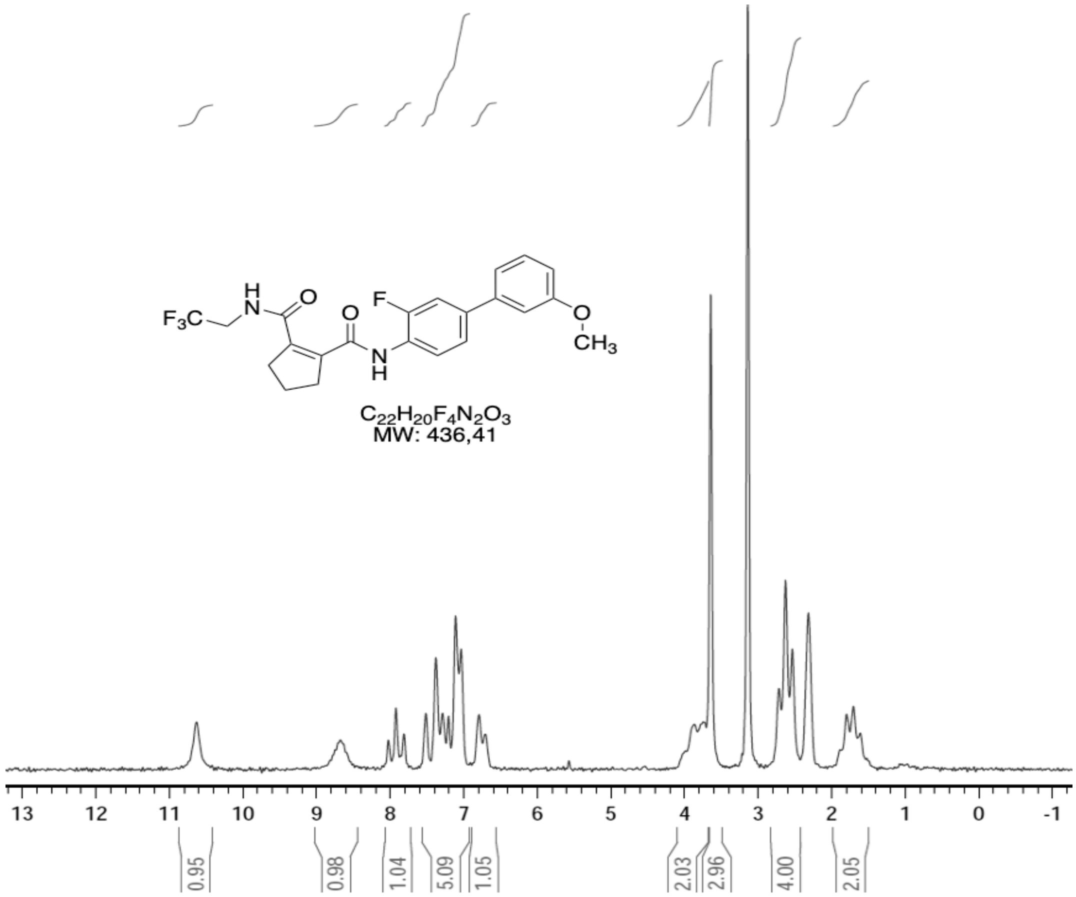

^{1}H-NMR (80 MHz, DMSO-d_6) of compound **7**

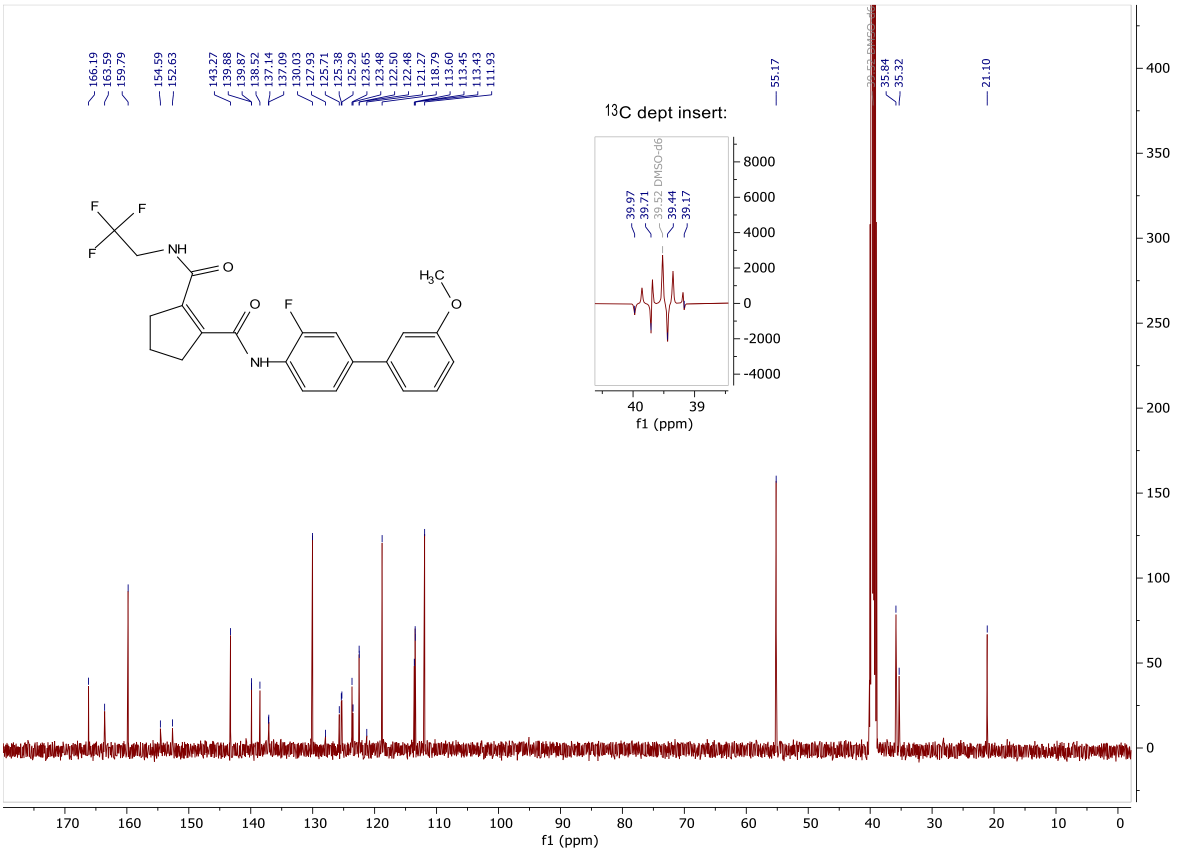

^{13}C-NMR (126 MHz, DMSO-d_6) of compound **7**

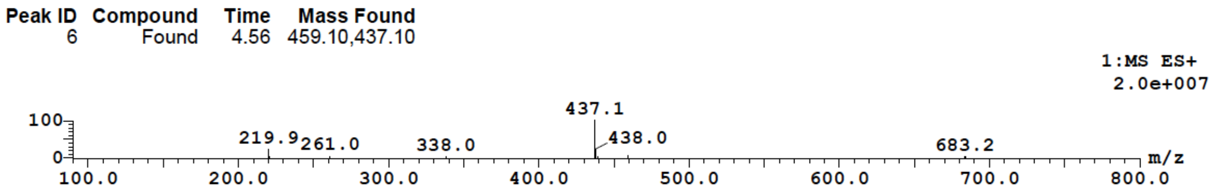

MS of compound **7**

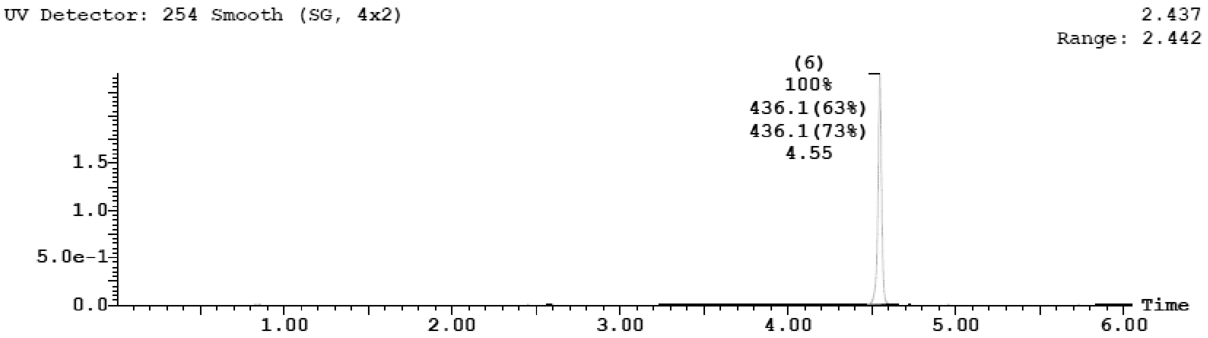

Chromatographic purity analysis of compound **7**

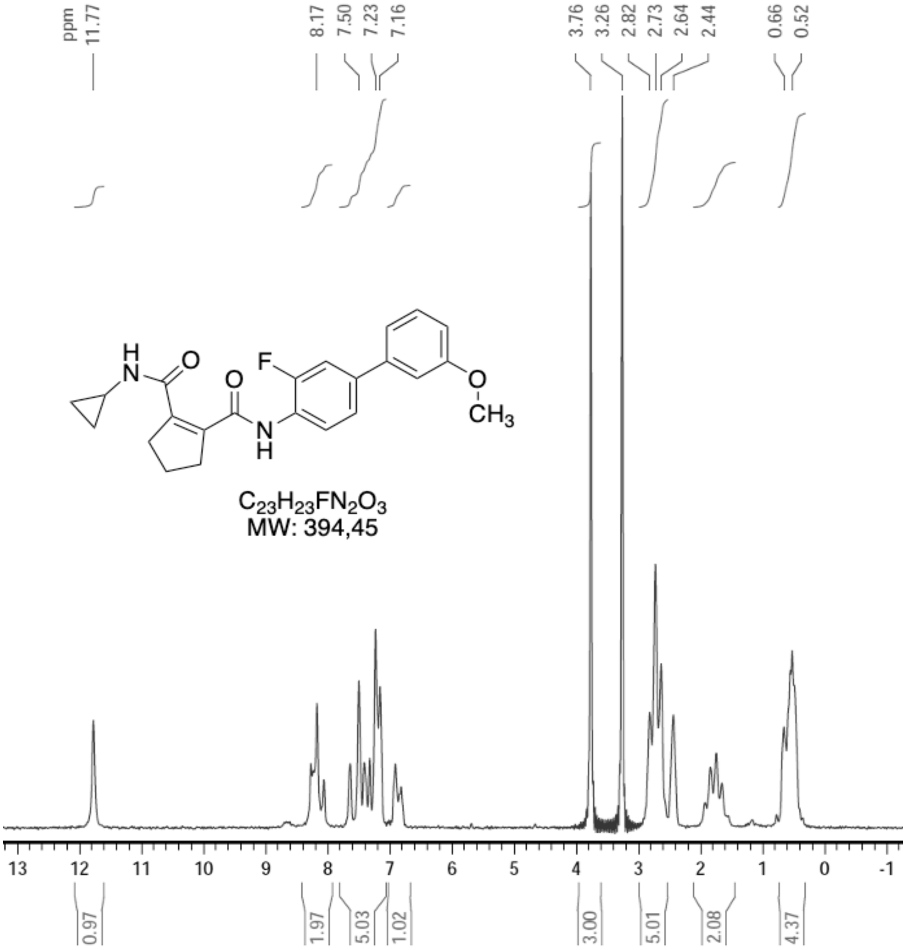

^{1}H-NMR (80 MHz, DMSO-d_6) of compound **8**

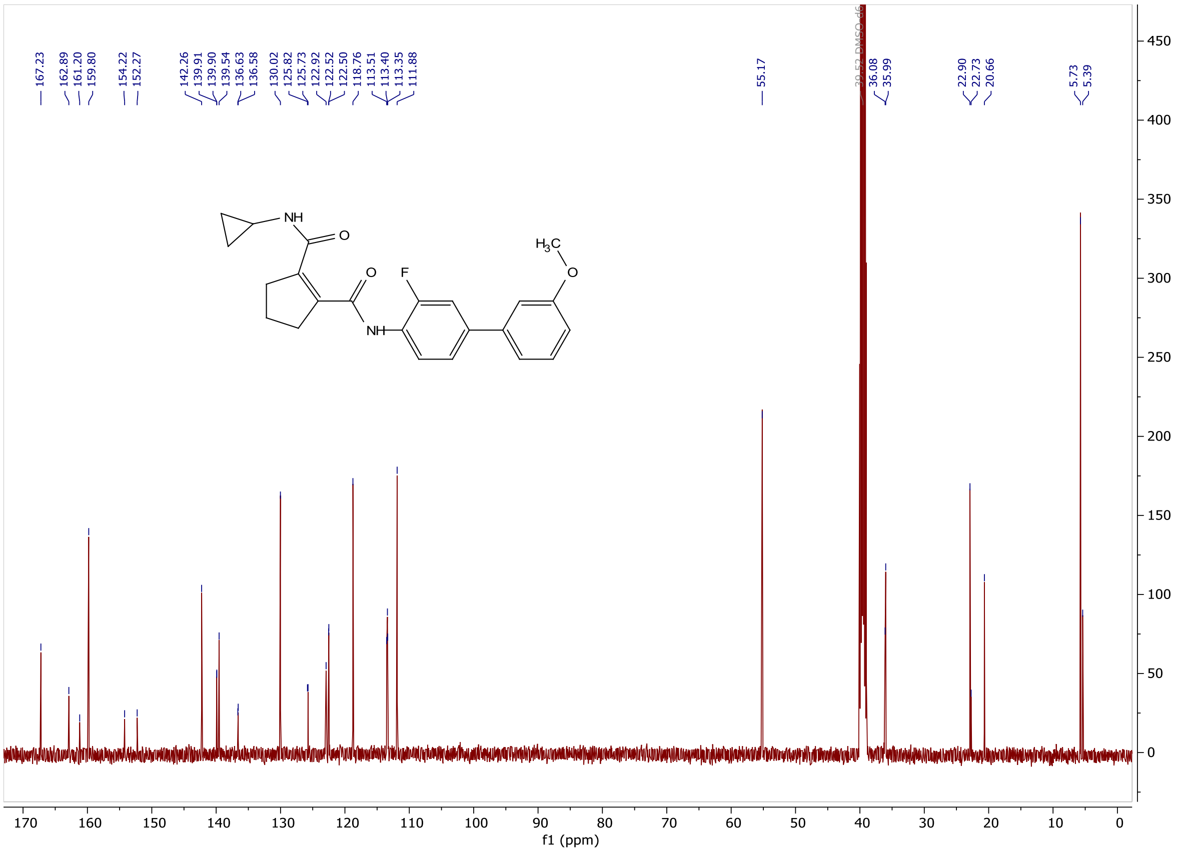

^{13}C-NMR (126 MHz, DMSO-d_6) of compound **8**

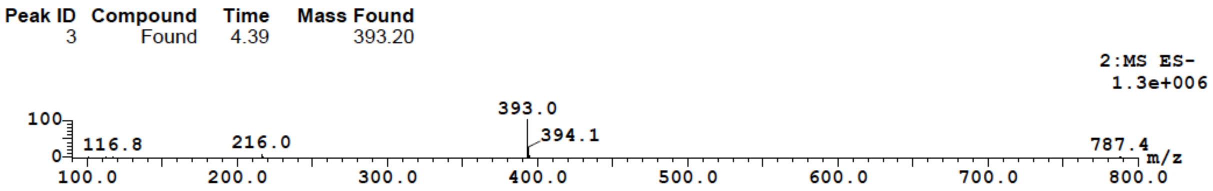

MS of compound **8**

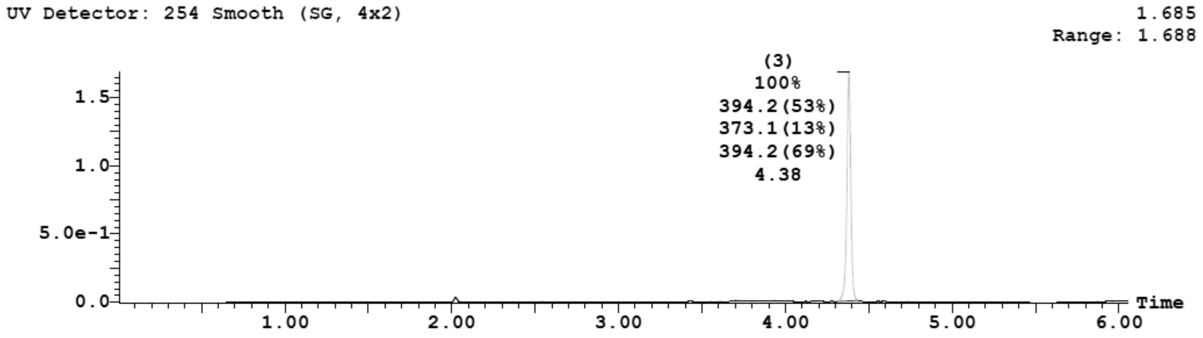

Chromatographic purity analysis of compound **8**

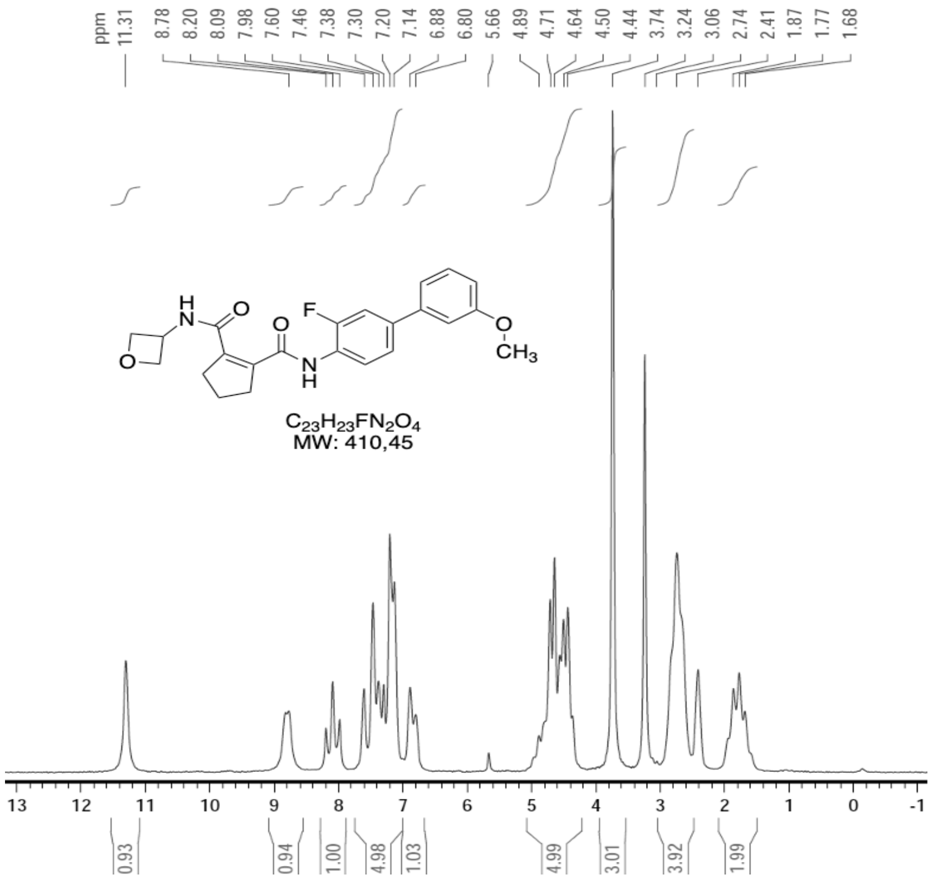

^{1}H-NMR (80 MHz, DMSO-d_6) of compound **9**

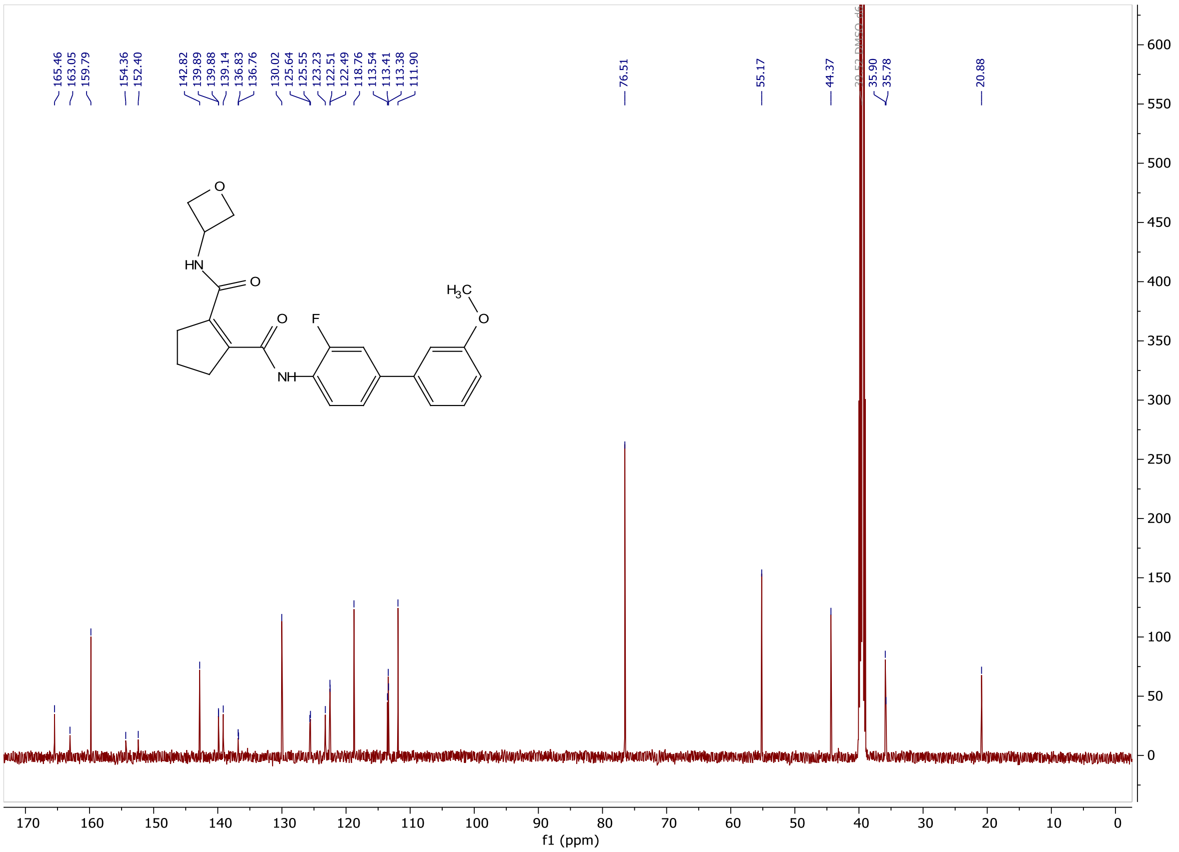

^{13}C-NMR (126 MHz, DMSO-d_6) of compound **9**

Peak ID	Compound	Time	Mass Found
8	Found	4.09	433.20,411.20

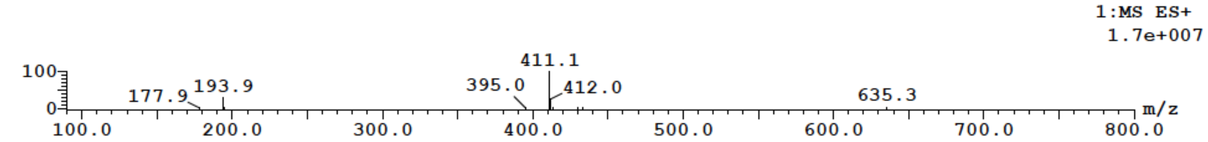

MS of compound **9**

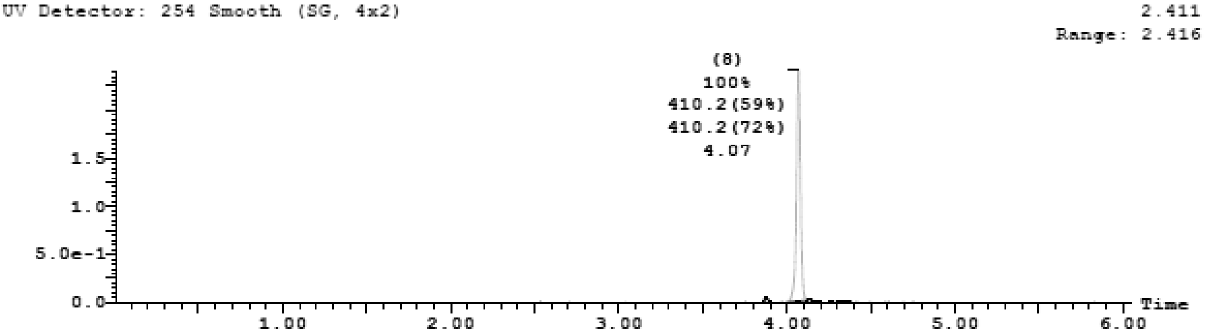

ak Number	Compound	Time	AreaAbs	Area %Total	Width	Height	Mass Found
8	Found	4.07	7e+004	100.00	0	2e+006	410.20, 410.20

Chromatographic purity analysis of compound **9**

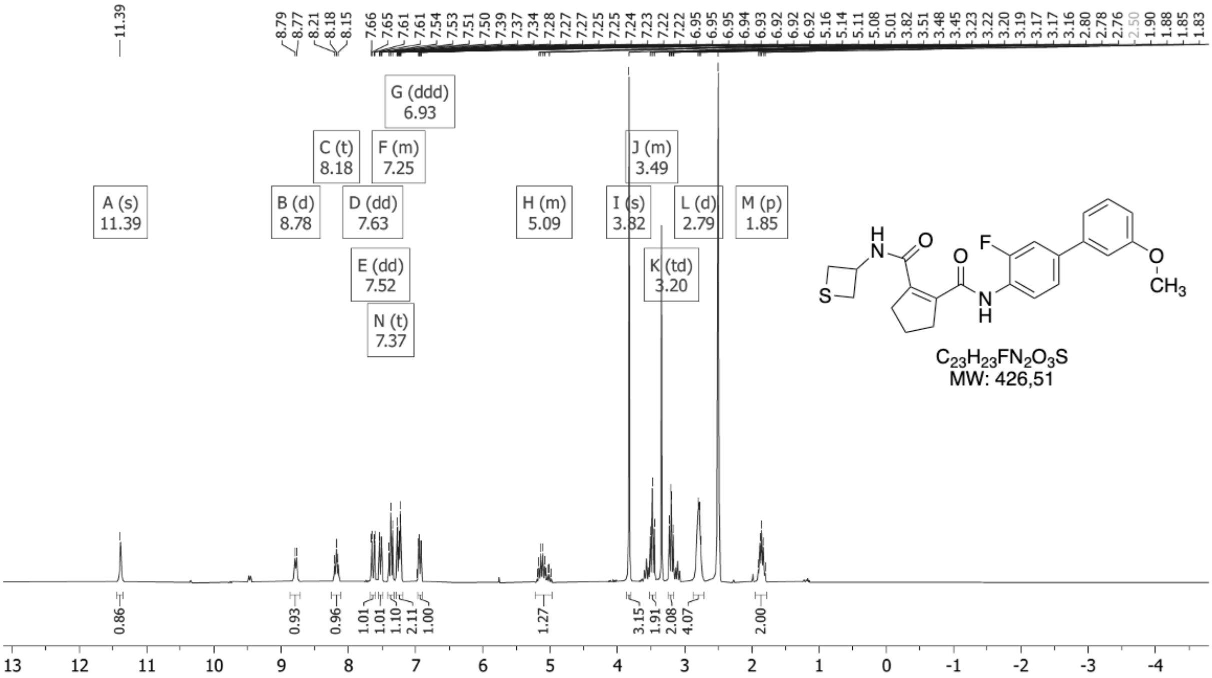

^{1}H-NMR (300 MHz, DMSO-d_6) of compound **10**

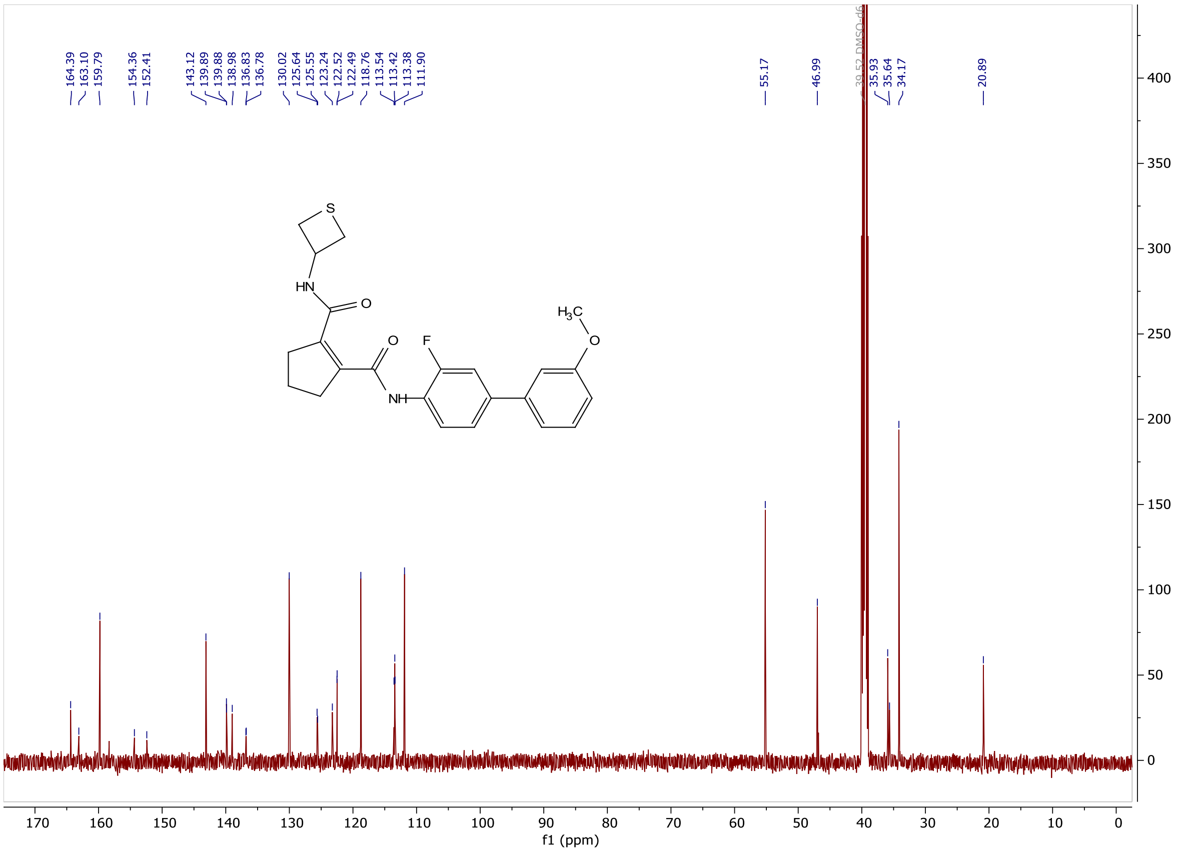

^{13}C-NMR (126 MHz, DMSO-d_6) of compound **10**

Peak ID	Compound	Time	Mass Found
9	Found	4.54	425.10

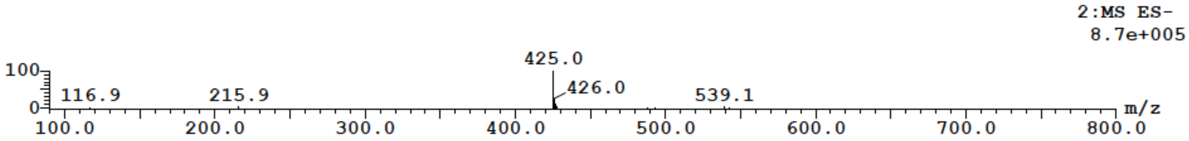

MS of compound **10**

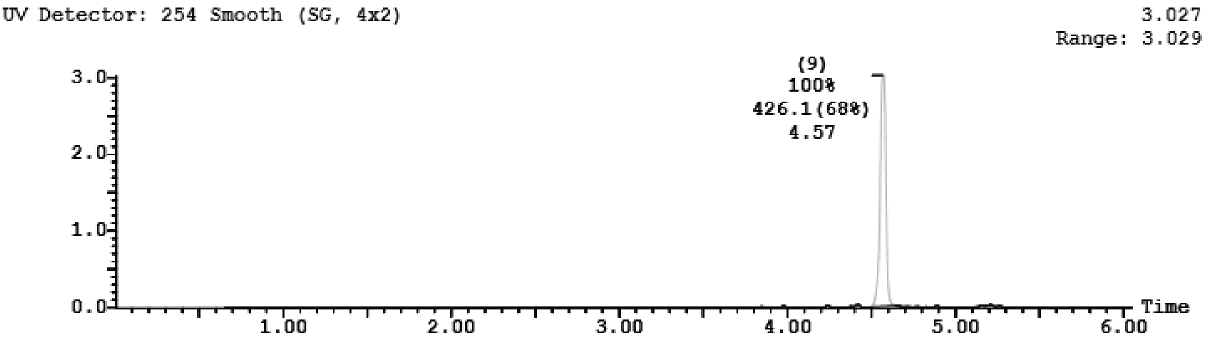

eak Number	Compound	Time	AreaAbs	Area %Total	Width	Height	Mass Found
9	Found	4.57	1e+005	100.00	0	3e+006	426.10

Chromatographic purity analysis of compound **10**

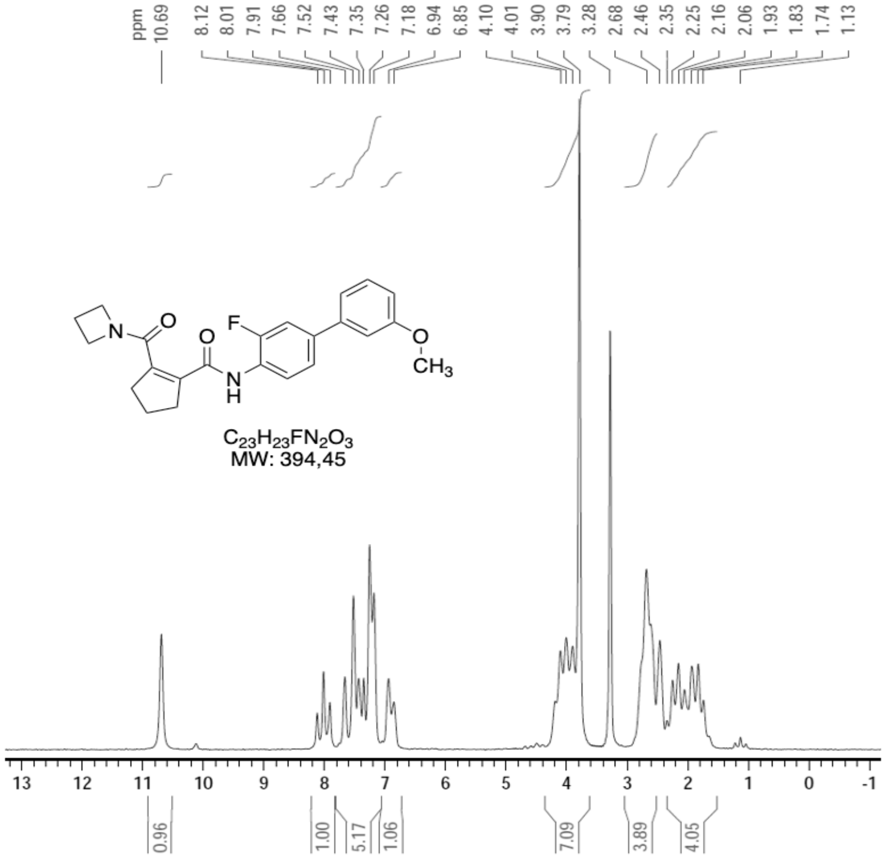

^{1}H-NMR (80 MHz, DMSO-d_6) of compound **11**

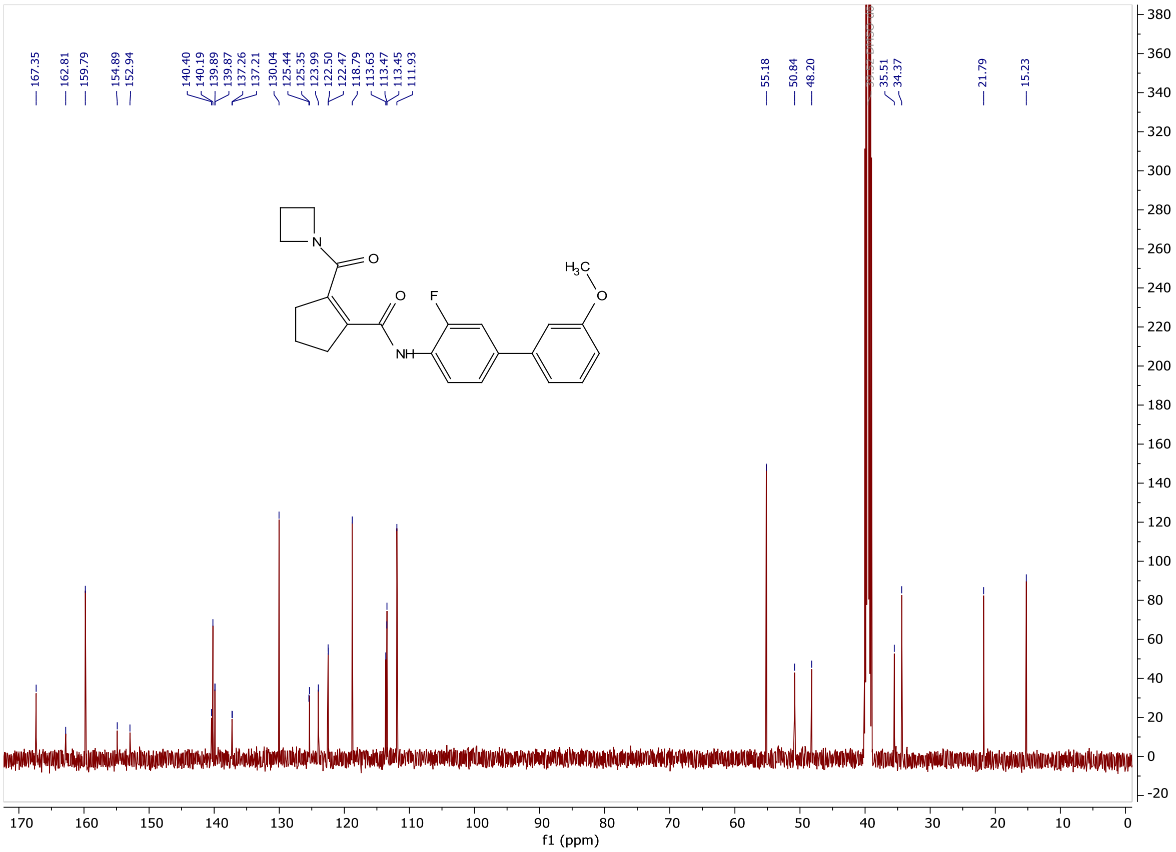

^{13}C-NMR (126 MHz, DMSO-d_6) of compound **11**

Peak ID	Compound	Time	Mass Found
7	Found	4.16	,417.20,395.20

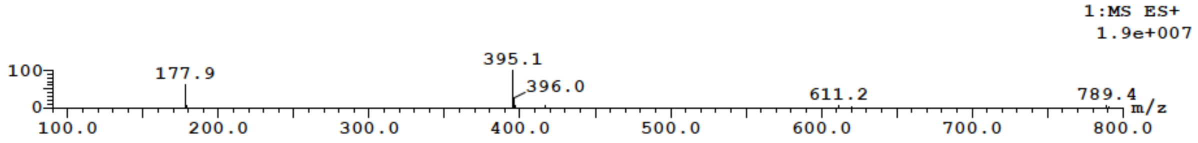

MS of compound **11**

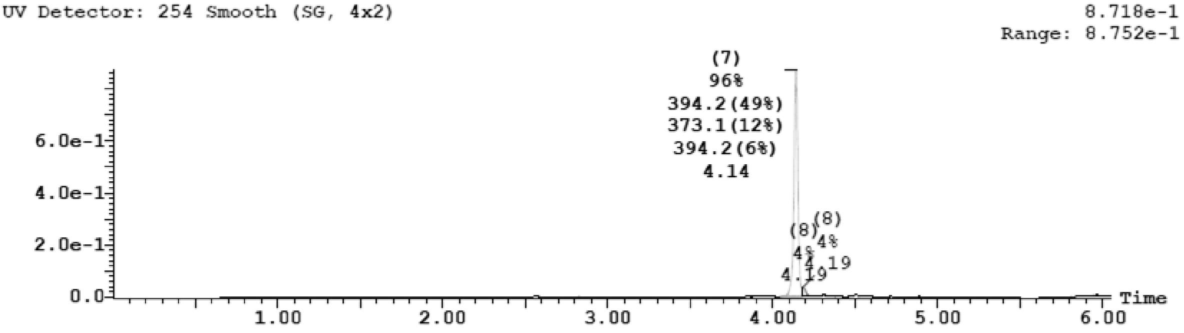

Chromatographic purity analysis of compound **11**

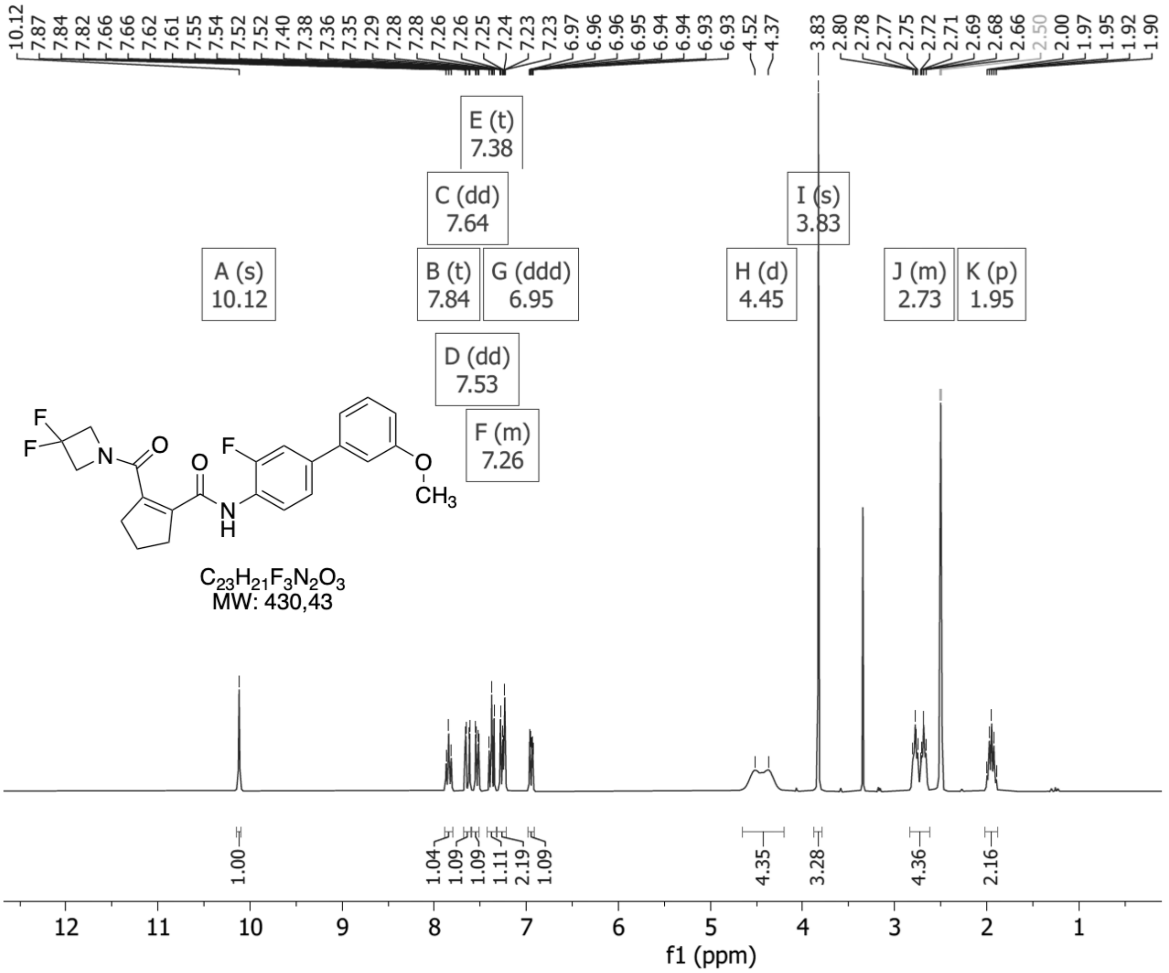

^{1}H-NMR (300 MHz, DMSO-d_6) of compound **12**

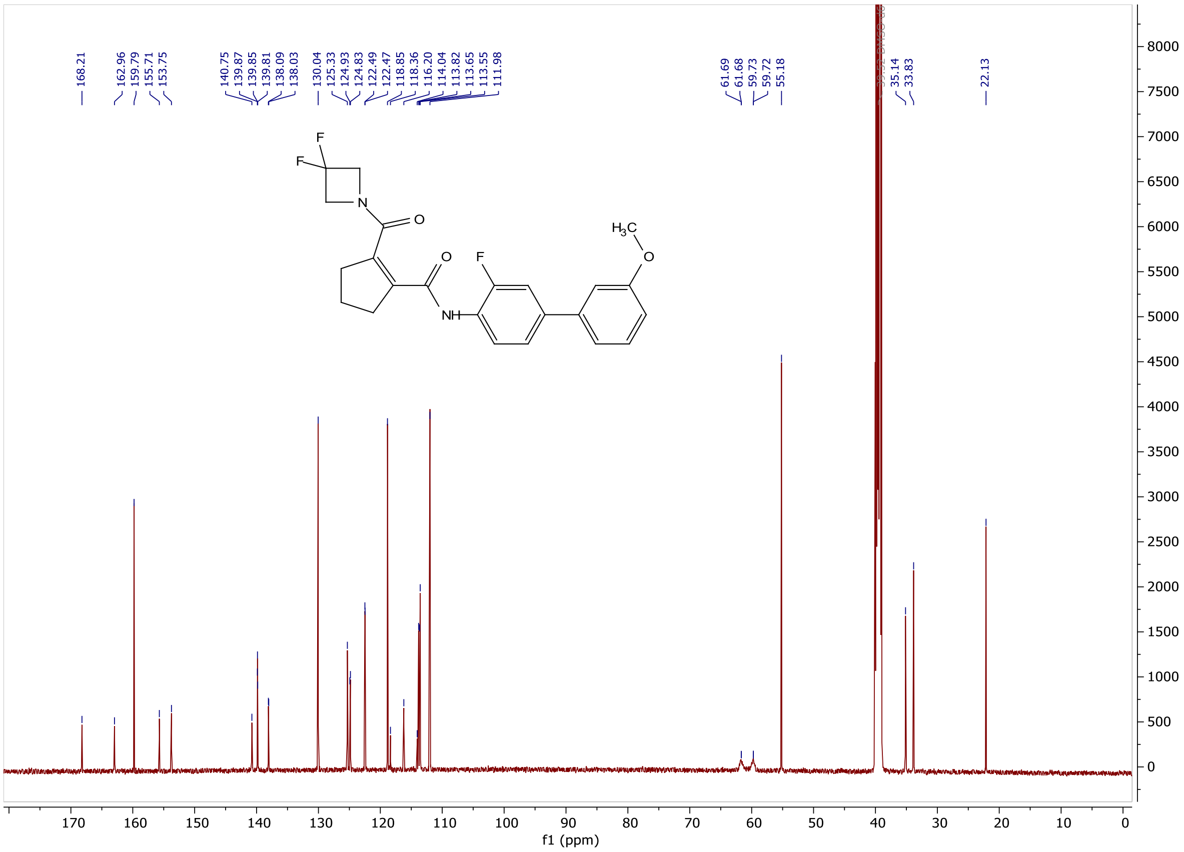

^{13}C-NMR (126 MHz, DMSO-d_6) of compound **12**

Peak ID	Compound	Time	Mass Found
12	Found	4.33	453.10,431.10

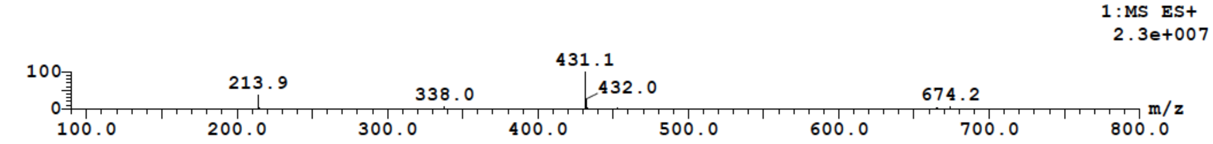

MS of compound **12**

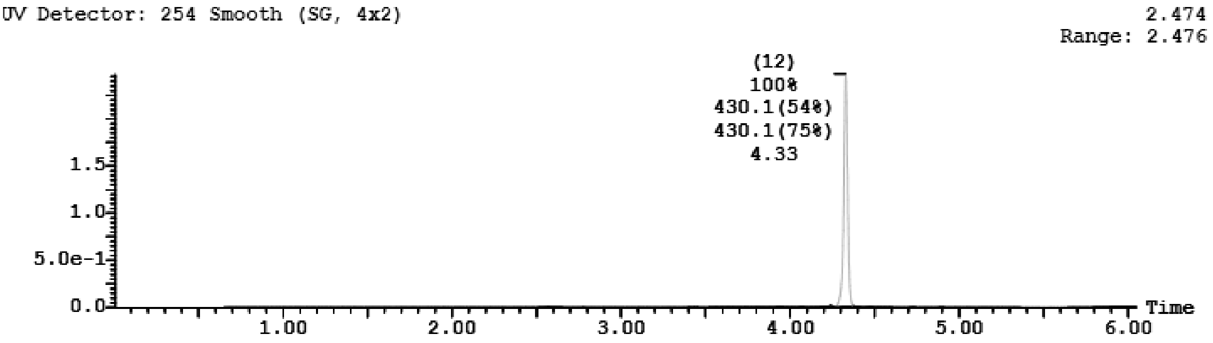

ak Number	Compound	Time	AreaAbs	Area %Total	Width	Height	Mass Found
12	Found	4.33	7e+004	100.00	0	2e+006	430.10, 430.10

Chromatographic purity analysis of compound **12**

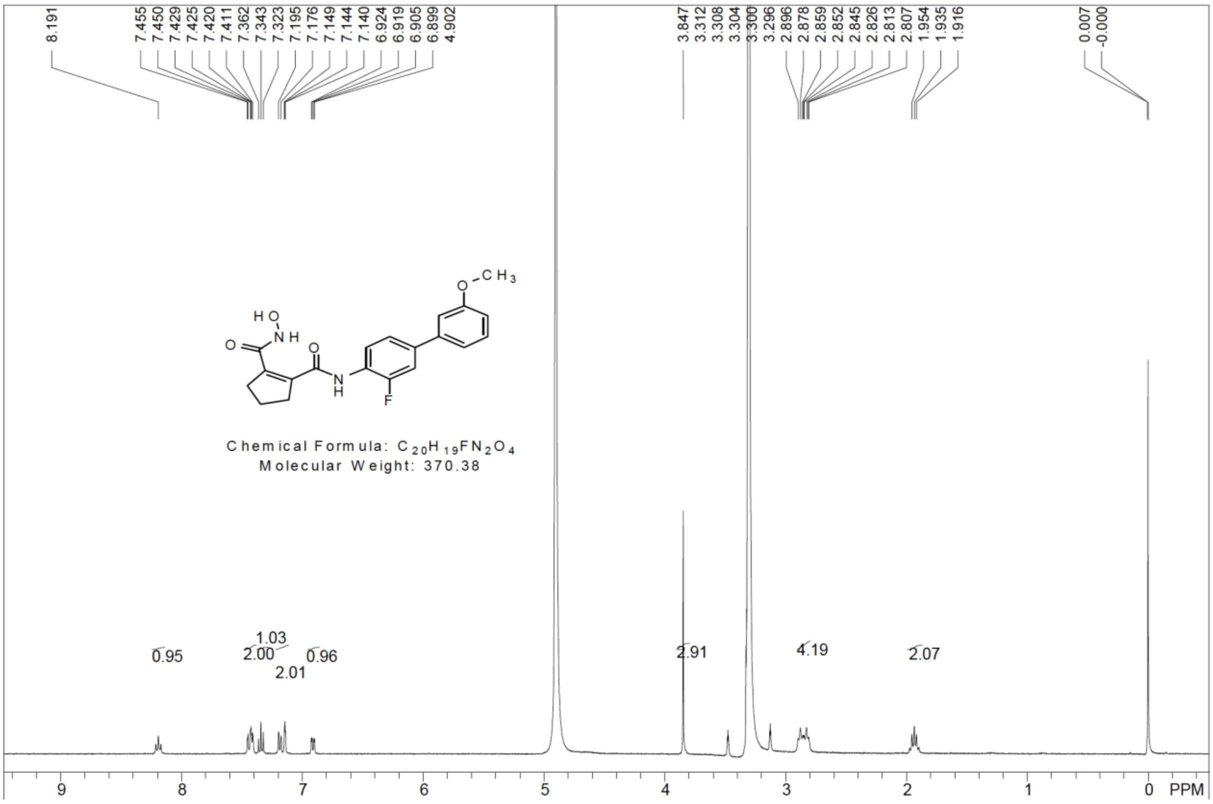

^{1}H-NMR (400 MHz, CD_3OD) of compound **13**

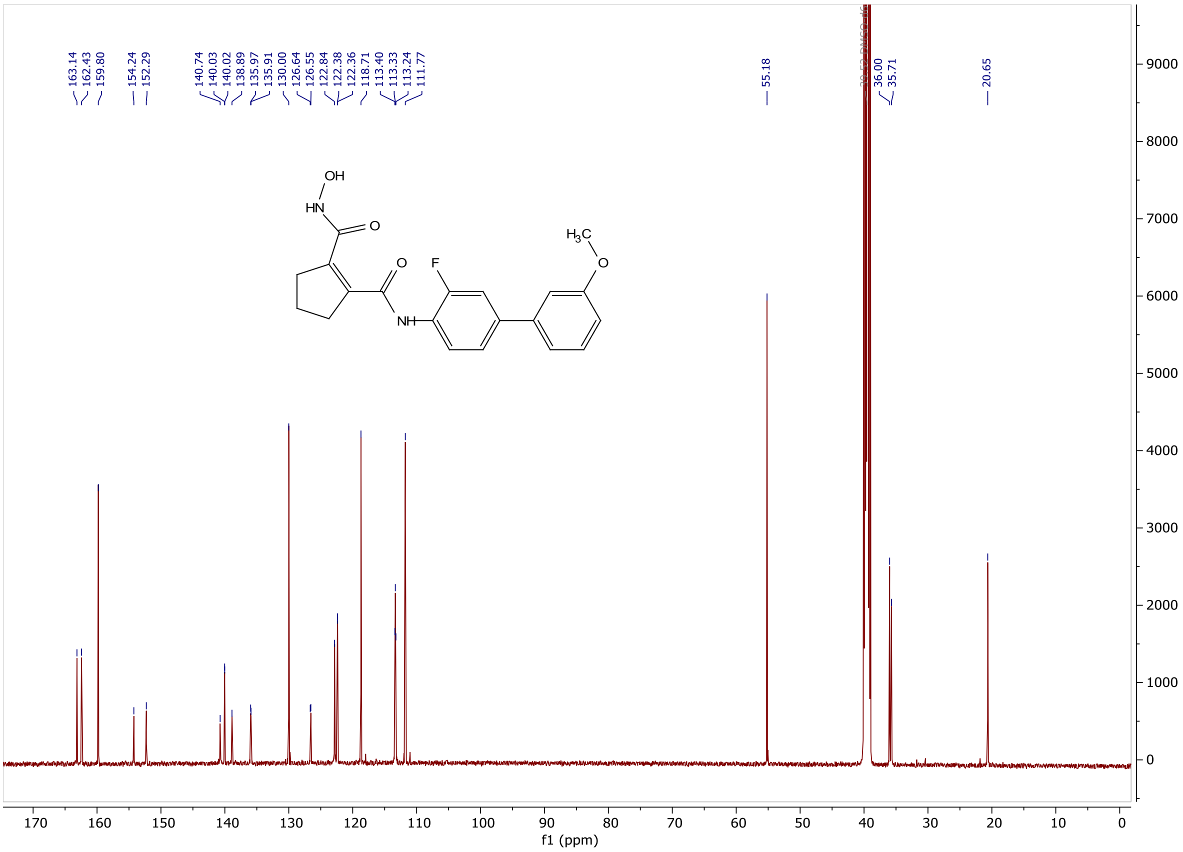

^{13}C-NMR (126 MHz, DMSO-d_6) of compound **13**

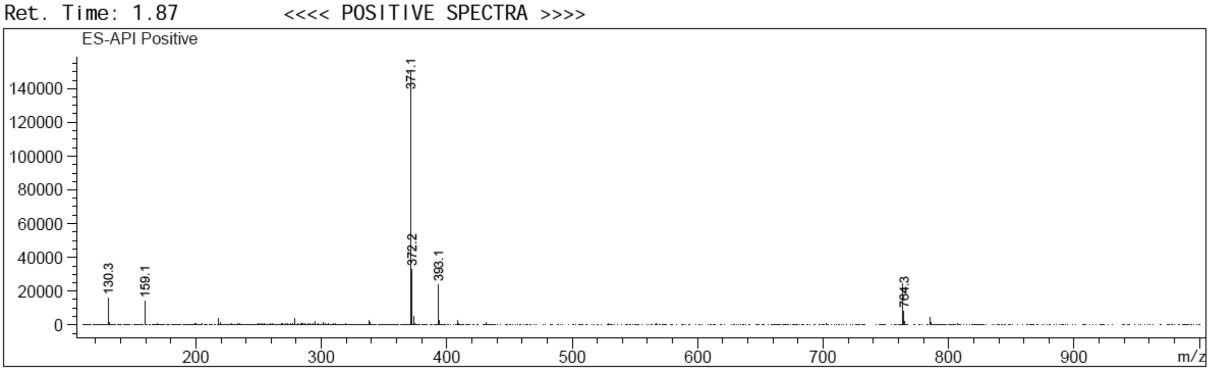

MS of compound **13**

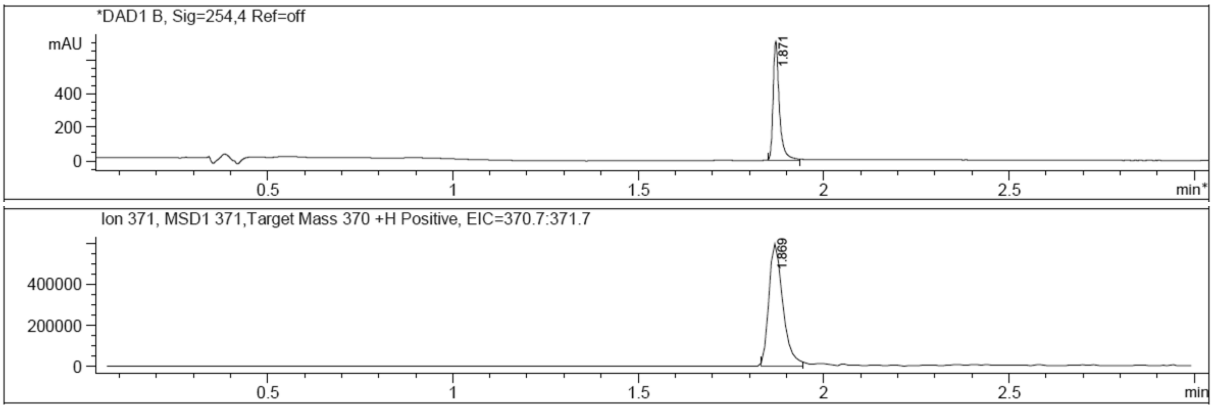

Chromatographic purity analysis of compound **13**

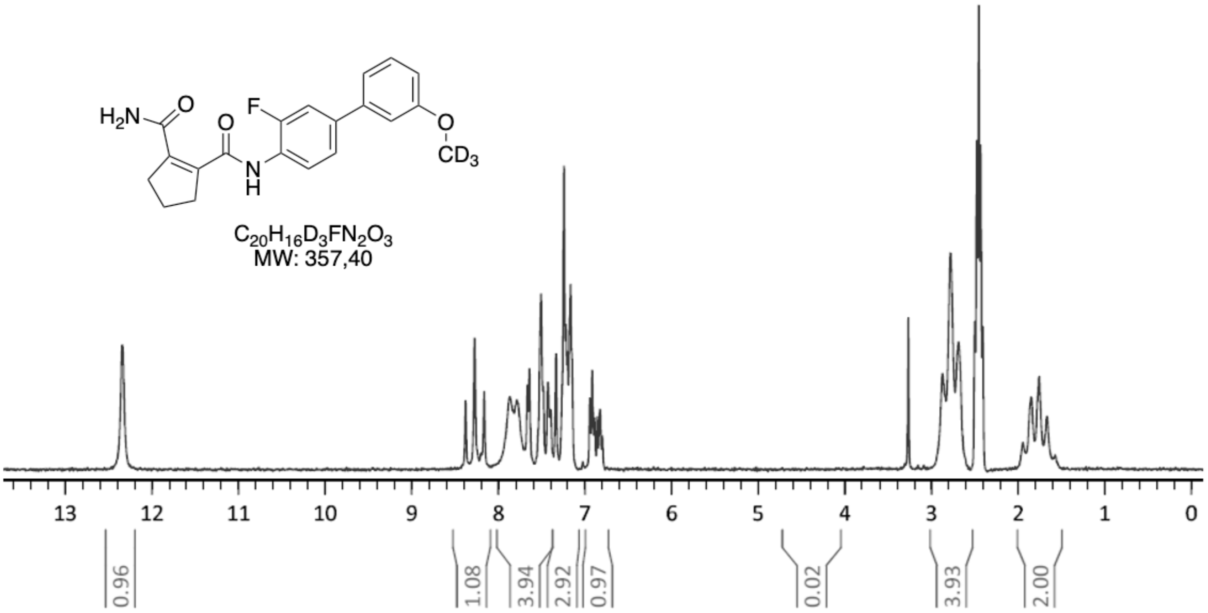

^{1}H-NMR (80 MHz, DMSO-d_6) of compound **15**

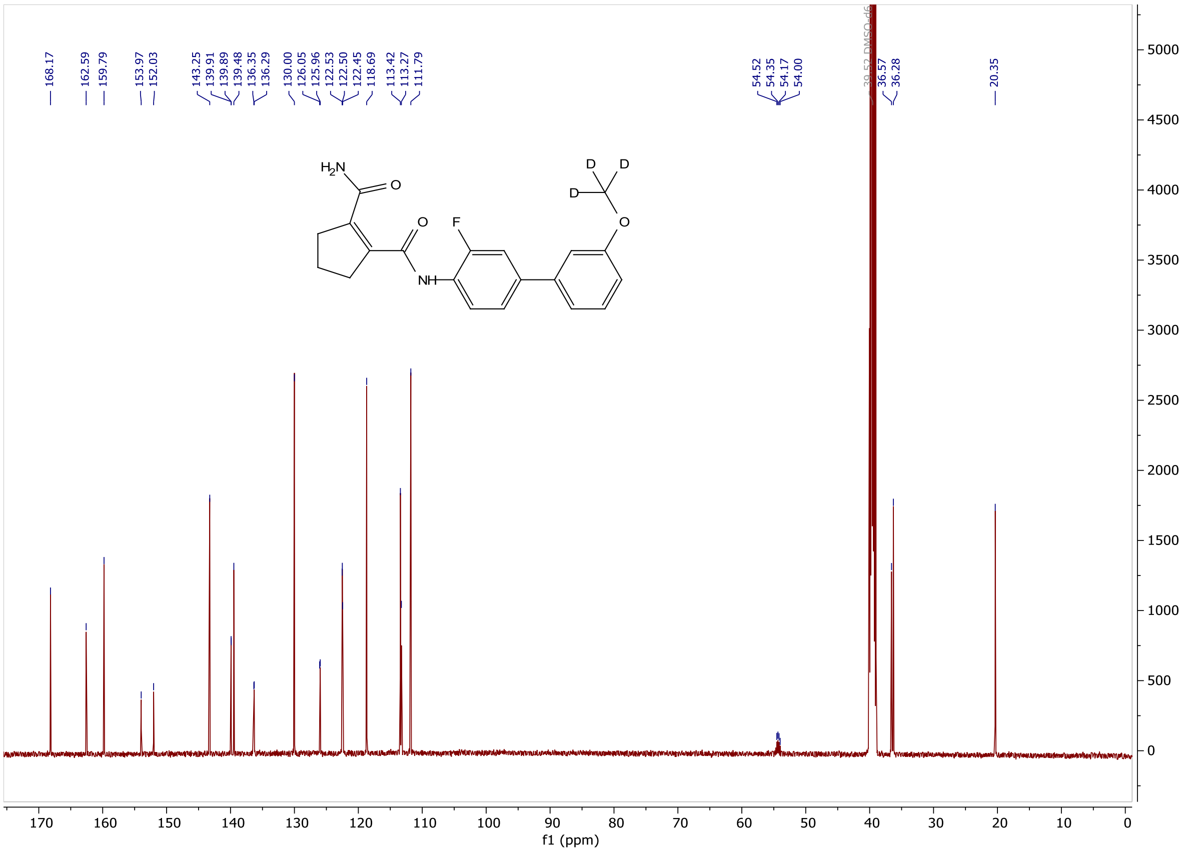

^{13}C-NMR (126 MHz, DMSO-d_6) of compound **15**

Peak ID	Compound	Time	Mass Found
3	Found	4.10	358.15,,359.14

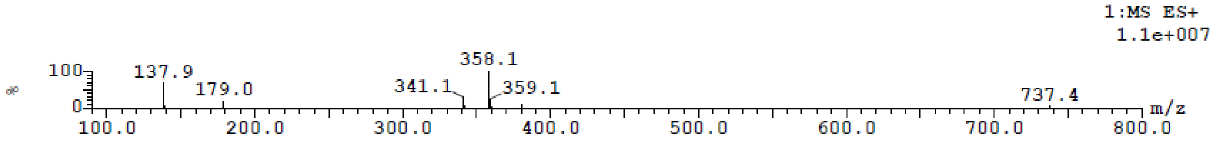

MS of compound **15**

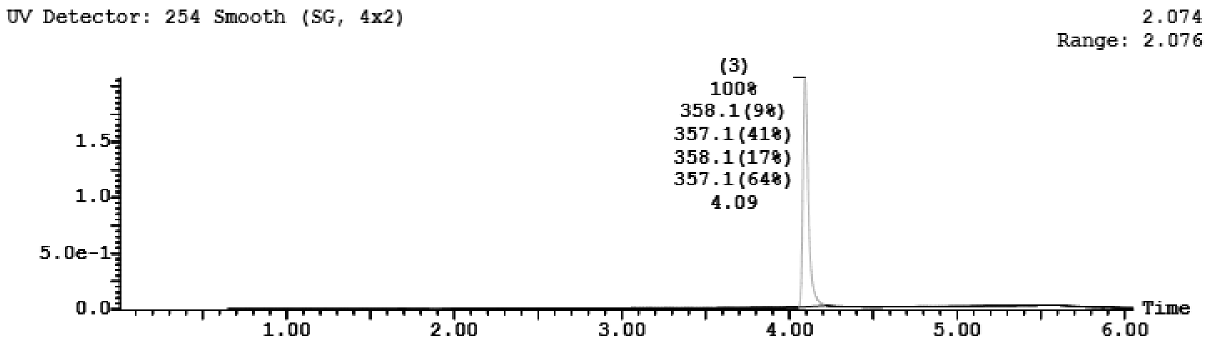

eak Number	Compound	Time	AreaAbs	Area %Total	Width	Height	Mass Found
3	Found	4.09	8e+004	100.00	0	2e+006	357.15, 358.14

Chromatographic purity analysis of compound **15**

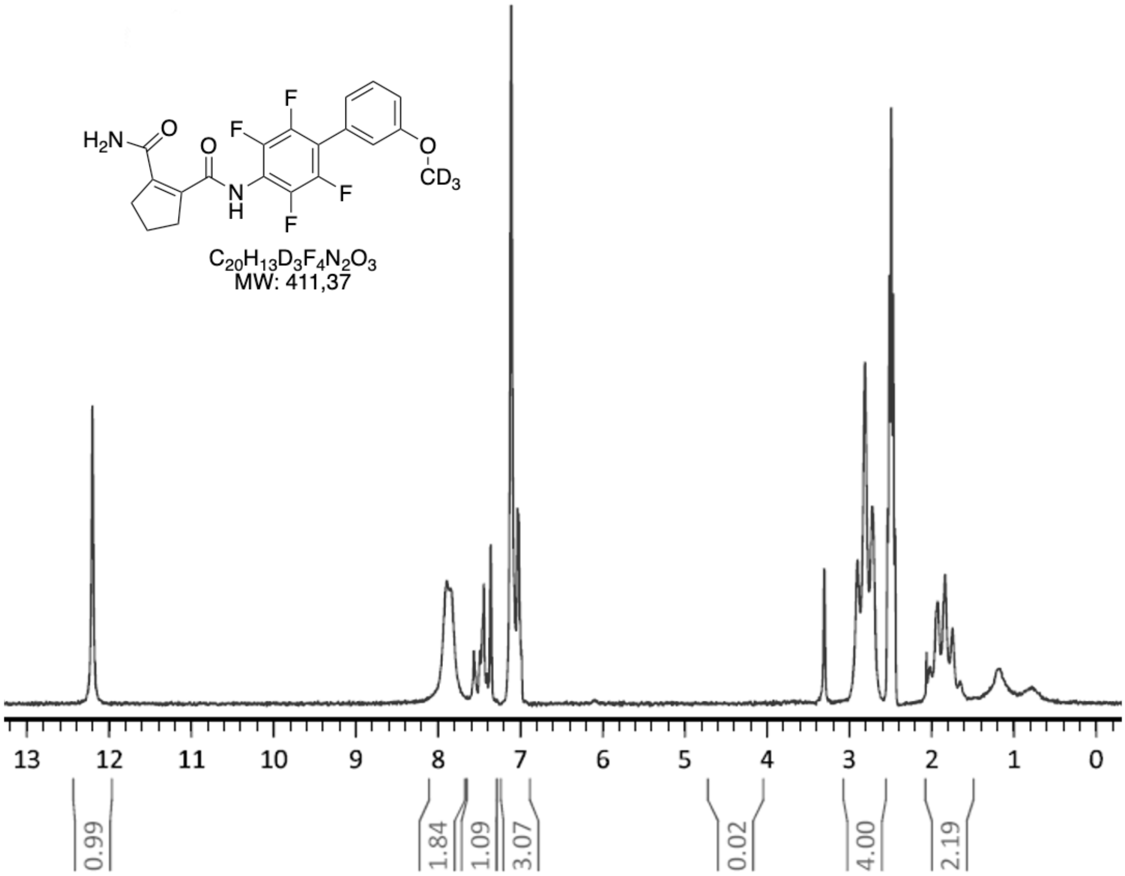

^{1}H-NMR (80 MHz, DMSO-d_6) of compound **17**

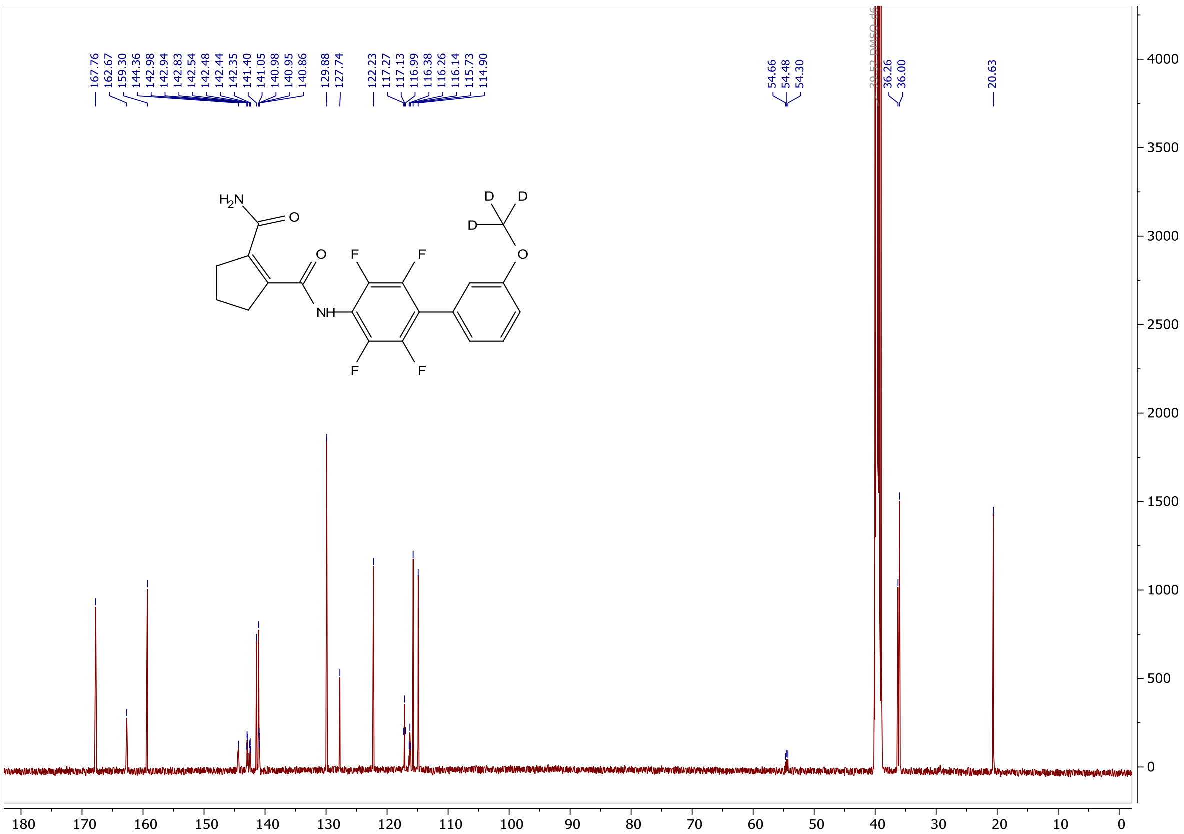

^{13}C-NMR (126 MHz, DMSO-d_6) of compound **17**

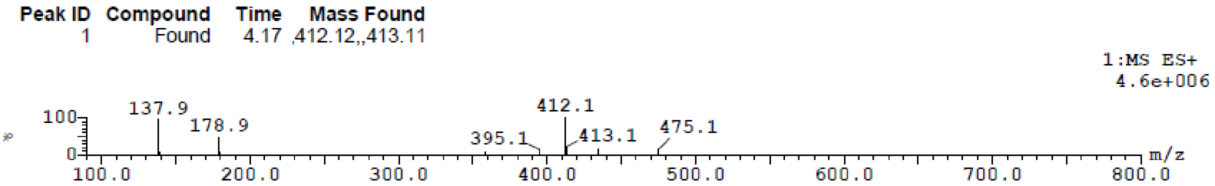

MS of compound **17**

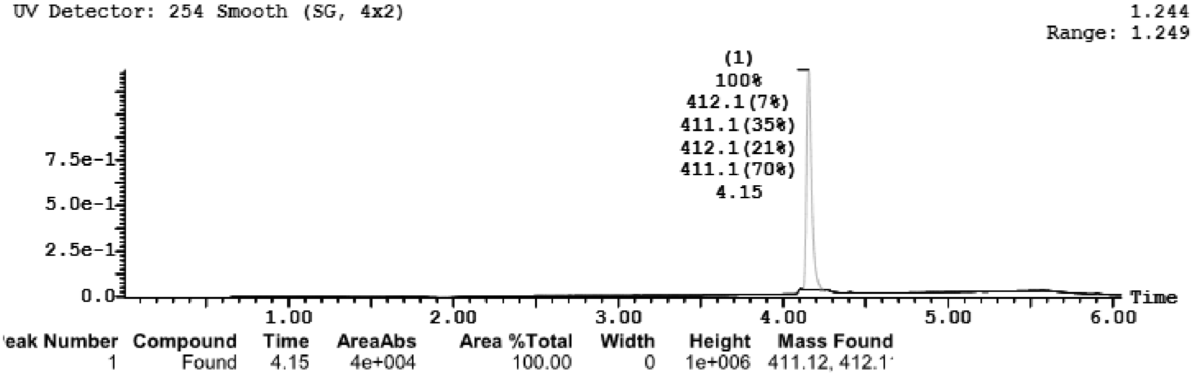

Chromatographic purity analysis of compound **17**

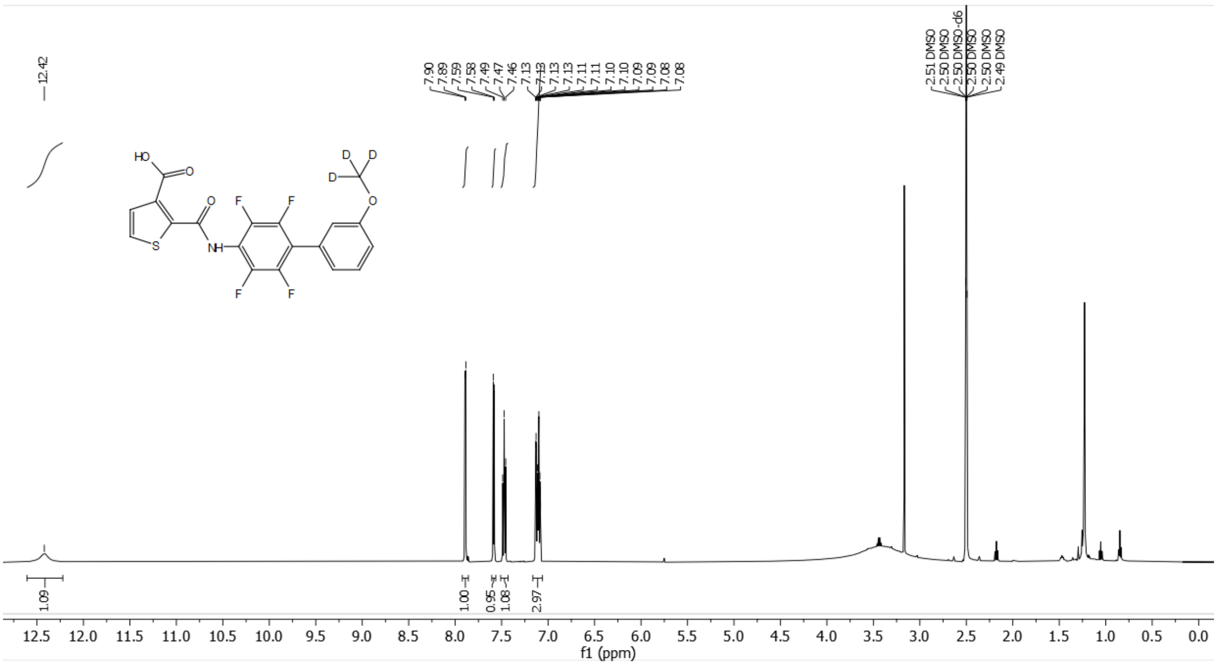

^{1}H-NMR (500 MHz, DMSO-d_6) of compound **22**

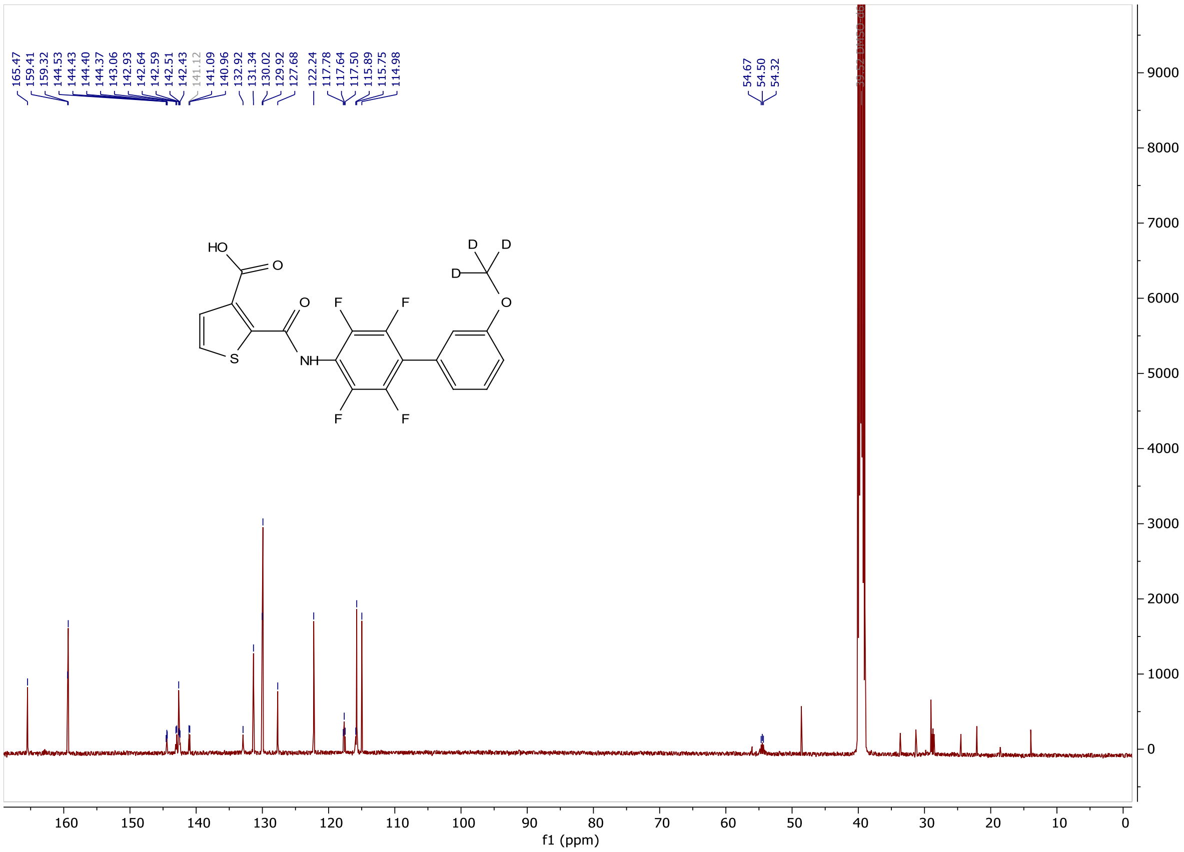

^{13}C-NMR (126 MHz, DMSO-d_6) of compound **22**

Peak ID	Compound	Time	Mass Found
31	Found	3.81	451.05,429.05

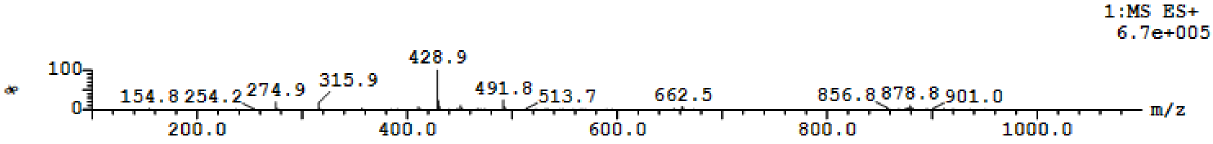

MS of compound **22**

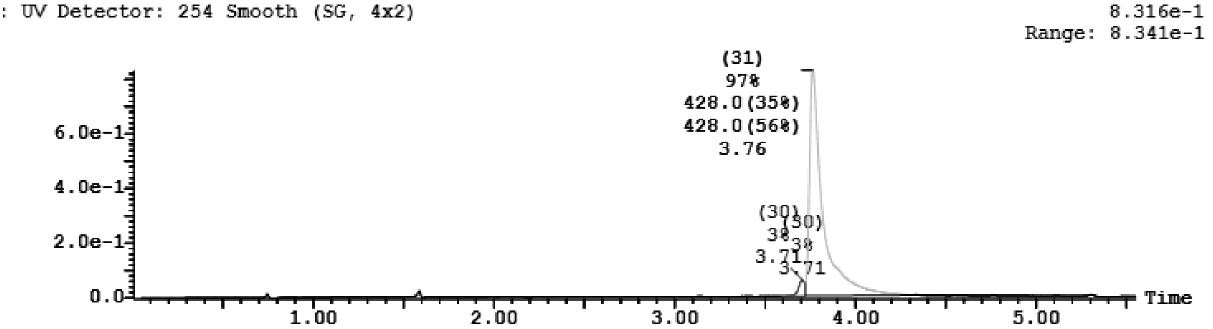

Peak Number	Compound	Time	AreaAbs	Area %Total	Width	Height	Mass Found
30		3.71	2e+003	3.10	0	6e+004	

Chromatographic purity analysis of compound **22**

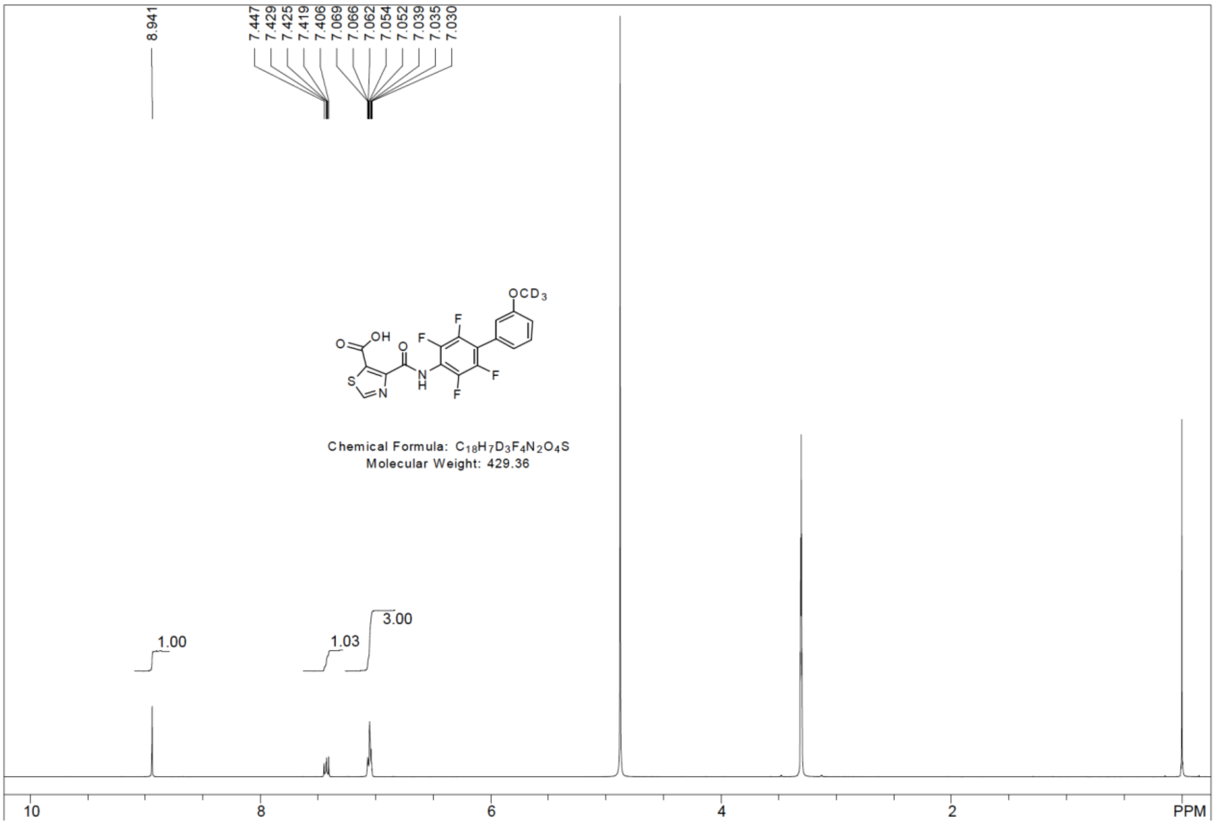

^{1}H-NMR (400 MHz, CD_3OD) of compound **24**

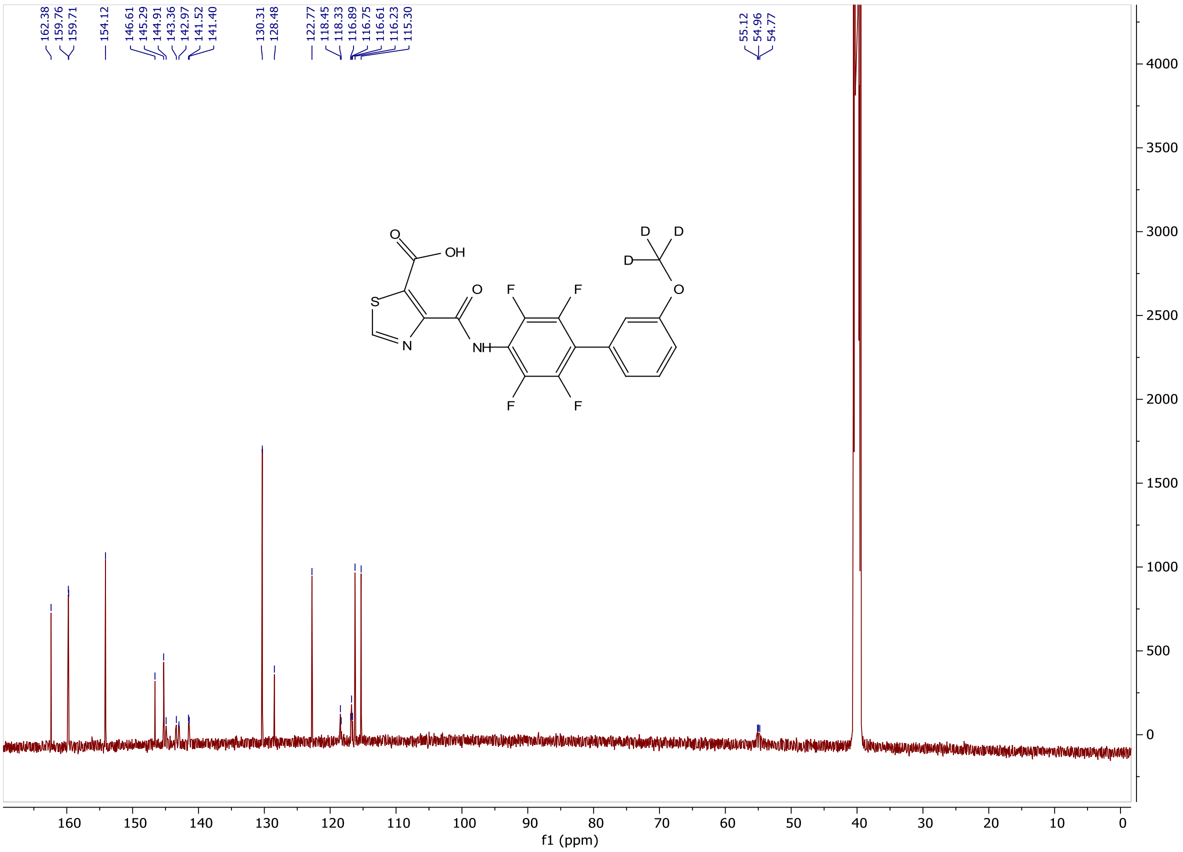

^{13}C-NMR (126 MHz, DMSO-d_6) of compound **24**

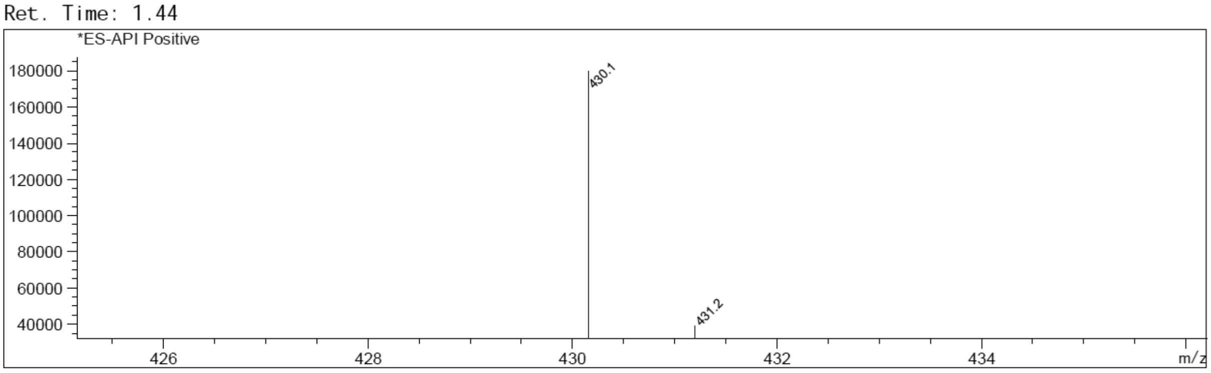

MS of compound **24**

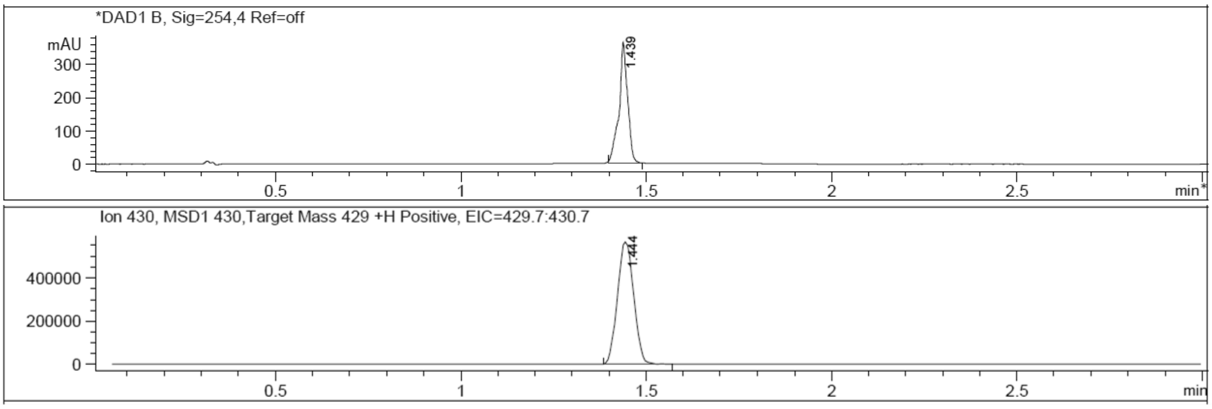

Chromatographic purity analysis of compound **24**

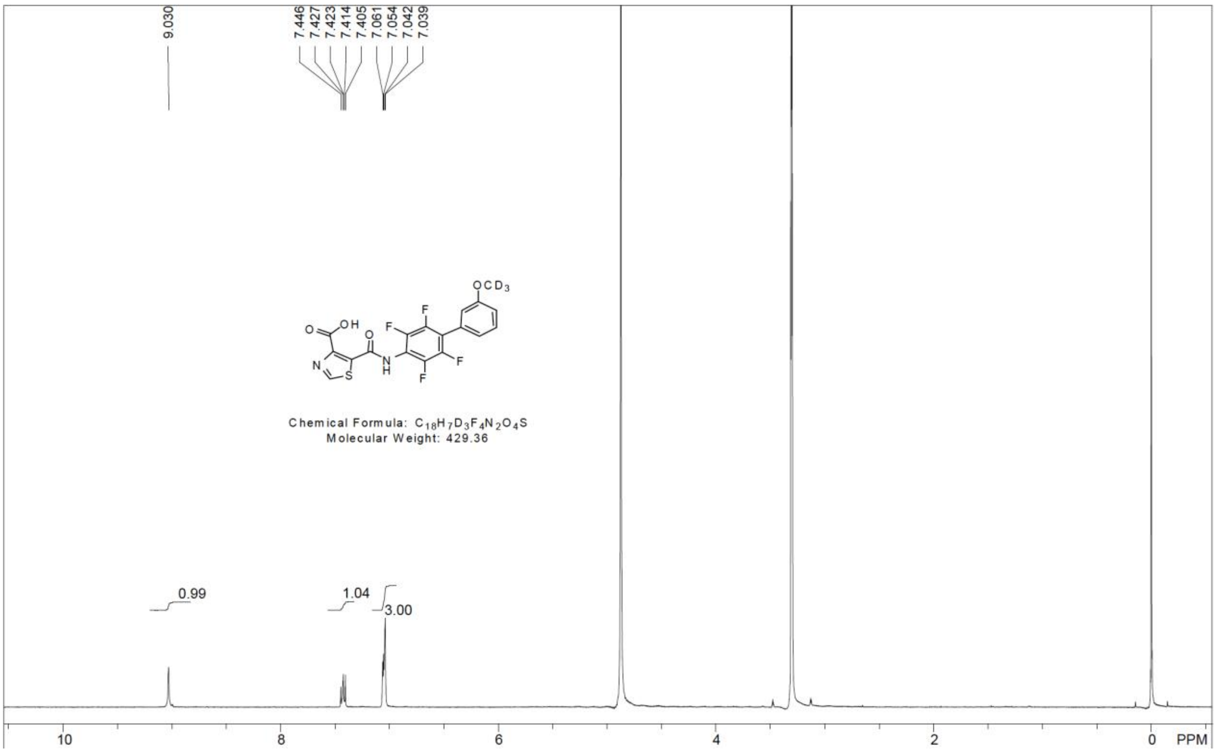

^{1}H-NMR (400 MHz, CD_3OD) of compound **25**

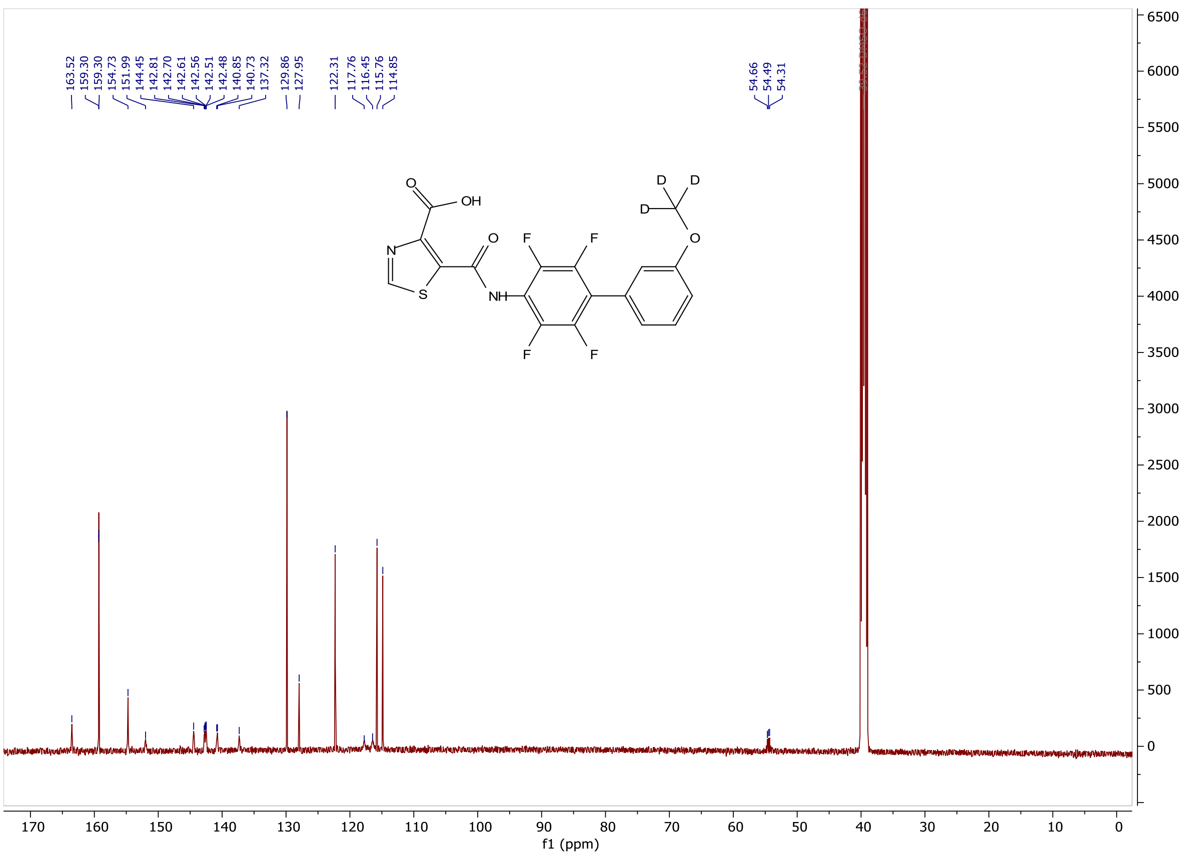

^{13}C-NMR (126 MHz, DMSO-d_6) of compound **25**

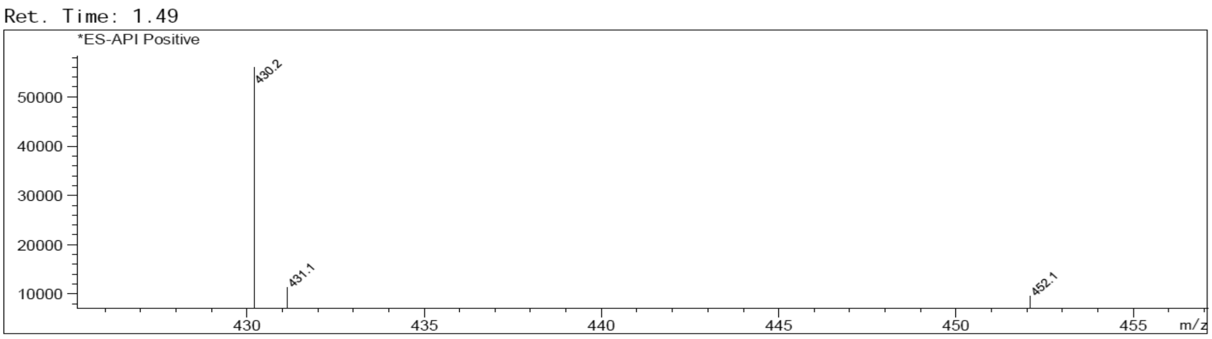

MS of compound **25**

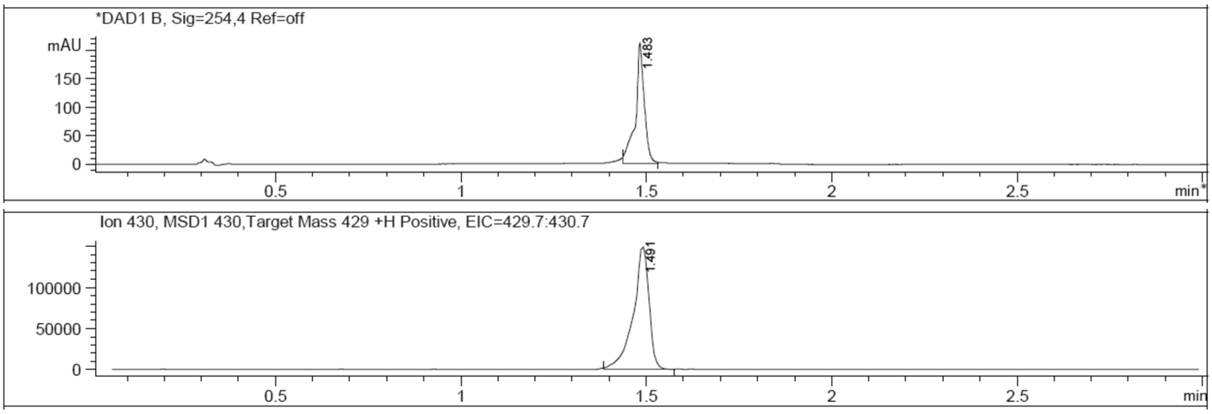

Chromatographic purity analysis of compound **25**

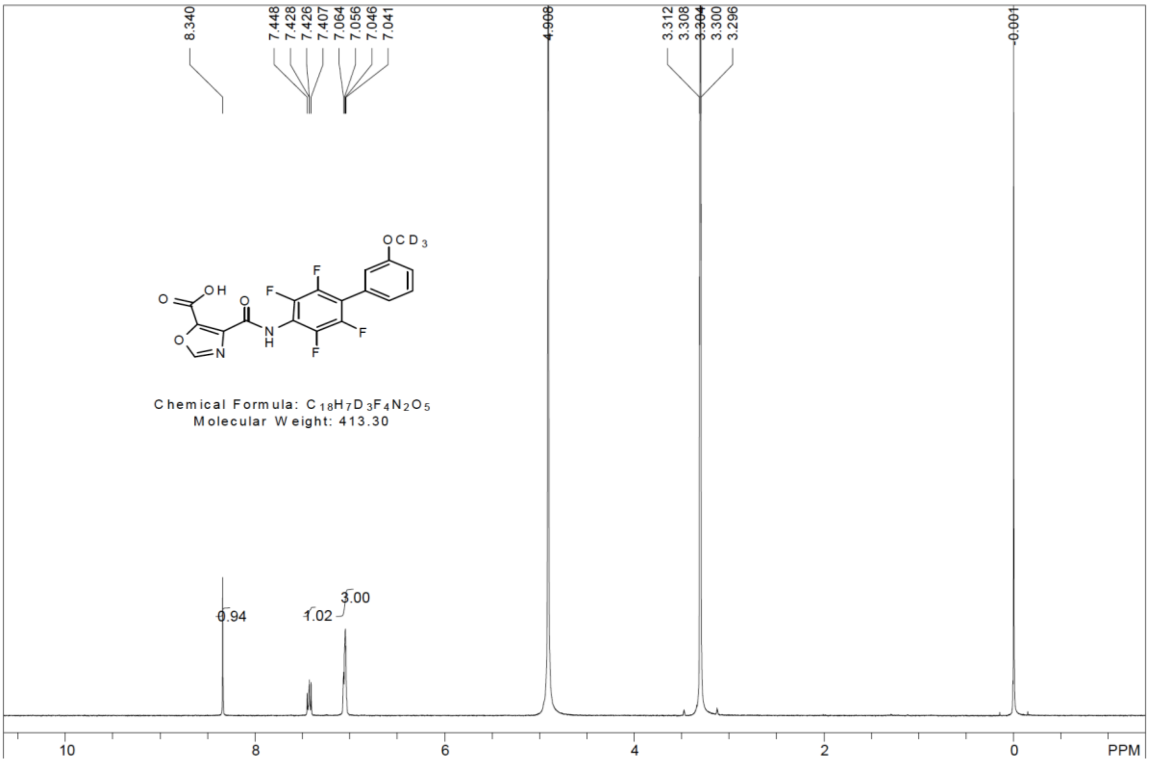

^{1}H-NMR (400 MHz, CD_3OD) of compound **26**

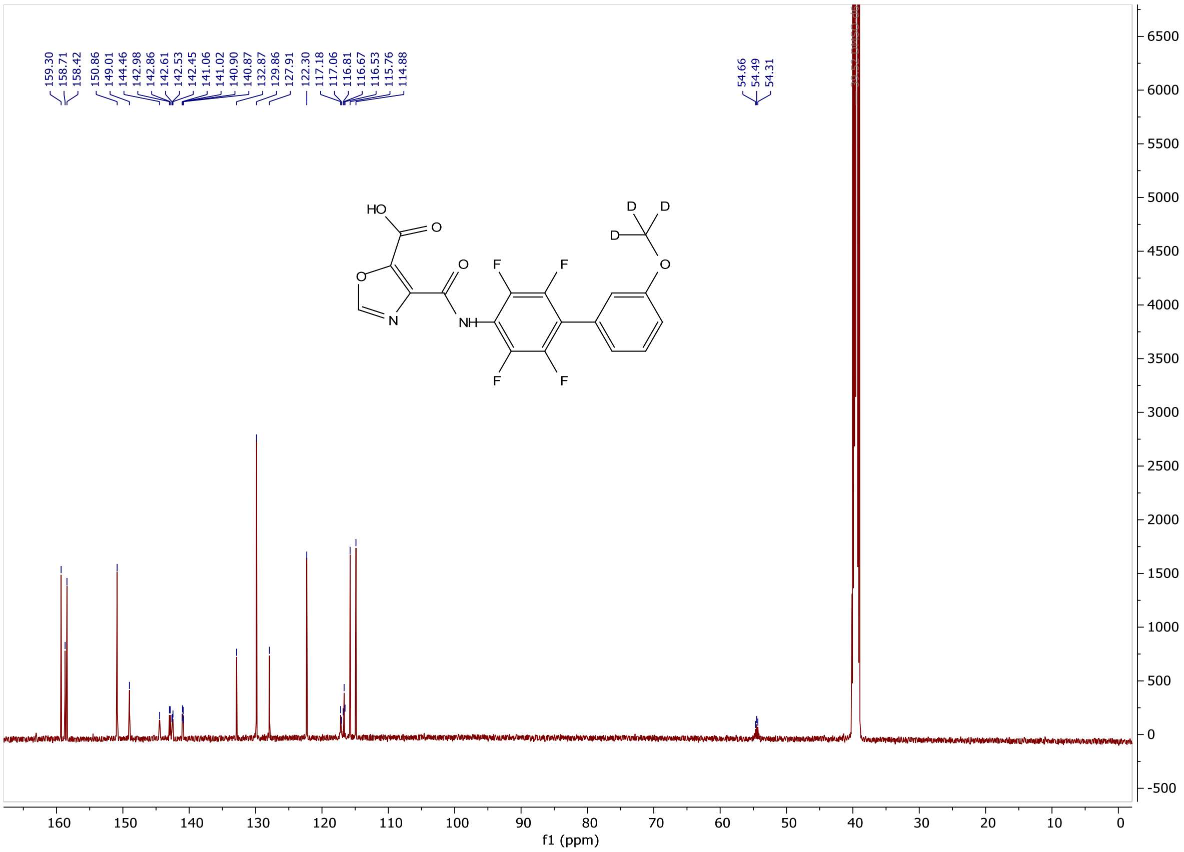

^{13}C-NMR (126 MHz, DMSO-d_6) of compound **26**

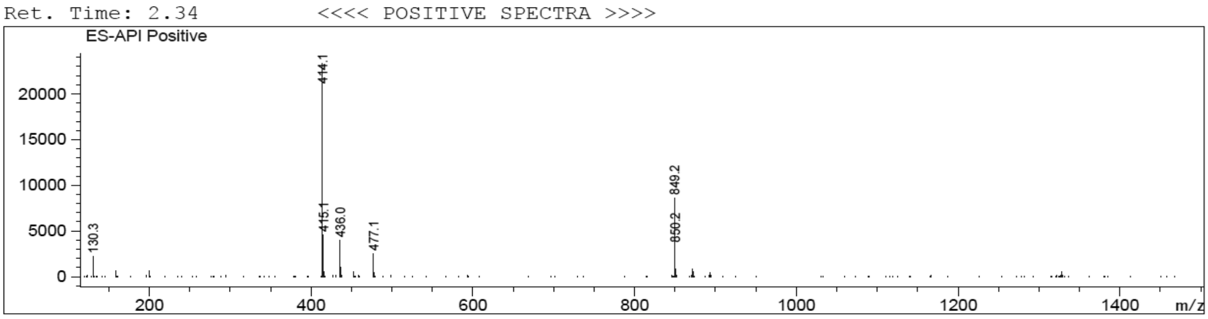

MS of compound **26**

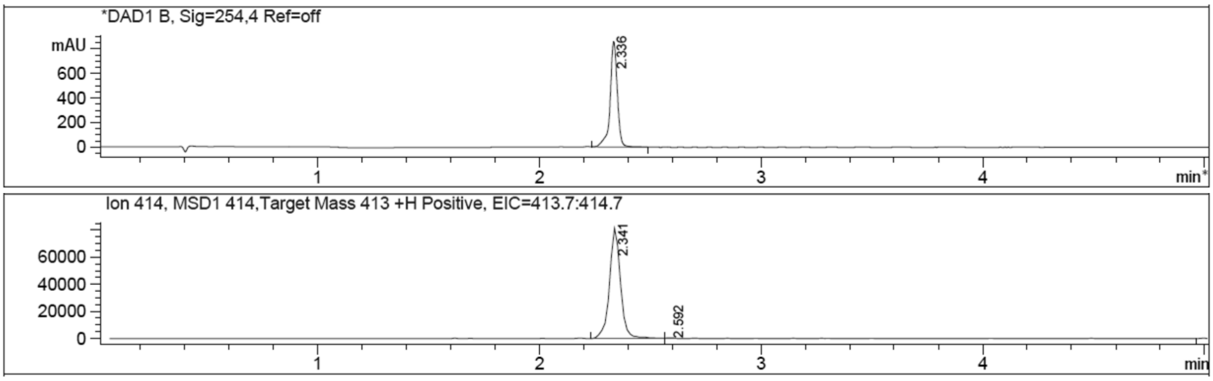

Chromatographic purity analysis of compound **26**

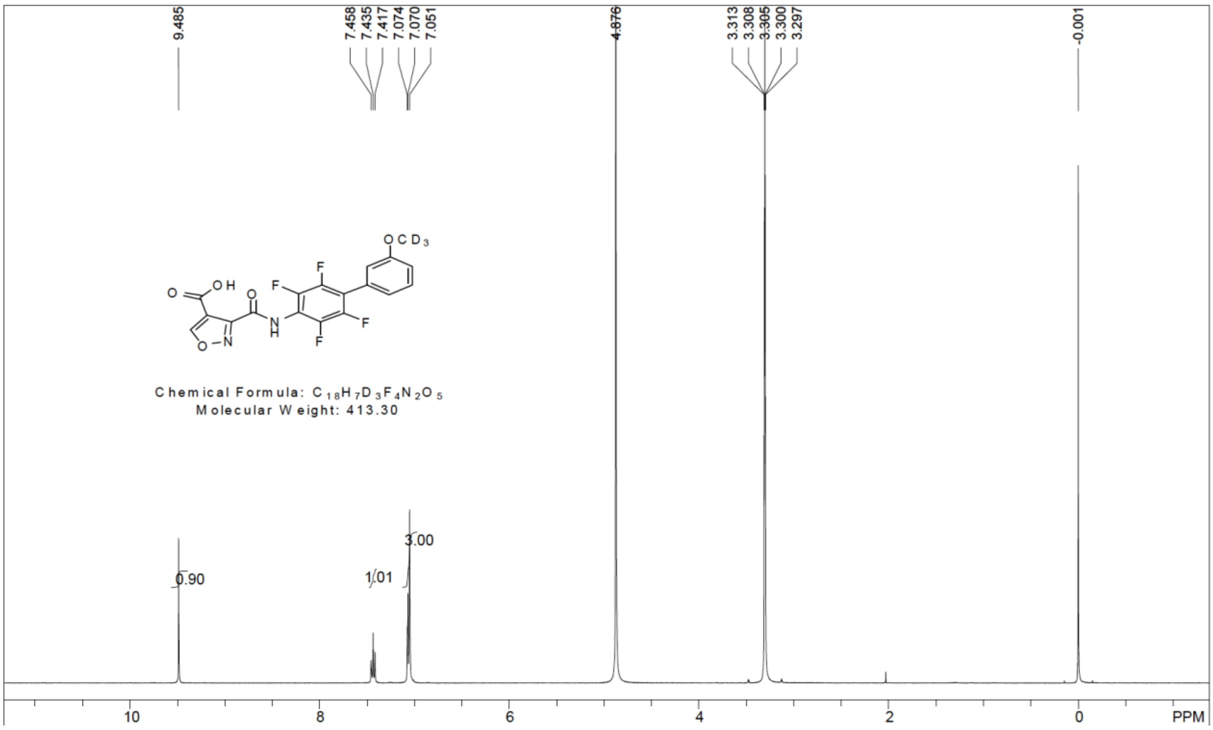

^{1}H-NMR (400 MHz, CD_3OD) of compound **27**

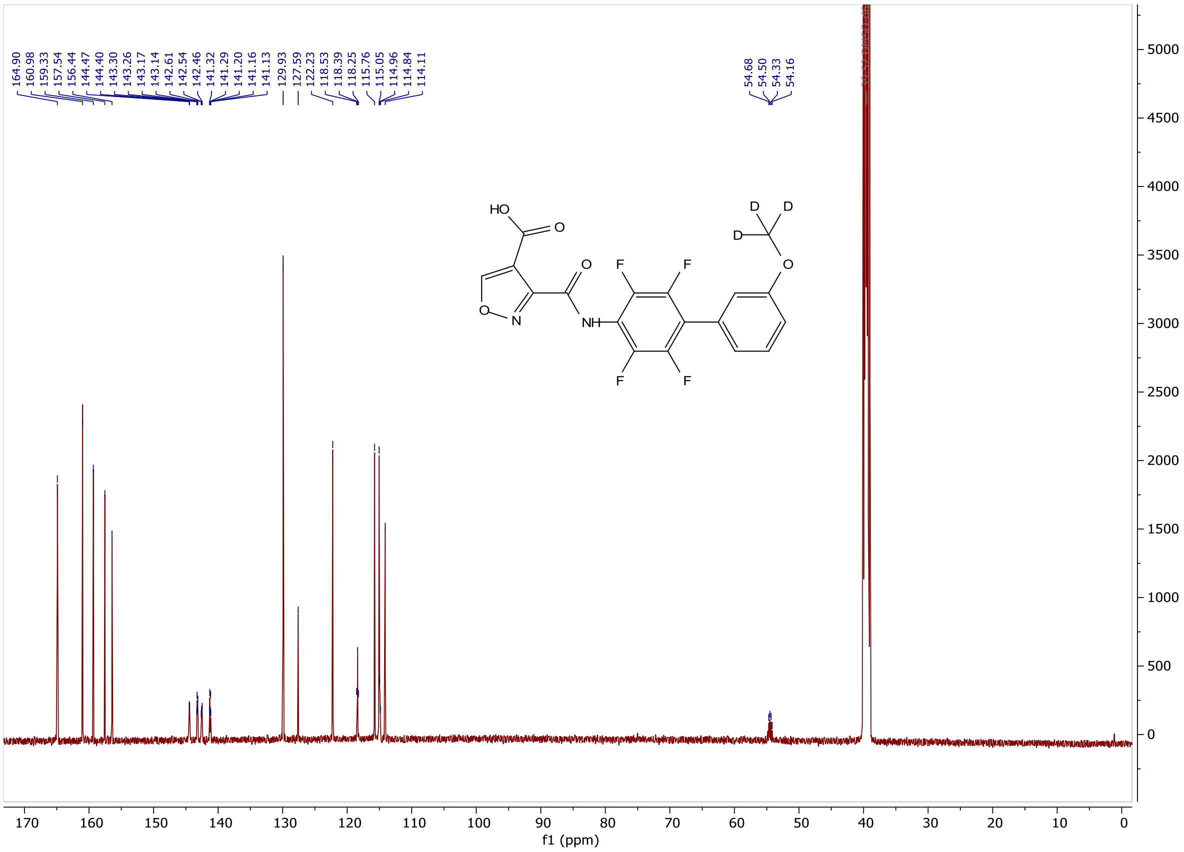

^{13}C-NMR (126 MHz, DMSO-d_6) of compound **27**

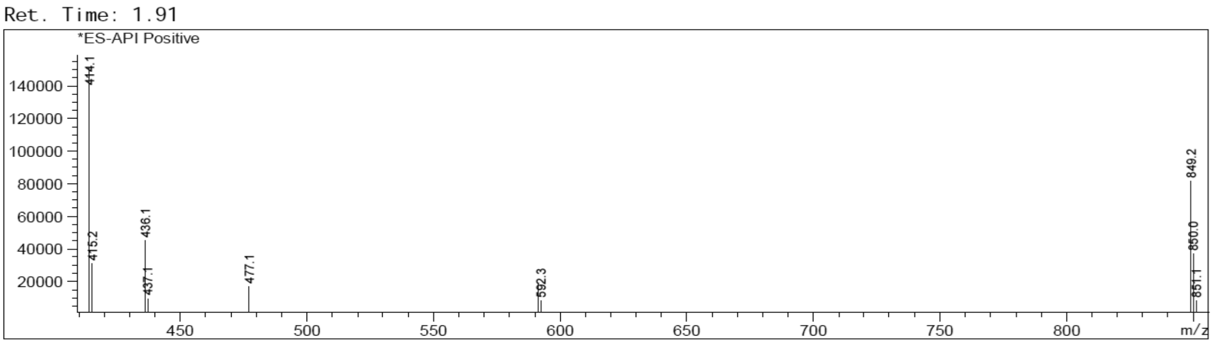

MS of compound **27**

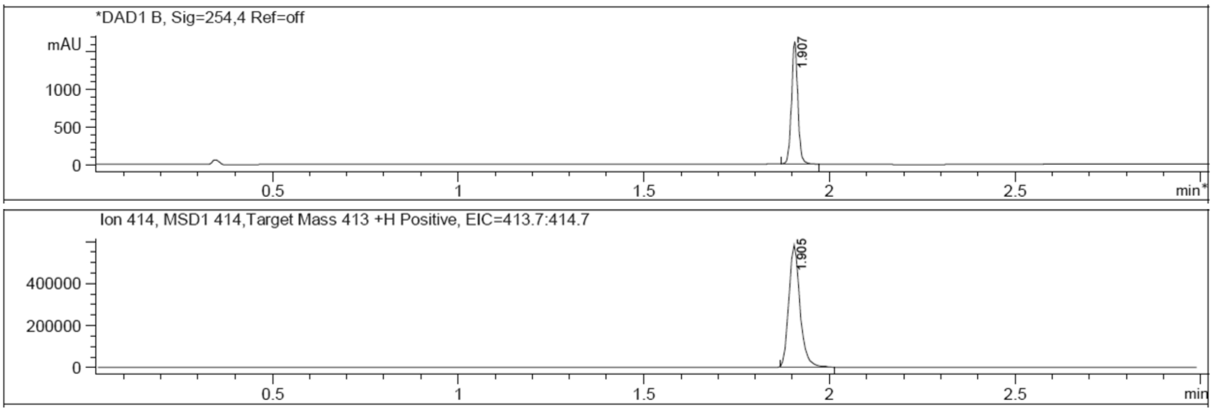

Chromatographic purity analysis of compound **27**

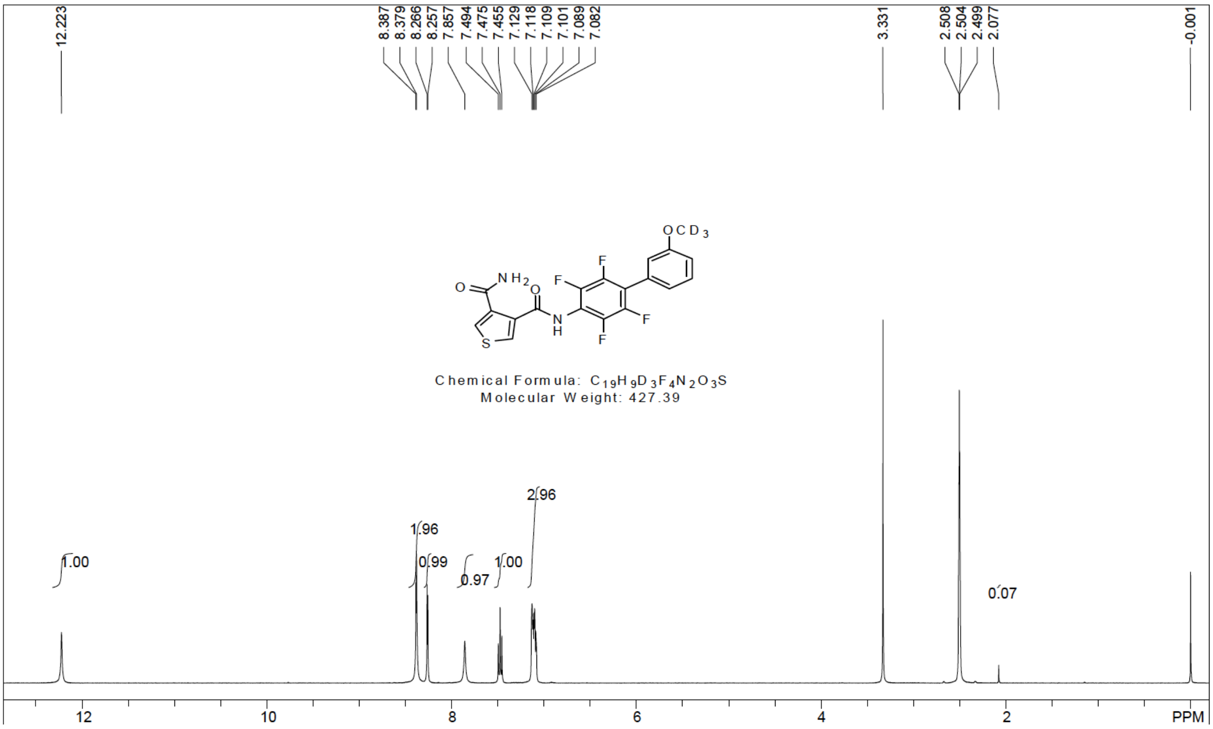

^{1}H-NMR (400 MHz, DMSO-d_6) of compound **28**

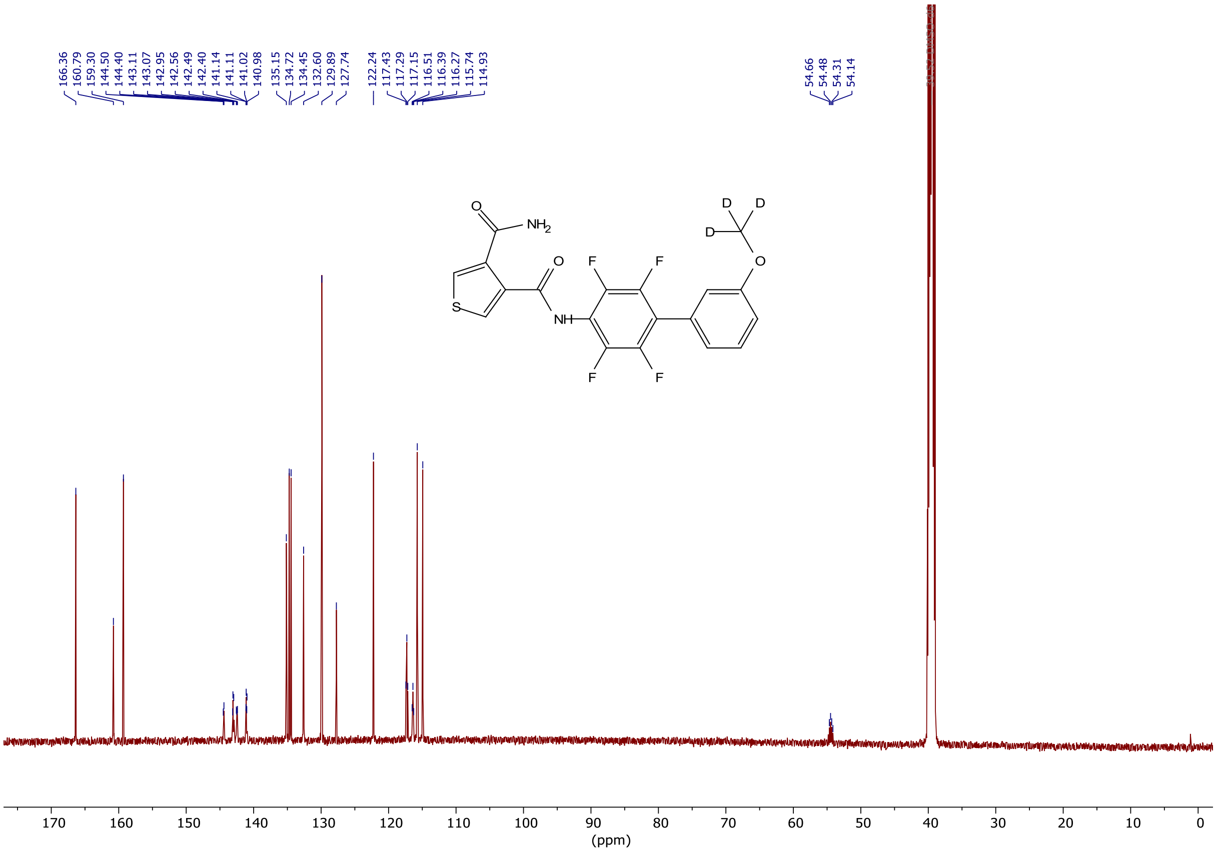

^{13}C-NMR (126 MHz, DMSO-d_6) of compound **28**

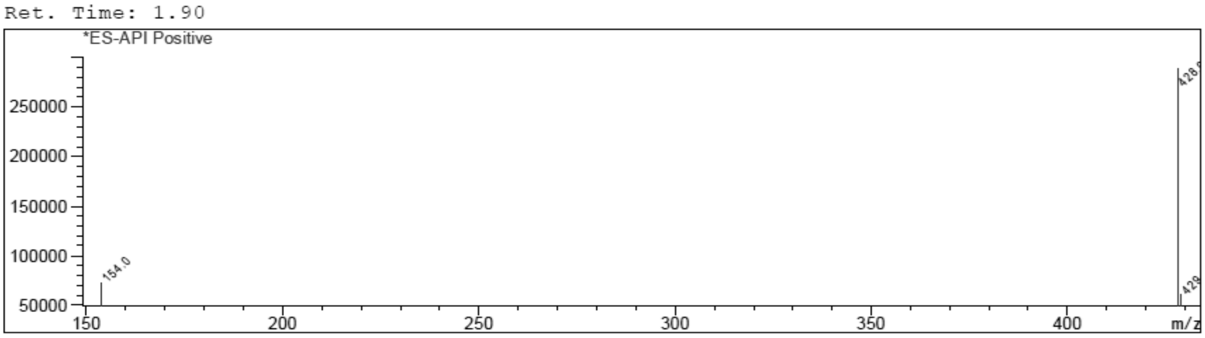

MS of compound **28**

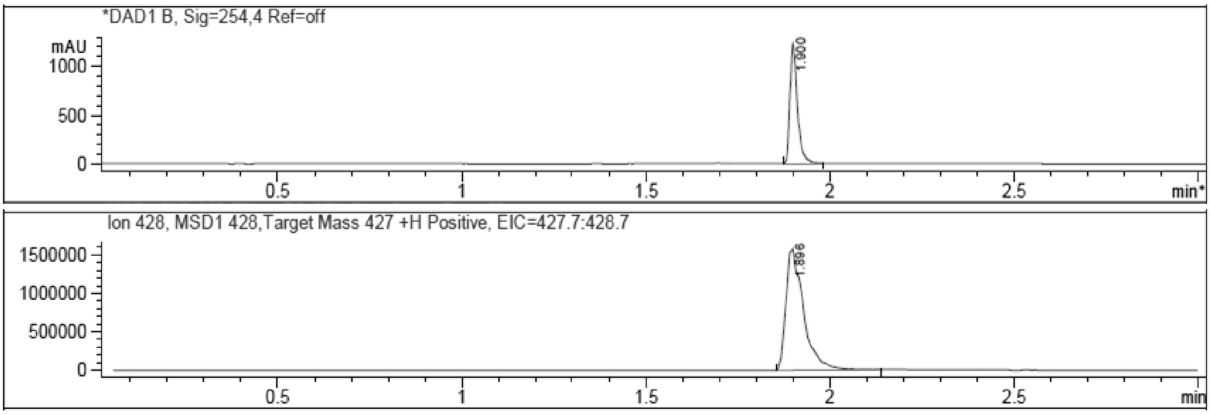

Chromatographic purity analysis of compound **28**

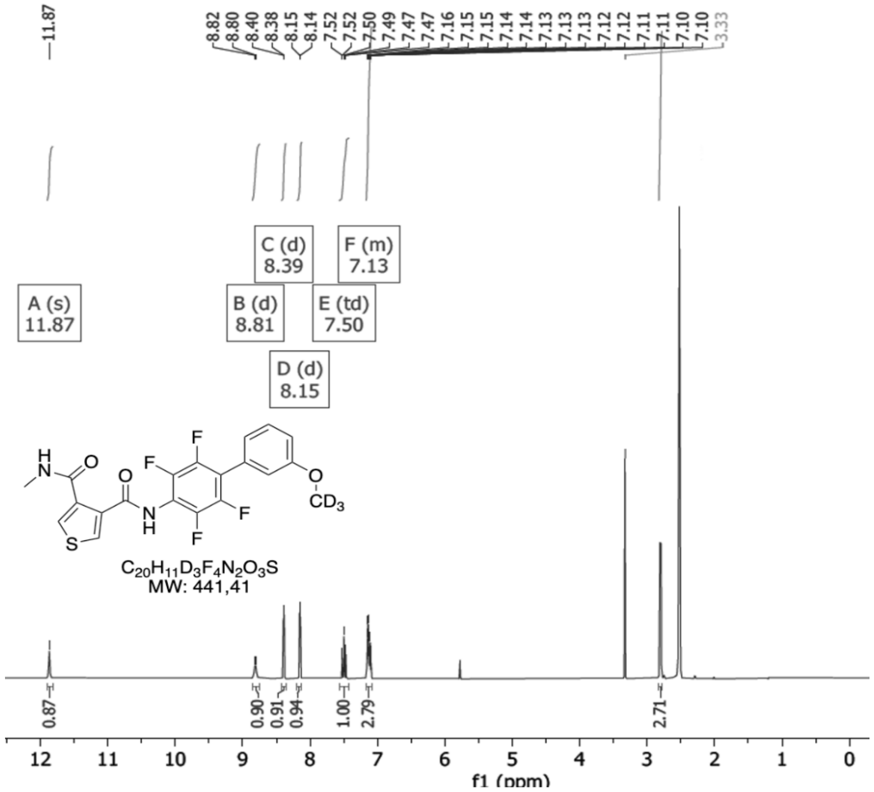

^{1}H-NMR (300 MHz, DMSO-d_6) of compound **29**

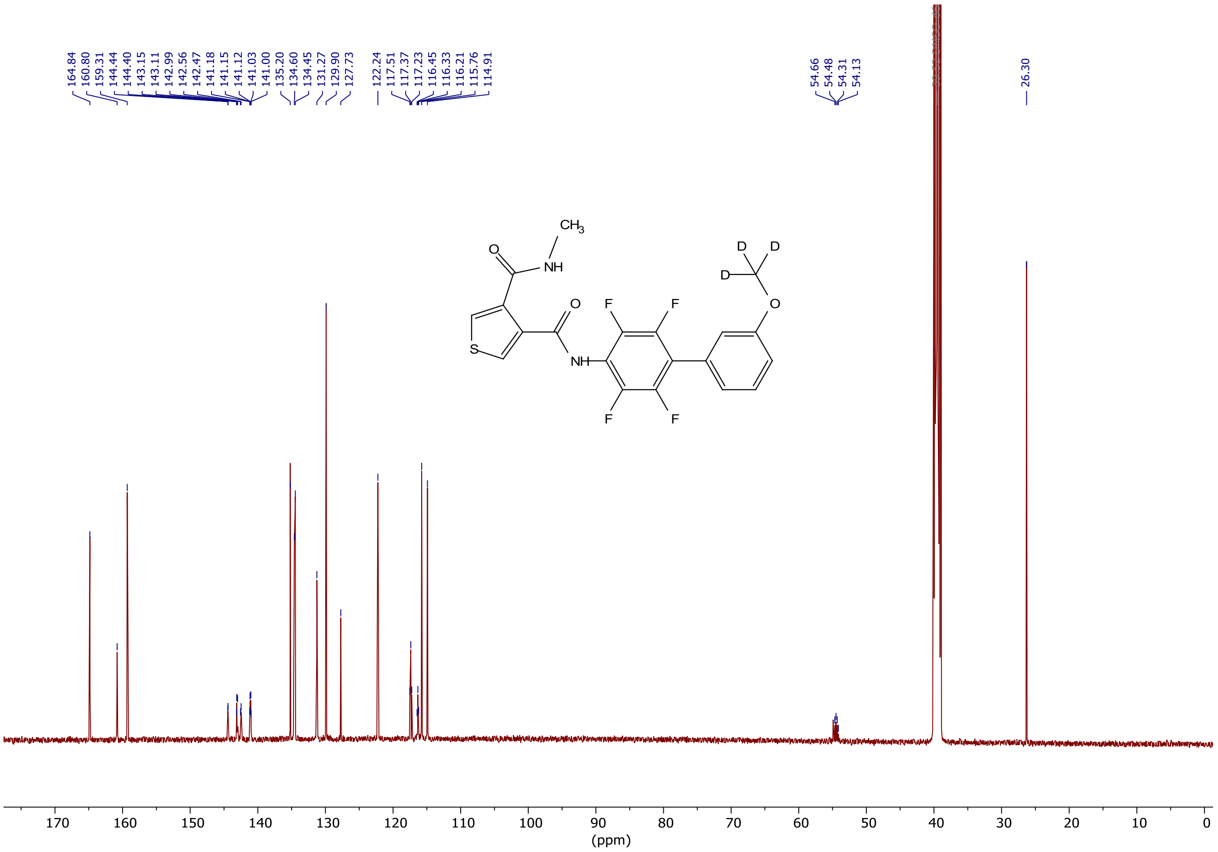

^{13}C-NMR (126 MHz, DMSO-d_6) of compound **29**

Peak ID	Compound	Time	Mass Found
31	Found	4.22	464.08,442.08

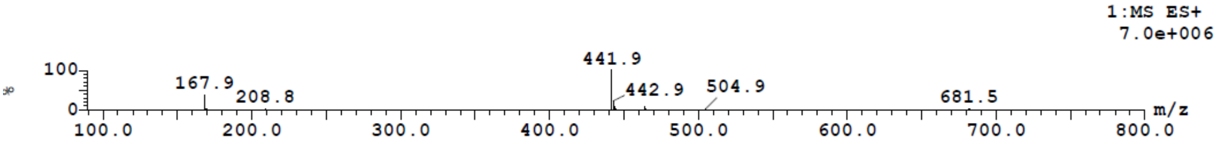

MS of compound **29**

Peak Number	Compound	Time	AreaAbs	Area %Total	Width	Height	Mass Found
31	Found	4.21	1e+005	100.00	0	3e+006	441.08, 441.08

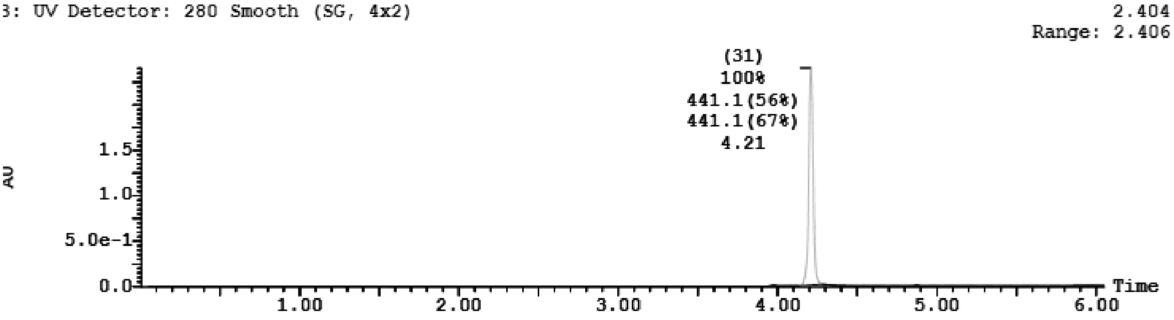

Peak Number	Compound	Time	AreaAbs	Area %Total	Width	Height	Mass Found
31	Found	4.21	8e+004	100.00	0	2e+006	441.08, 441.08

Chromatographic purity analysis of compound **29**

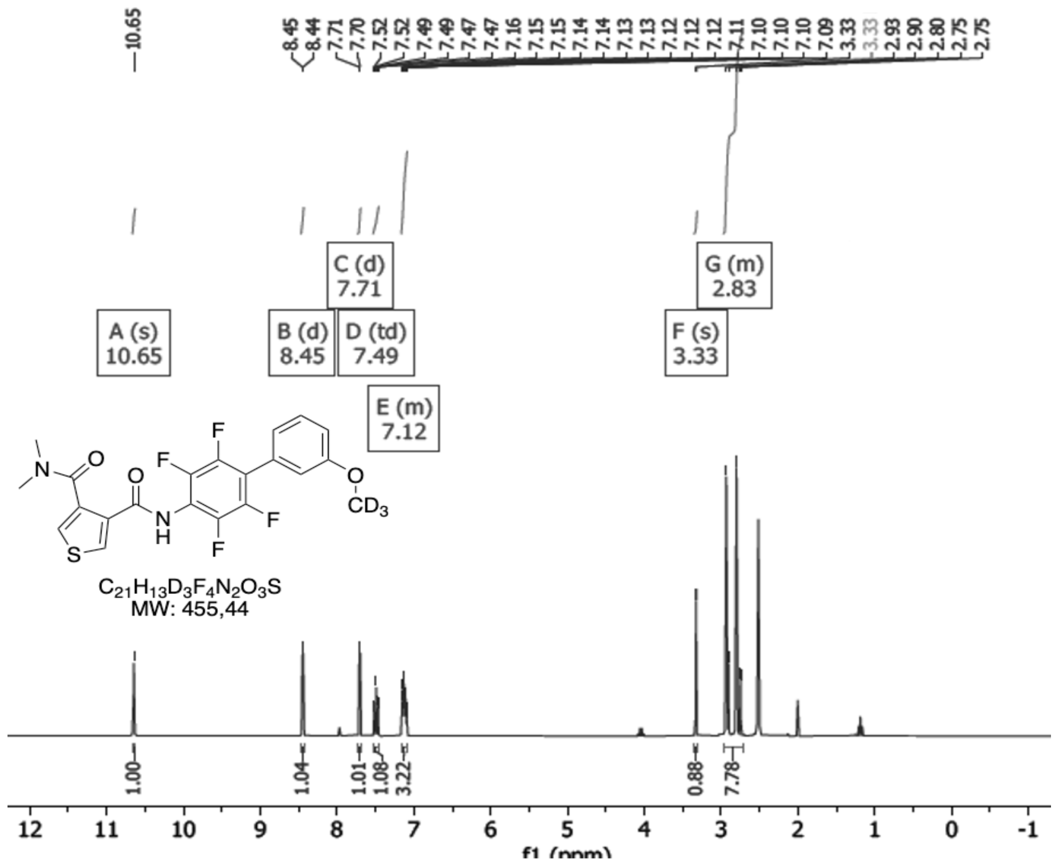

^{1}H-NMR (300 MHz, DMSO-d_6) of compound **30**

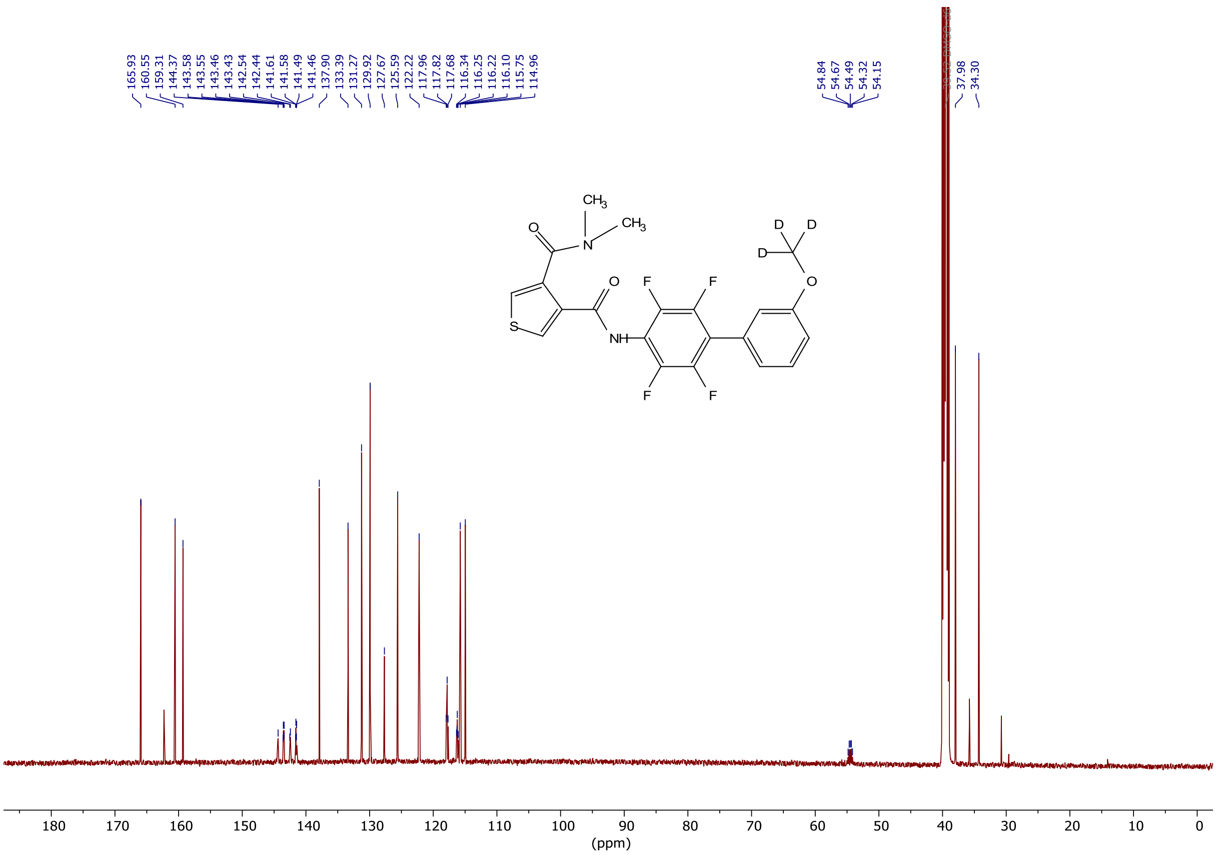

^{13}C-NMR (126 MHz, DMSO-d_6) of compound **30**

Peak ID	Compound	Time	Mass Found
31	Found	4.02	,479.08,457.08

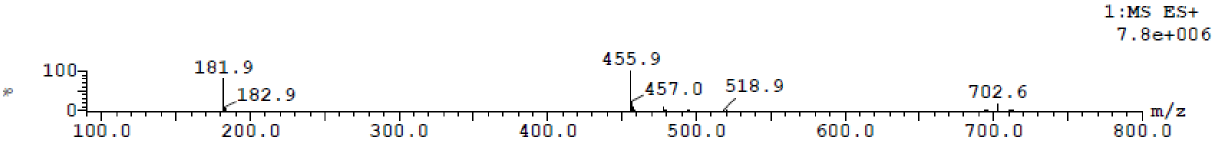

MS of compound **30**

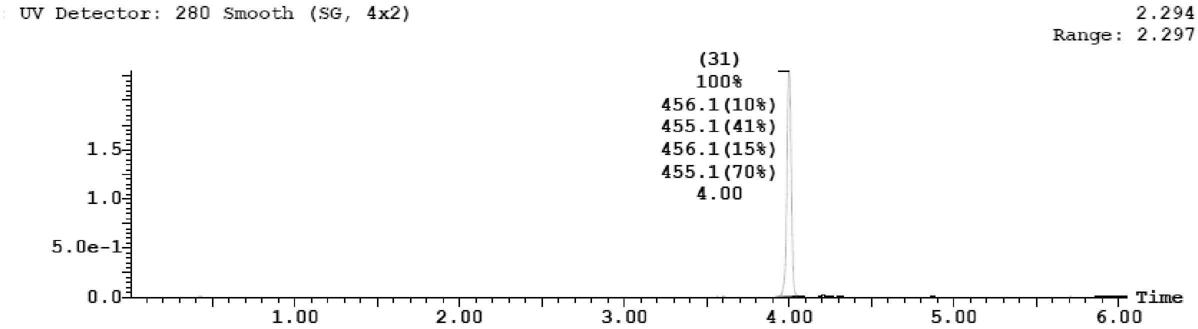

Peak Number	Compound	Time	AreaAbs	Area %Total	Width	Height	Mass Found
31	Found	4.00	7e+004	100.00	0	2e+006	455.10, 456.08

Chromatographic purity analysis of compound **30**

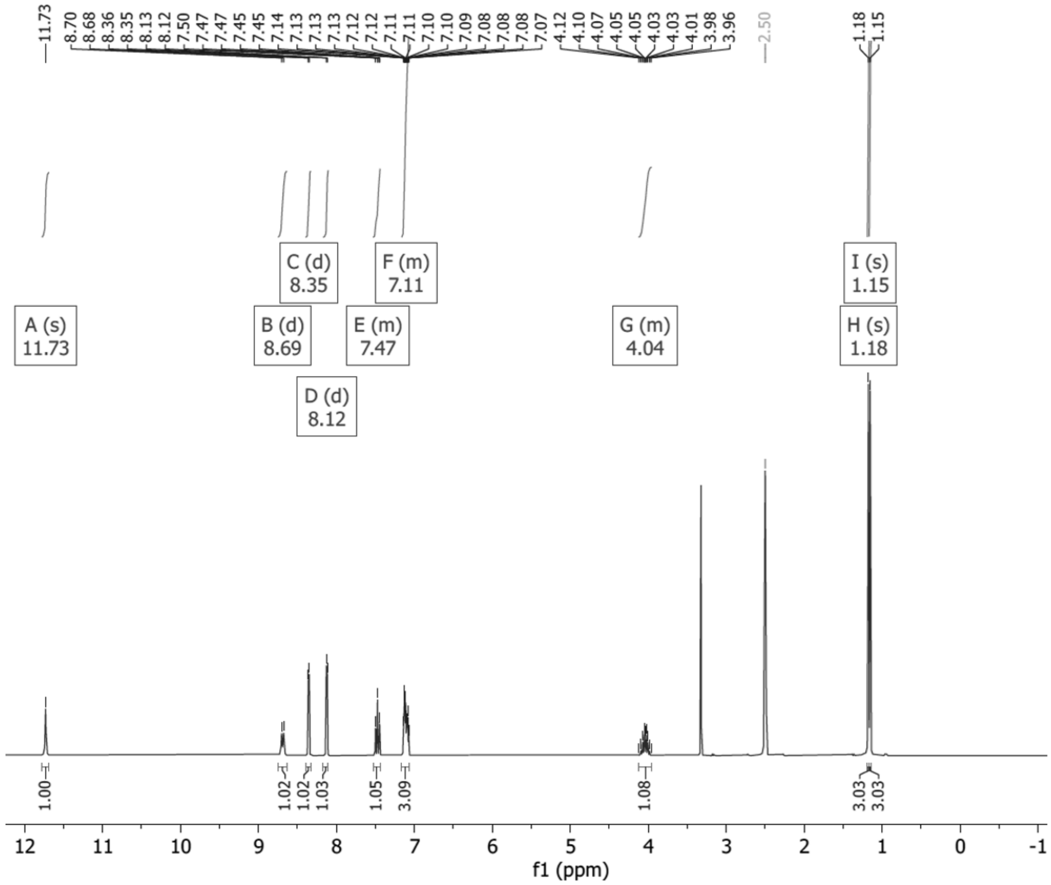

^{1}H-NMR (300 MHz, DMSO-d_6) of compound **31**

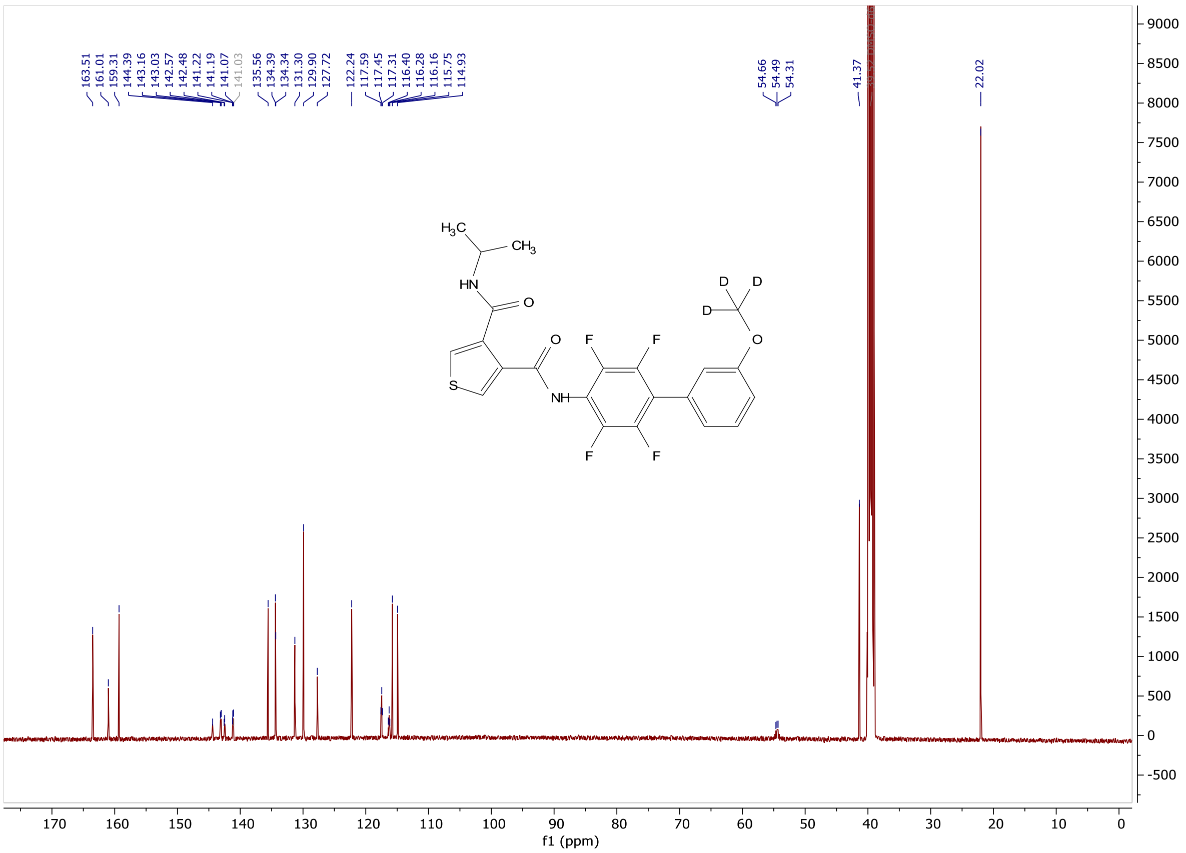

^{13}C-NMR (126 MHz, DMSO-d_6) of compound **31**

Peak ID	Compound	Time	Mass Found
6	Found	4.58	492.10,470.10

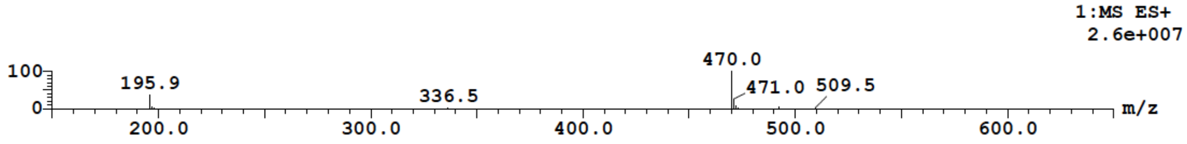

MS of compound **31**

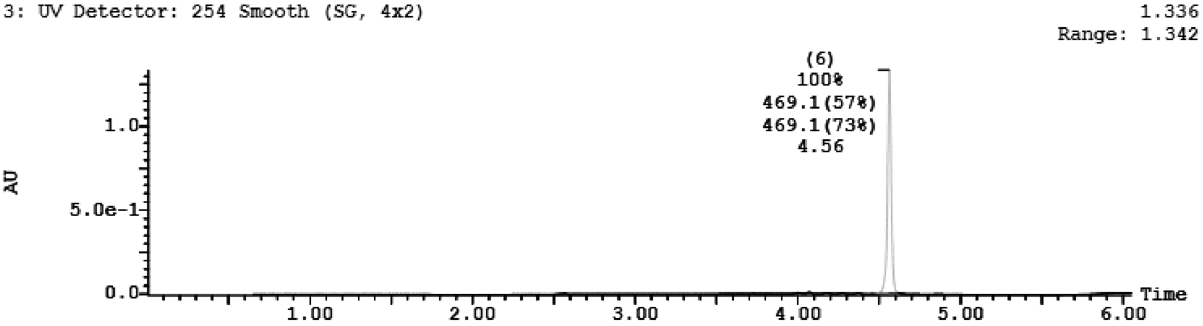

Peak Number	Compound	Time	AreaAbs	Area %Total	Width	Height	Mass Found
6	Found	4.56	4e+004	100.00	0	1e+006	469.10, 469.10

Chromatographic purity analysis of compound **31**

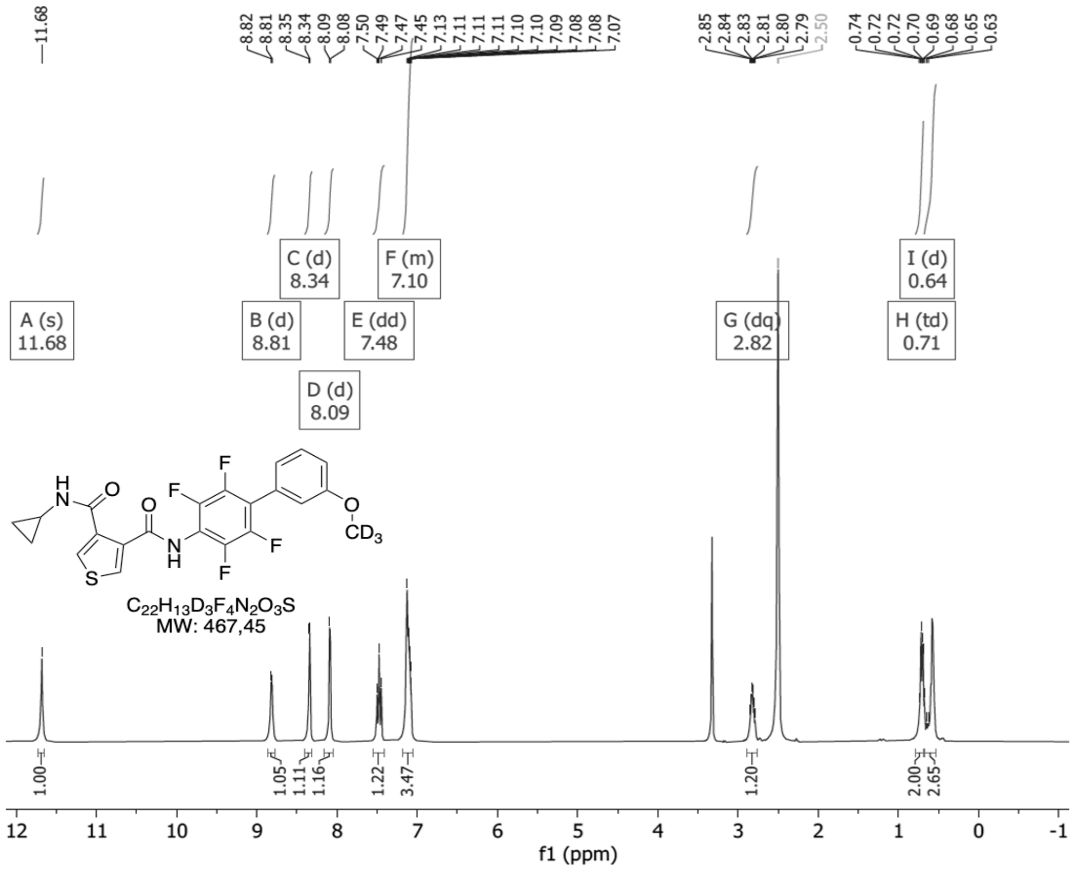

^{1}H-NMR (300 MHz, DMSO-*d*6) of compound **32**

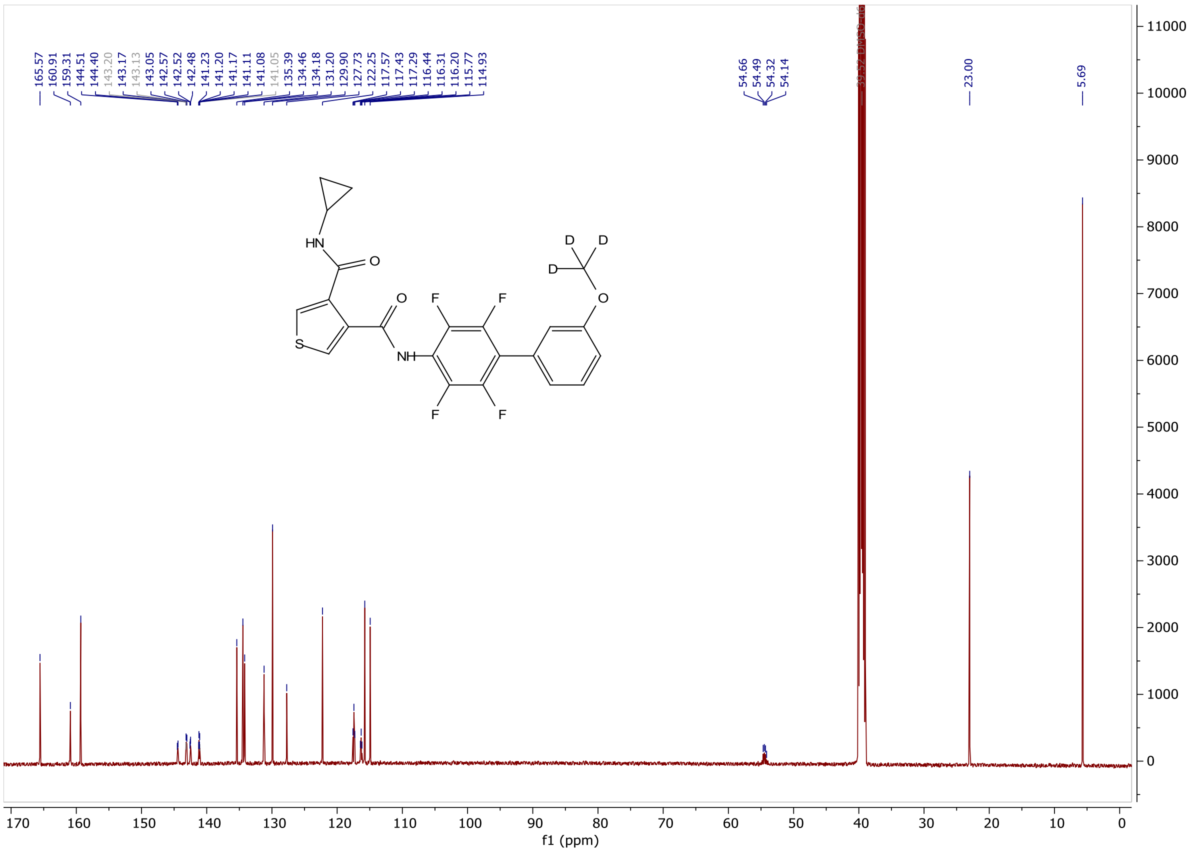

^{13}C-NMR (126 MHz, DMSO-*d6*) of compound **32**

Peak ID	Compound	Time	Mass Found
3	Found	4.38	490.10,468.10

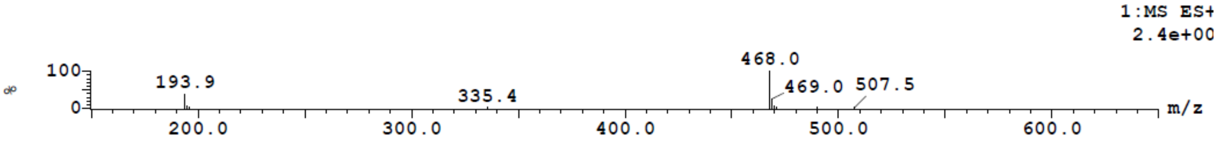

MS of compound **32**

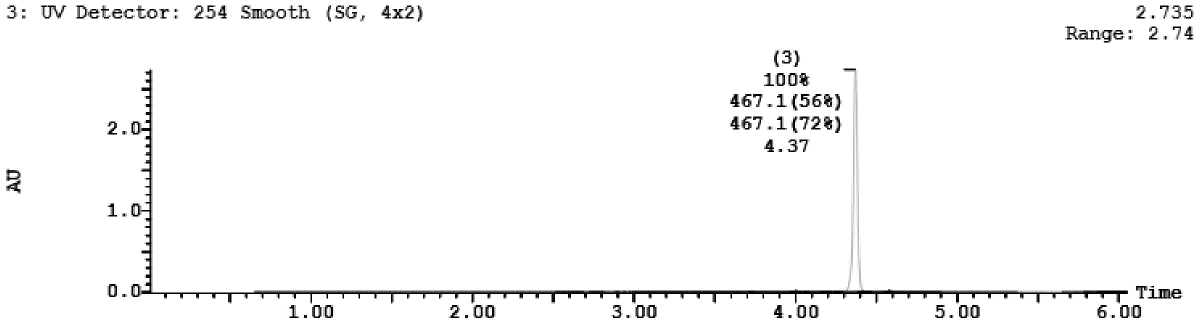

Peak Number	Compound	Time	AreaAbs	Area %Total	Width	Height	Mass Found
3	Found	4.37	8e+004	100.00	0	3e+006	467.10, 467.10

Chromatographic purity analysis of compound **32**

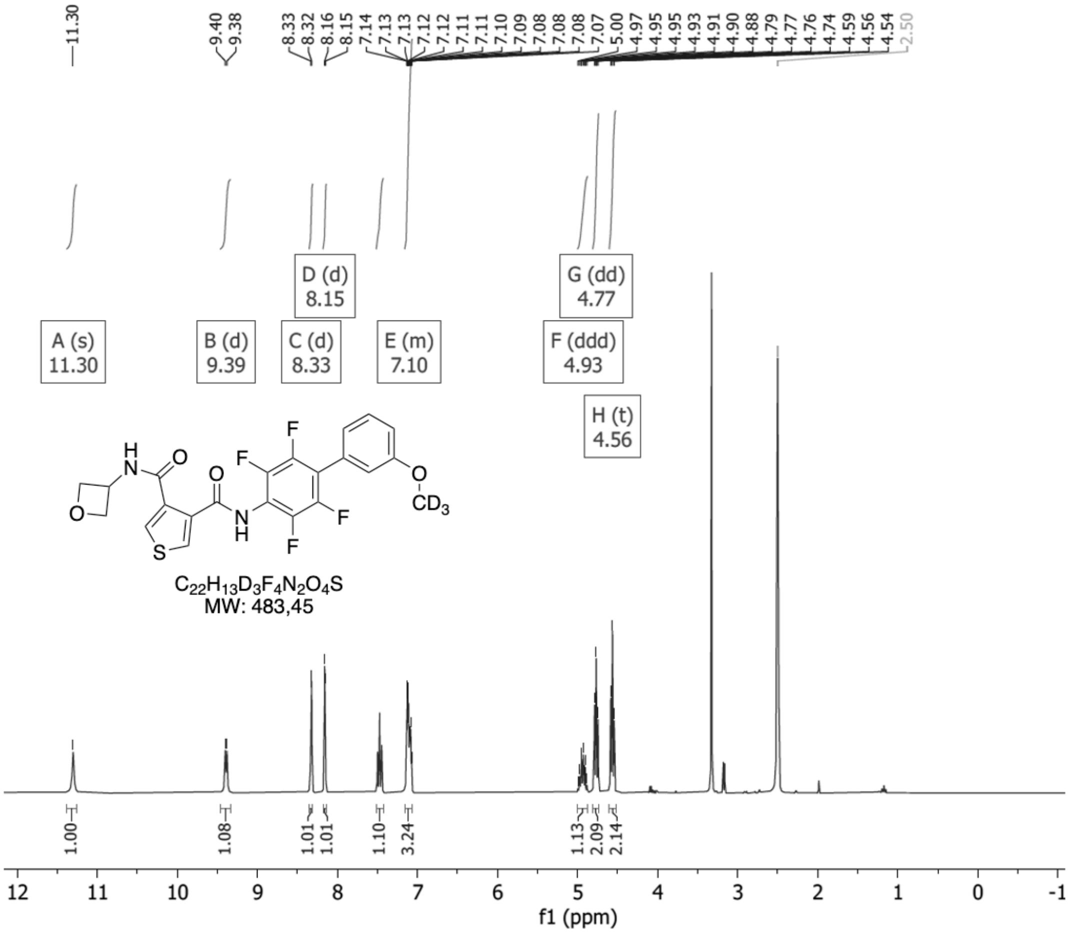

^{1}H-NMR (300 MHz, DMSO-*d*6) of compound **33**

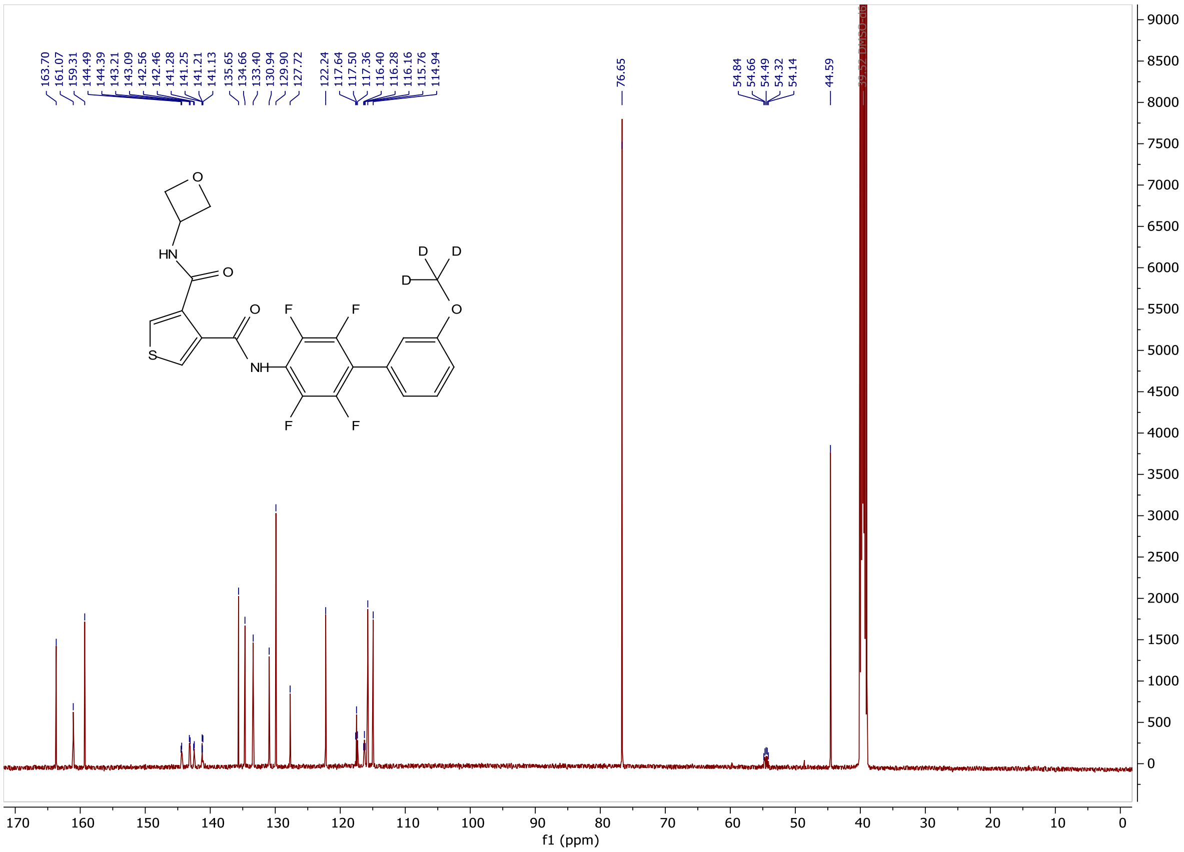

^{13}C-NMR (126 MHz, DMSO-d_6) of compound **33**

Peak ID	Compound	Time	Mass Found
5	Found	4.09	506.10,484.10

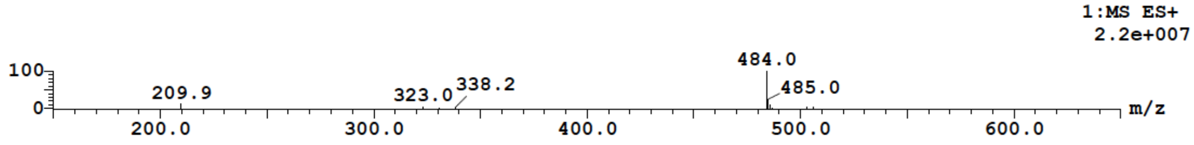

MS of compound **33**

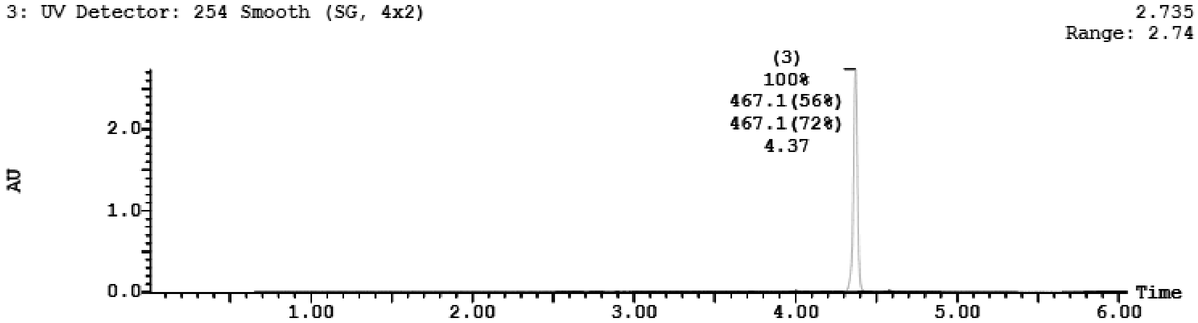

Peak Number	Compound	Time	AreaAbs	Area %Total	Width	Height	Mass Found
3	Found	4.37	8e+004	100.00	0	3e+006	467.10, 467.10

Chromatographic purity analysis of compound **33**

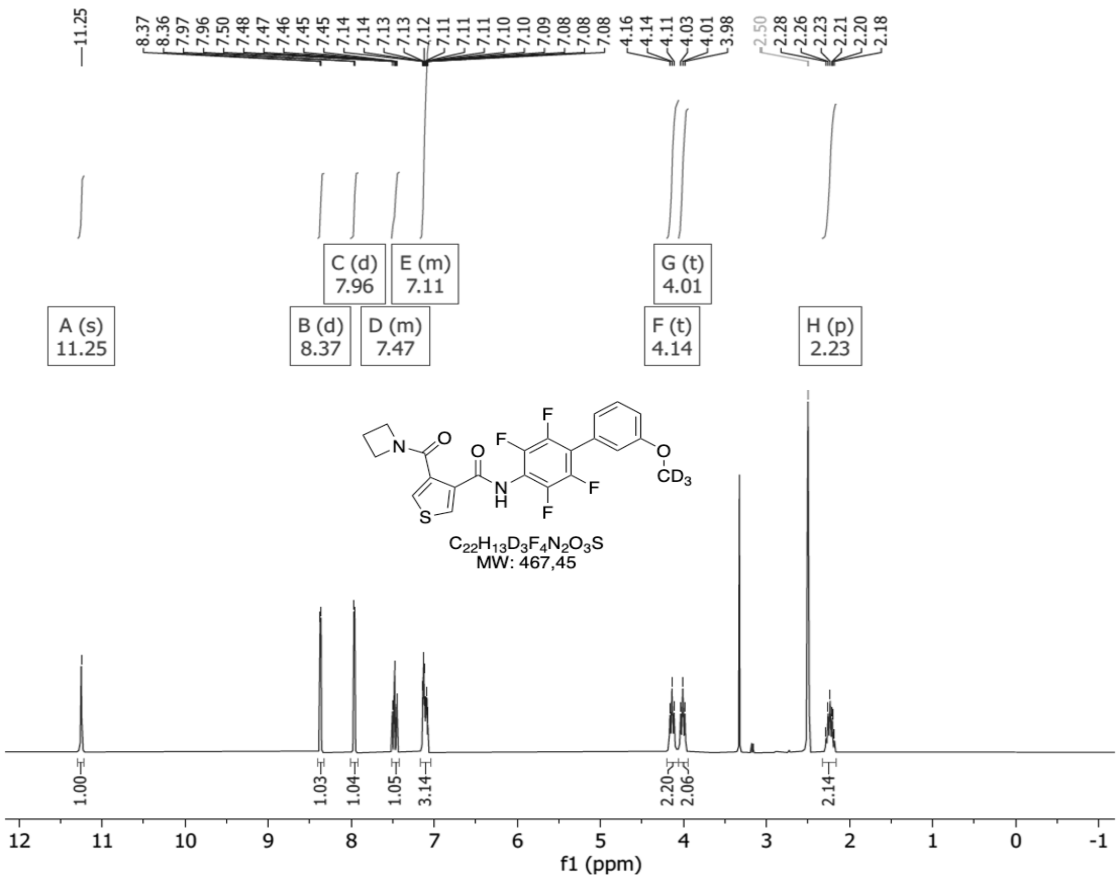

^{1}H-NMR (300 MHz, DMSO-d_6) of compound **34**

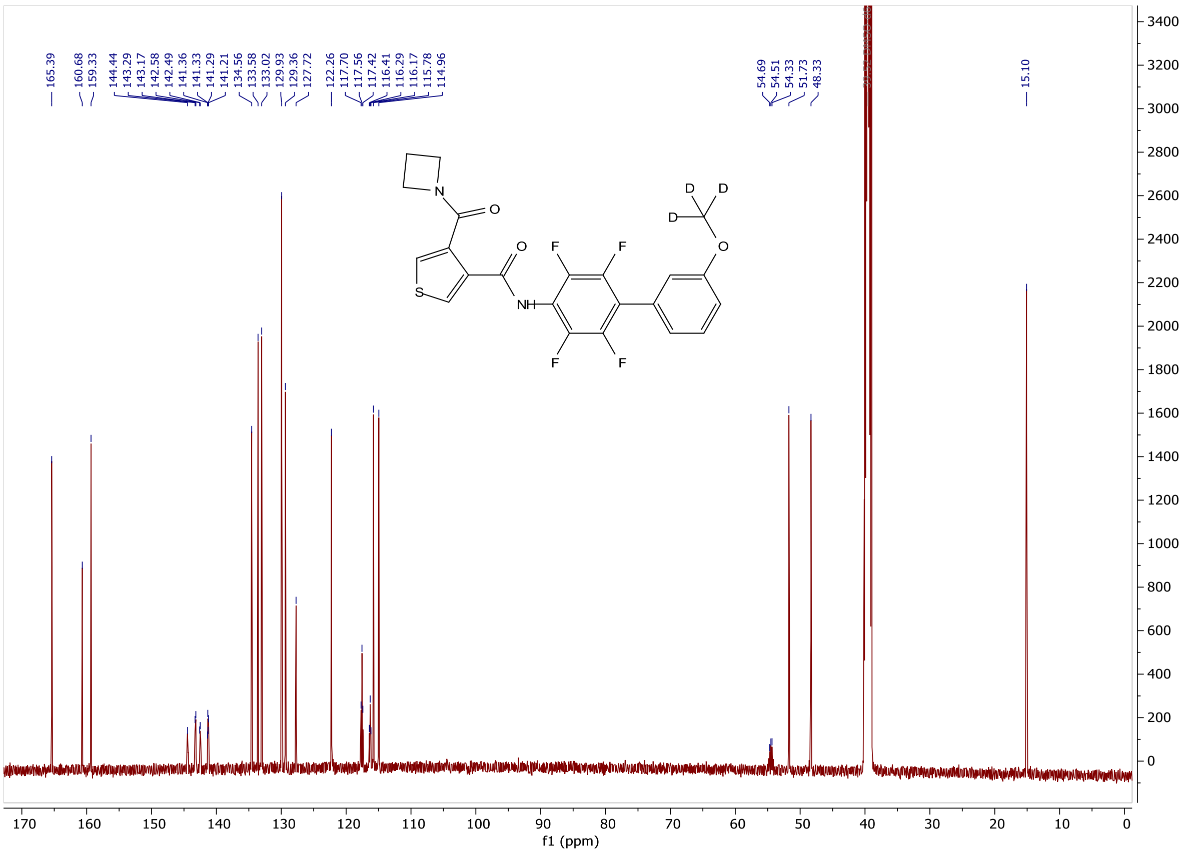

^{13}C-NMR (126 MHz, DMSO-d_6) of compound **34**

Peak ID	Compound	Time	Mass Found
6	Found	4.26	490.10,468.10

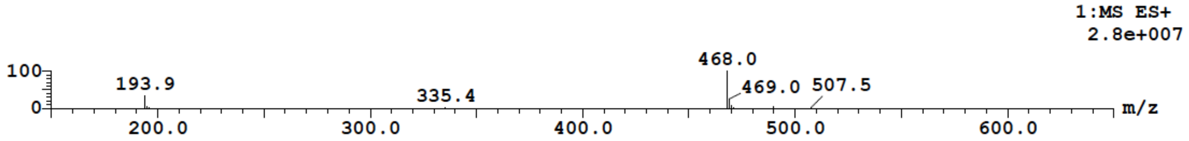

MS of compound **34**

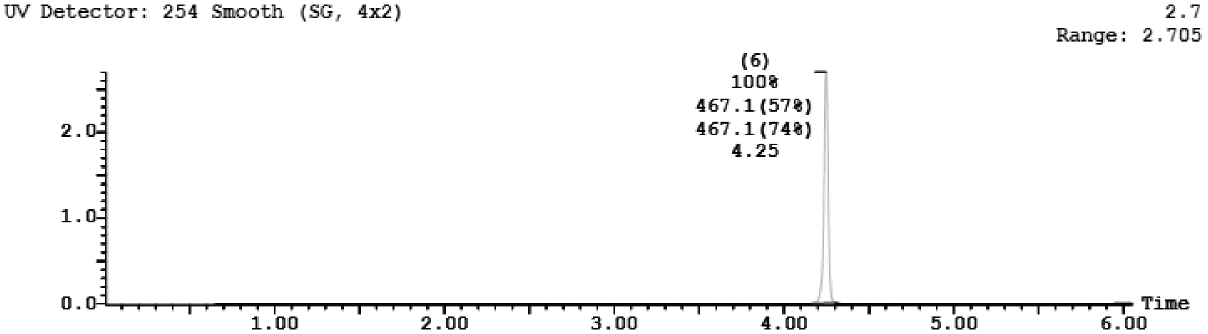

eak Number	Compound	Time	AreaAbs	Area %Total	Width	Height	Mass Found
6	Found	4.25	8e+004	100.00	0	3e+006	467.10, 467.10

Chromatographic purity analysis of compound **34**

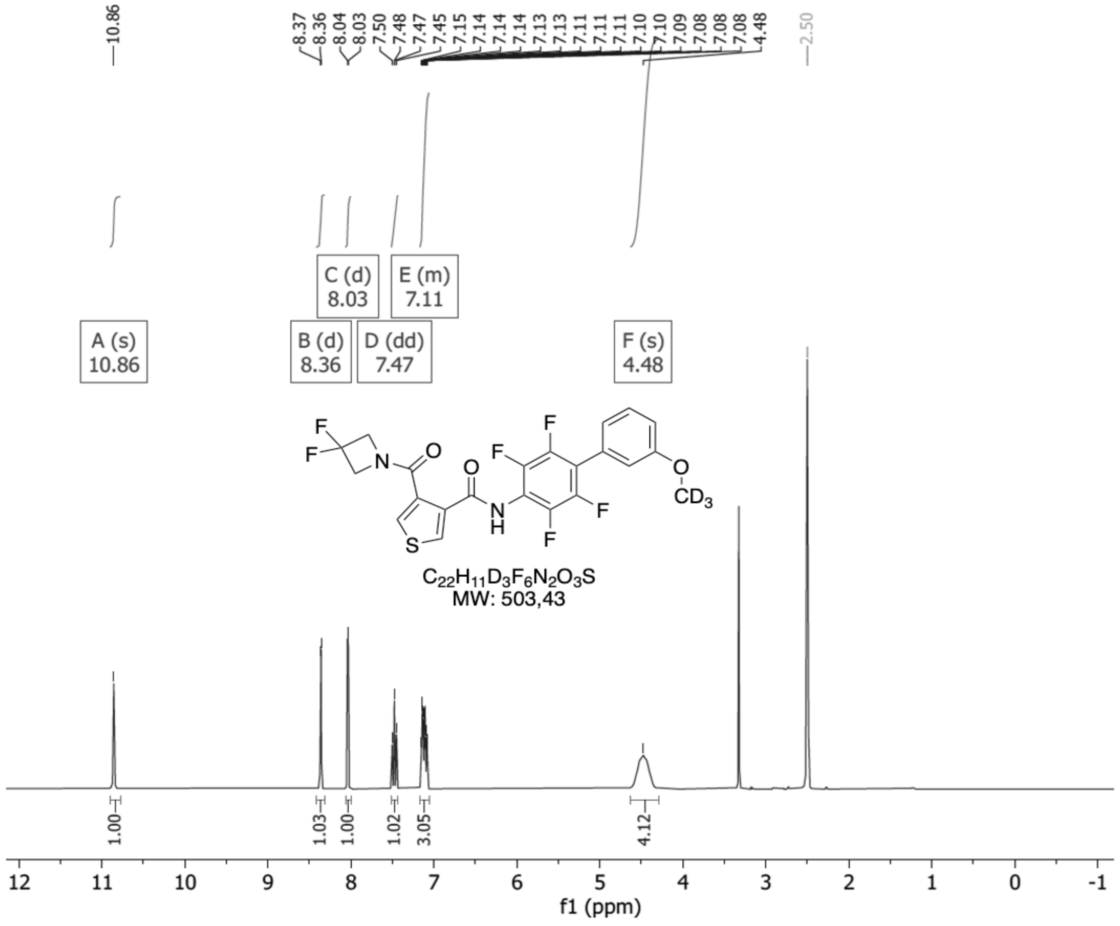

^{1}H-NMR (300 MHz, DMSO-d_6) of compound **35**

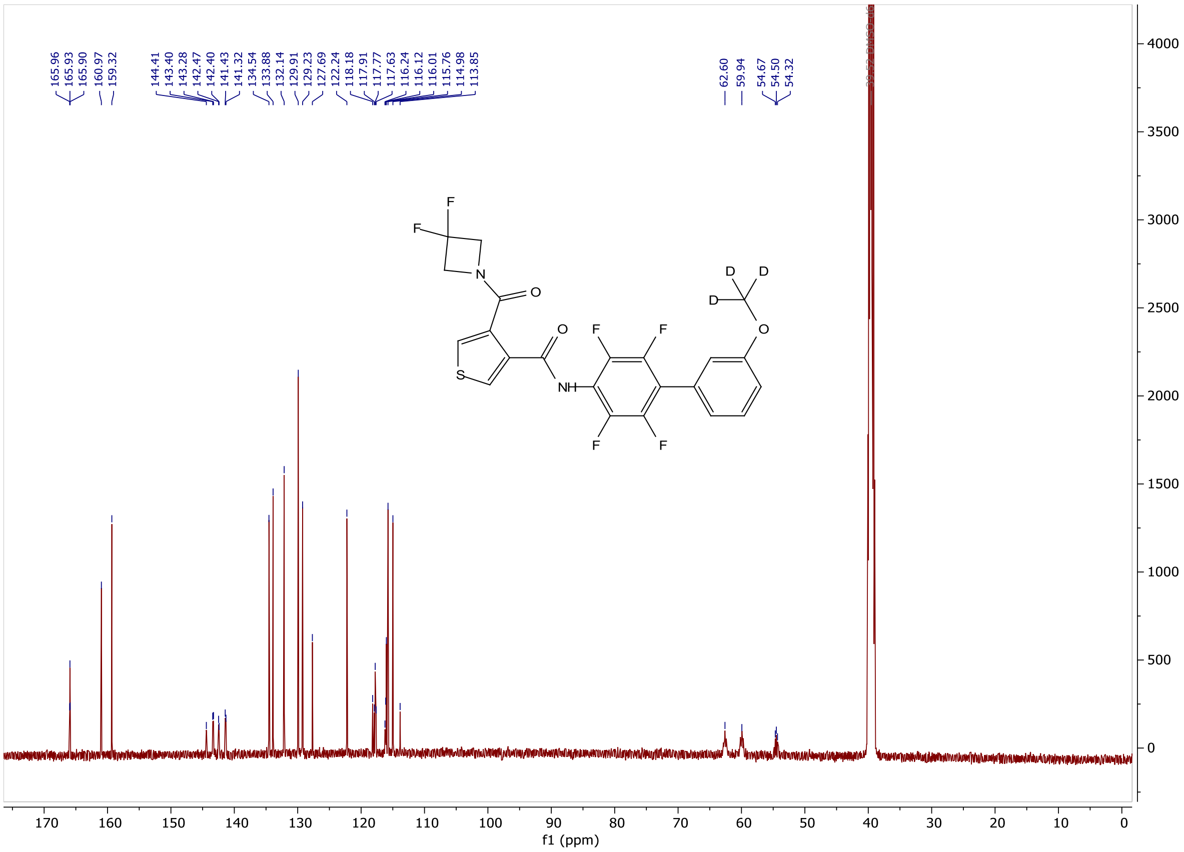

^{13}C-NMR (126 MHz, DMSO-d_6) of compound **35**

Peak ID	Compound	Time	Mass Found
9	Found	4.37	526.10,504.10

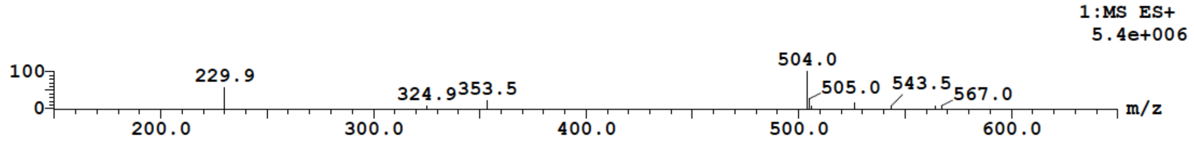

MS of compound **35**

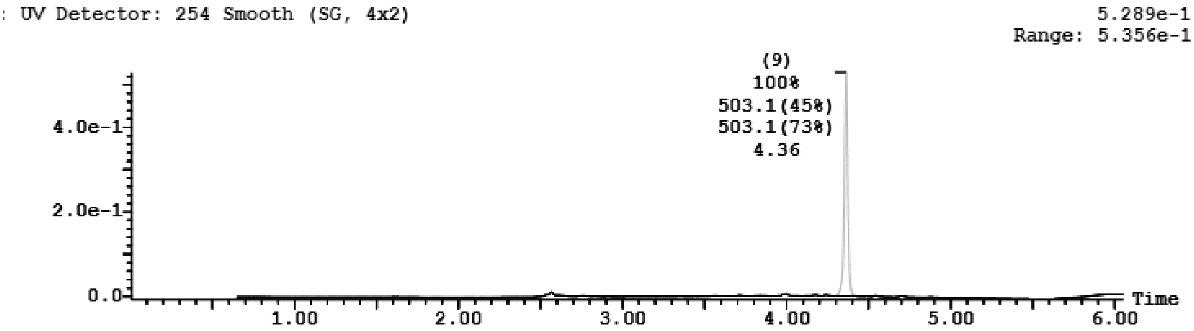

Peak Number	Compound	Time	AreaAbs	Area %Total	Width	Height	Mass Found
9	Found	4.36	1e+004	100.00	0	5e+005	503.10, 503.10

Chromatographic purity analysis of compound **35**

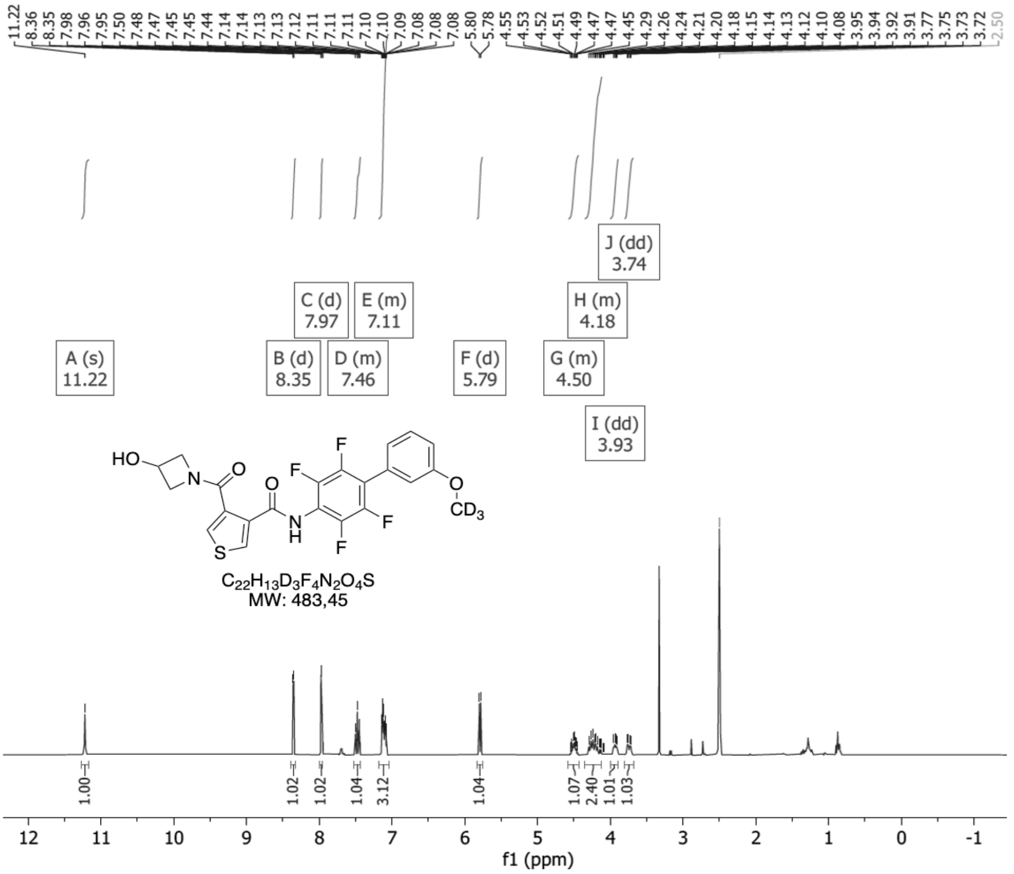

^{1}H-NMR (300 MHz, DMSO-d_6) of compound **36**

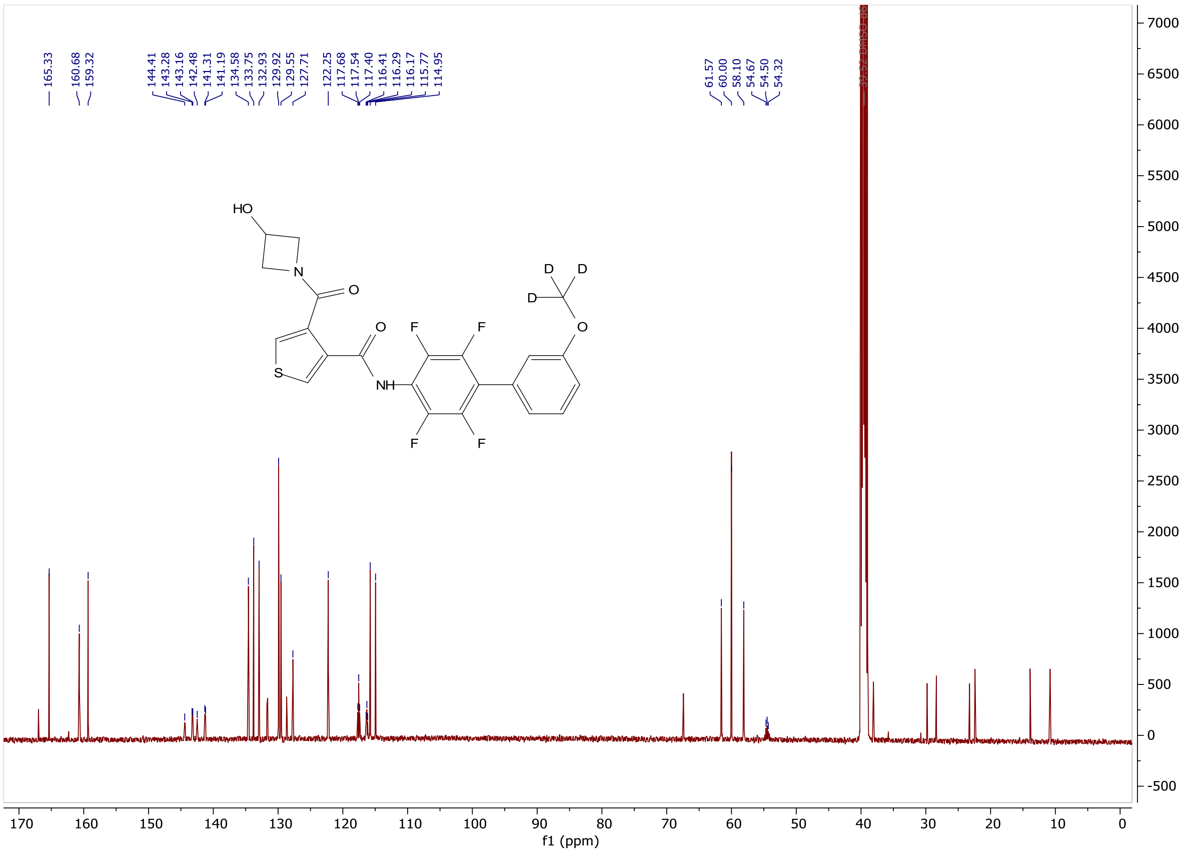

^{13}C-NMR (126 MHz, DMSO-d_6) of compound **36**

Peak ID	Compound	Time	Mass Found
4	Found	3.82	506.10,484.10

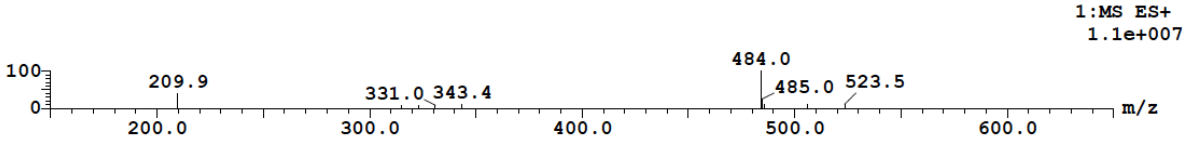

MS of compound **36**

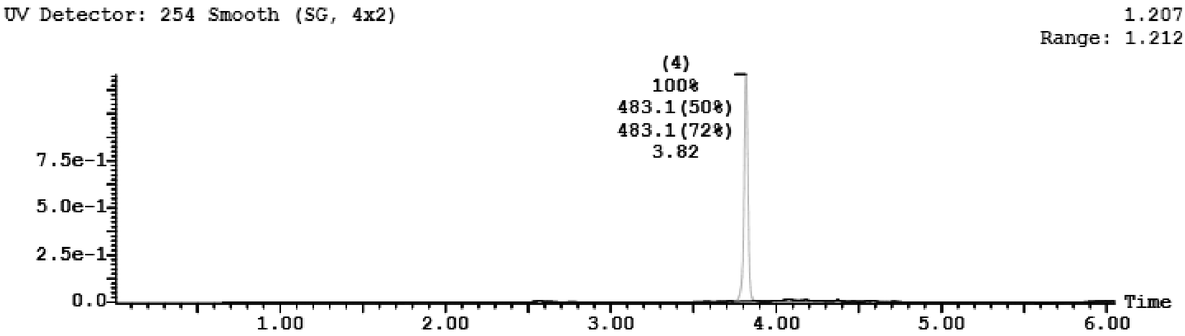

Peak Number	Compound	Time	AreaAbs	Area %Total	Width	Height	Mass Found
4	Found	3.82	3e+004	100.00	0	1e+006	483.10, 483.10

Chromatographic purity analysis of compound **36**

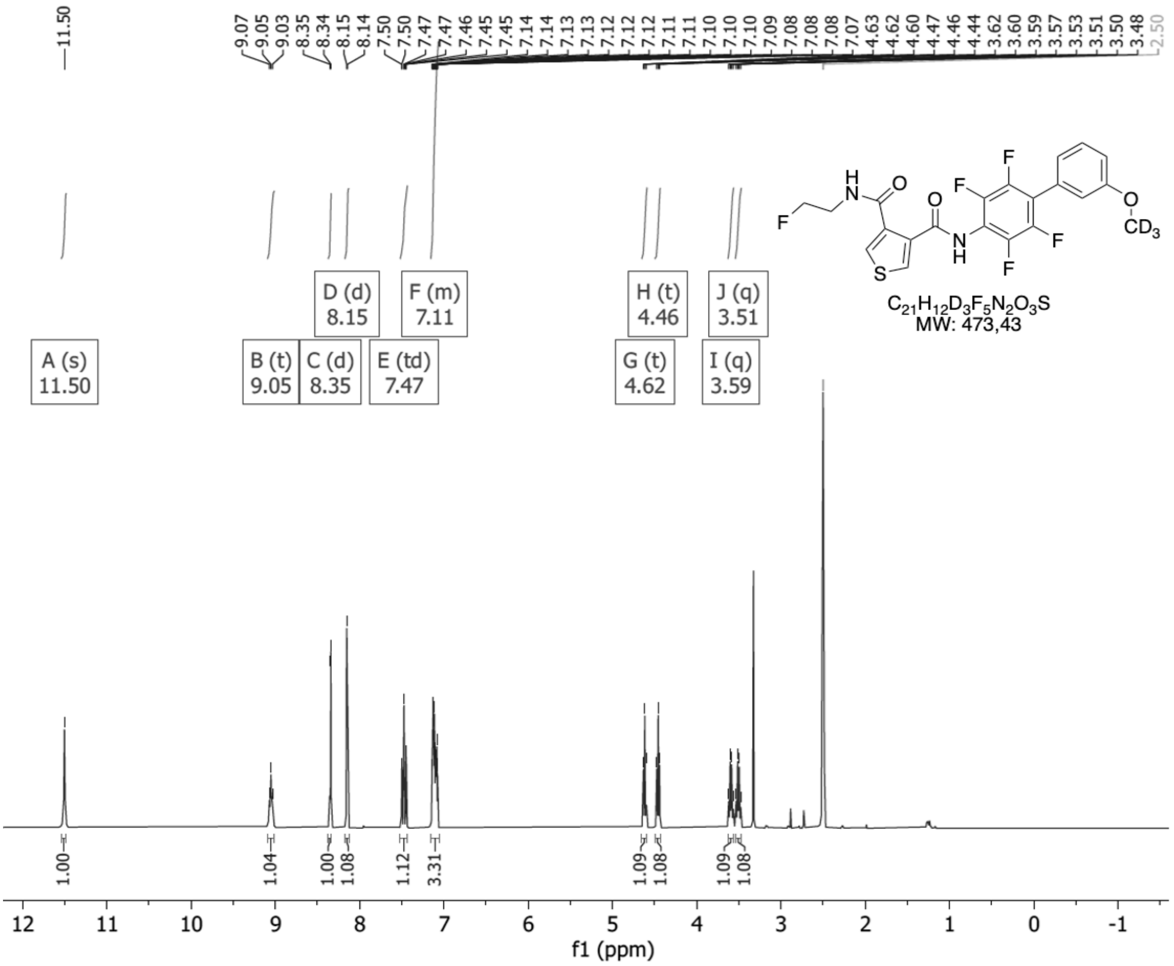

^{1}H-NMR (300 MHz, DMSO-d_6) of compound **37**

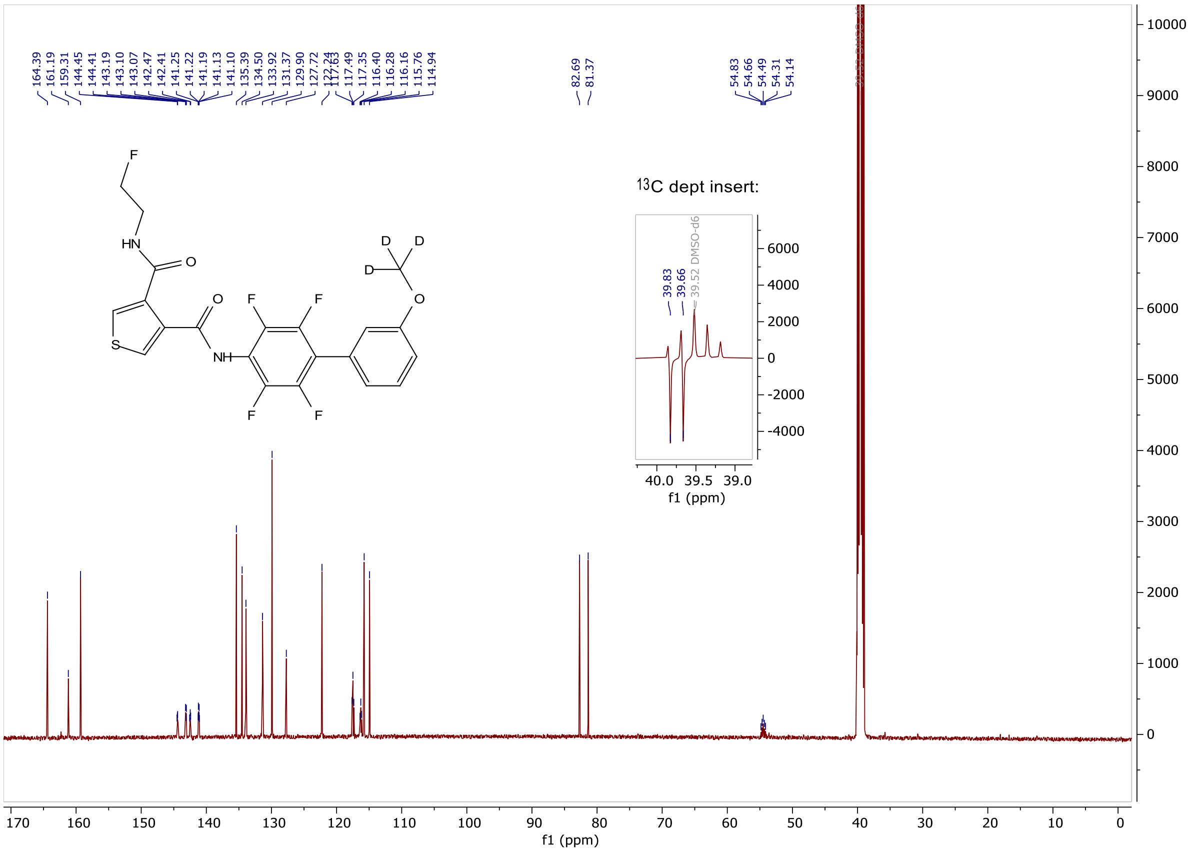

^{13}C-NMR (126 MHz, DMSO-d_6) of compound **37**

Peak ID	Compound	Time	Mass Found
2	Found	4.28	496.10,474.10

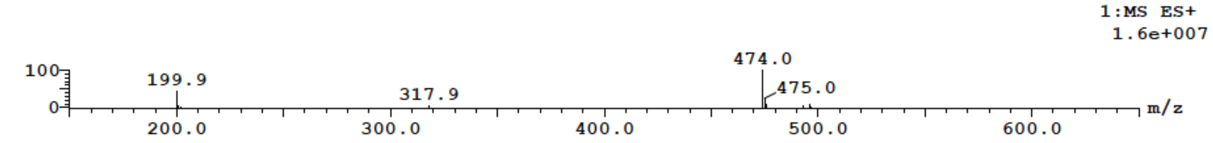

MS of compound **37**

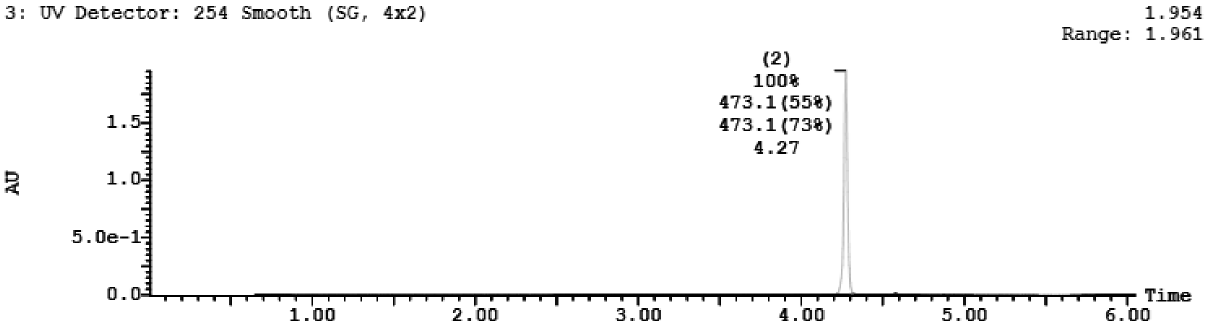

Peak Number	Compound	Time	AreaAbs	Area %Total	Width	Height	Mass Found
2	Found	4.27	5e+004	100.00	0	2e+006	473.10, 473.10

Chromatographic purity analysis of compound **37**

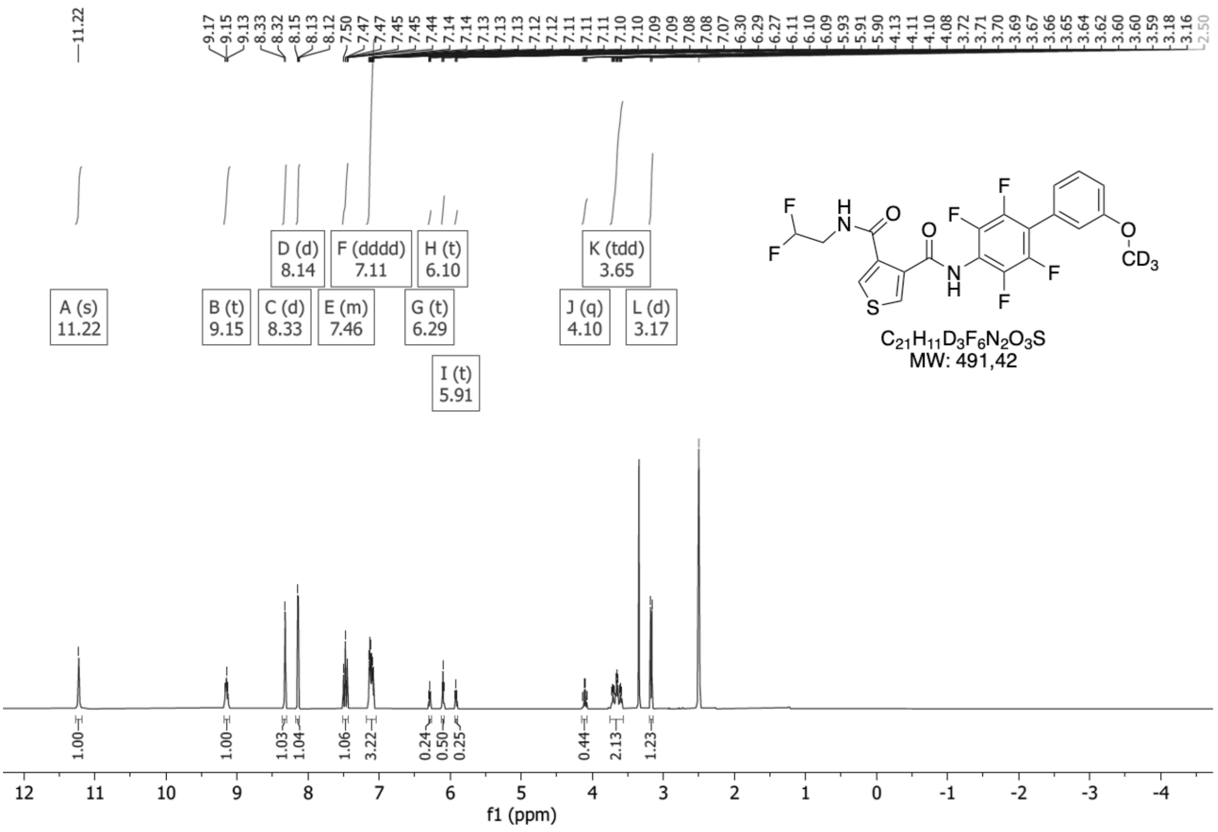

^{1}H-NMR (300 MHz, DMSO-d_6) of compound **38**

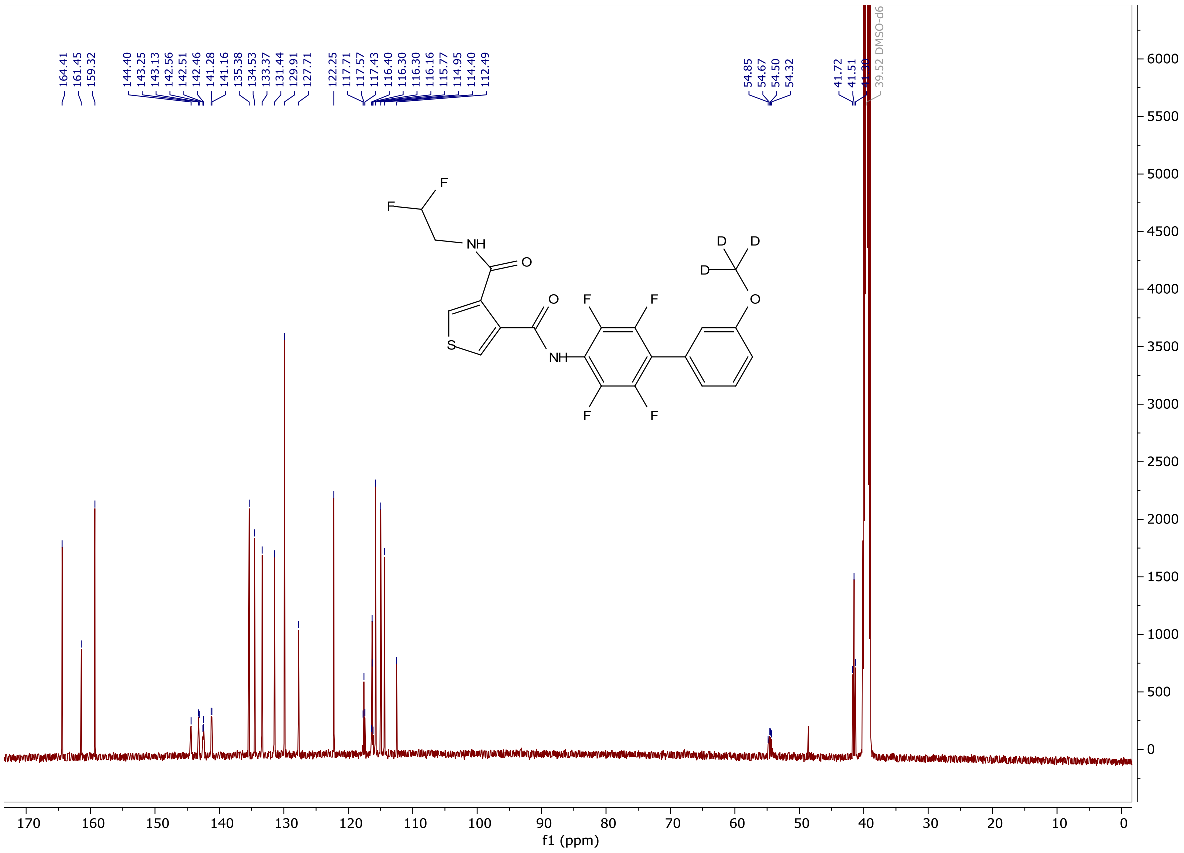

^{13}C-NMR (126 MHz, DMSO-d_6) of compound **38**

Peak ID	Compound	Time	Mass Found
11	Found	4.38	514.10,492.10

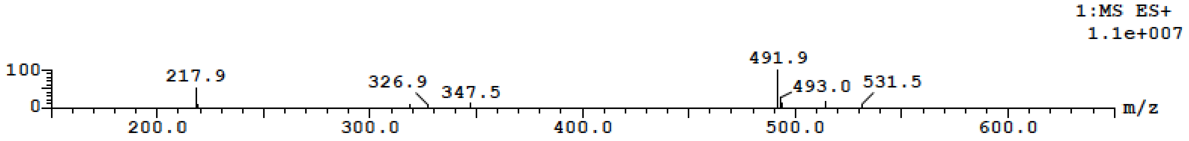

MS of compound **38**

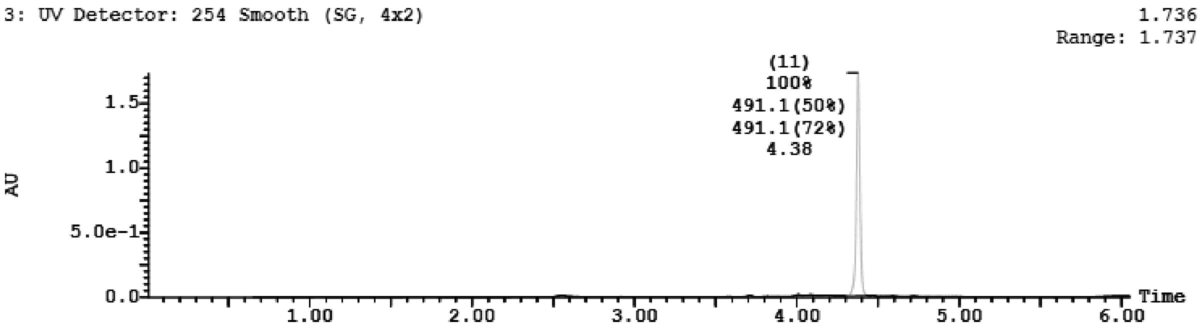

Peak Number	Compound	Time	AreaAbs	Area %Total	Width	Height	Mass Found
11	Found	4.38	5e+004	100.00	0	2e+006	491.10, 491.10

Chromatographic purity analysis of compound **38**

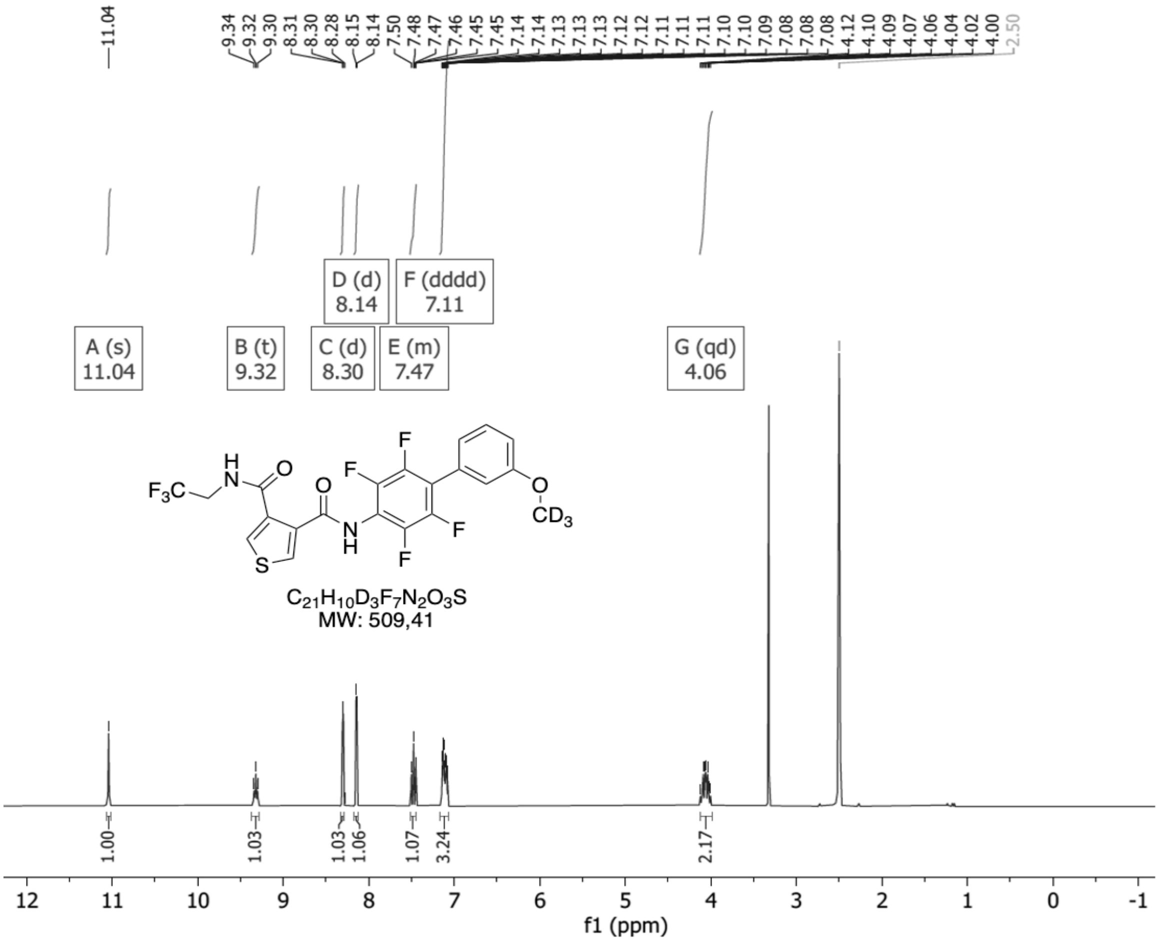

^{1}H-NMR (300 MHz, DMSO-d_6) of compound **39**

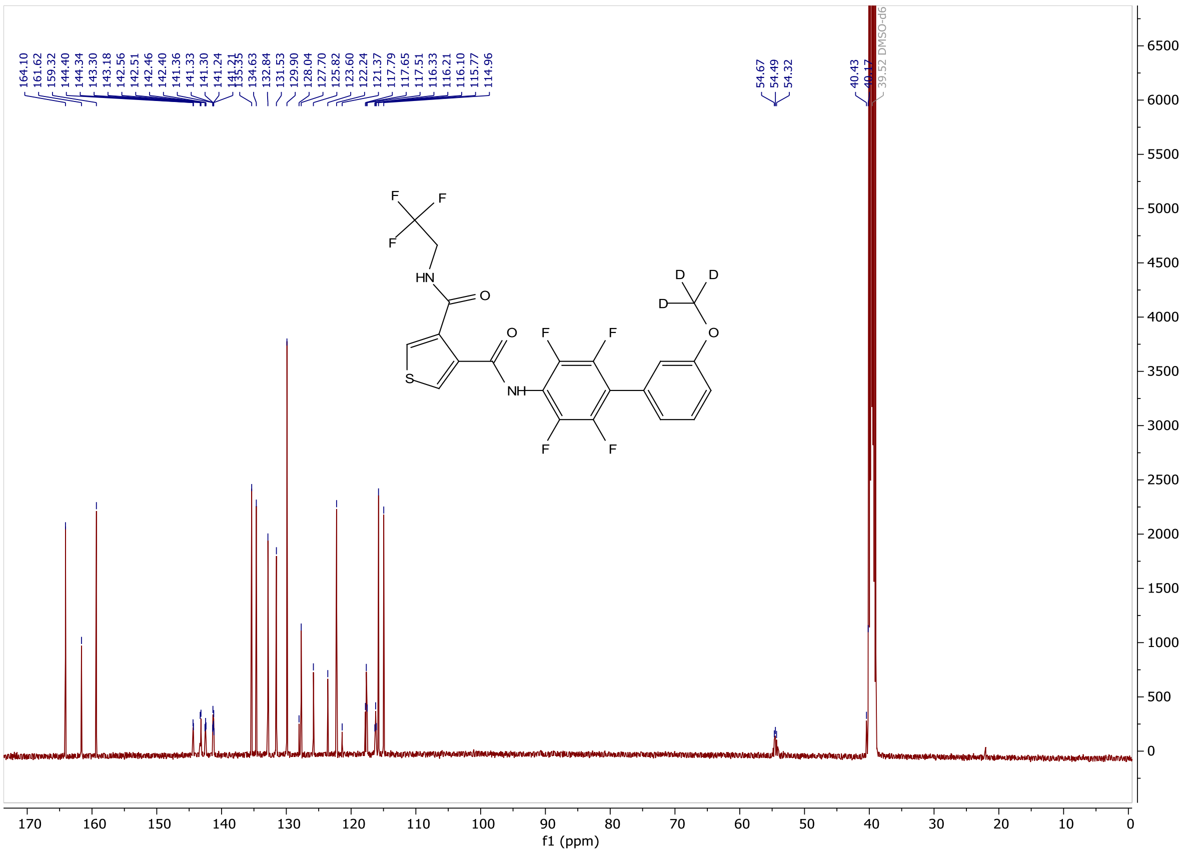

^{13}C-NMR (126 MHz, DMSO-d_6) of compound **39**

Peak ID	Compound	Time	Mass Found
4	Found	4.52	532.10,510.10

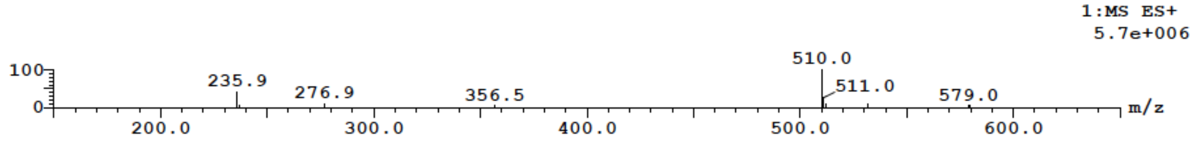

MS of compound **39**

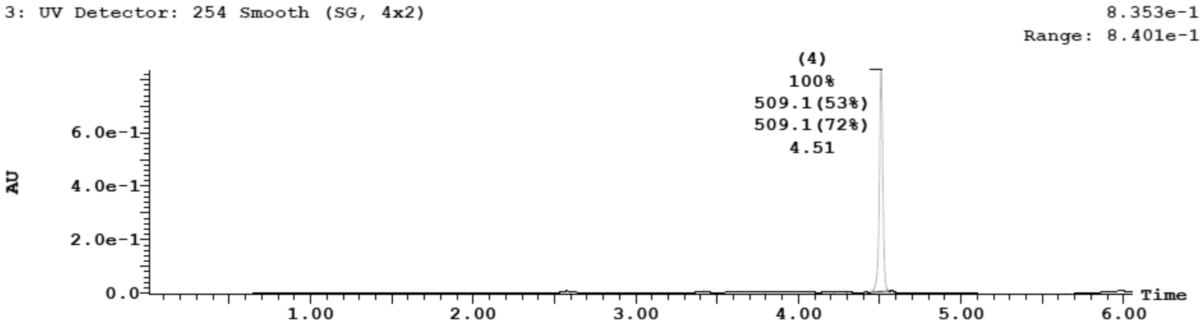

Peak Number	Compound	Time	AreaAbs	Area %Total	Width	Height	Mass Found
4	Found	4.51	2e+004	100.00	0	8e+005	509.10, 509.10

Chromatographic purity analysis of compound **39**

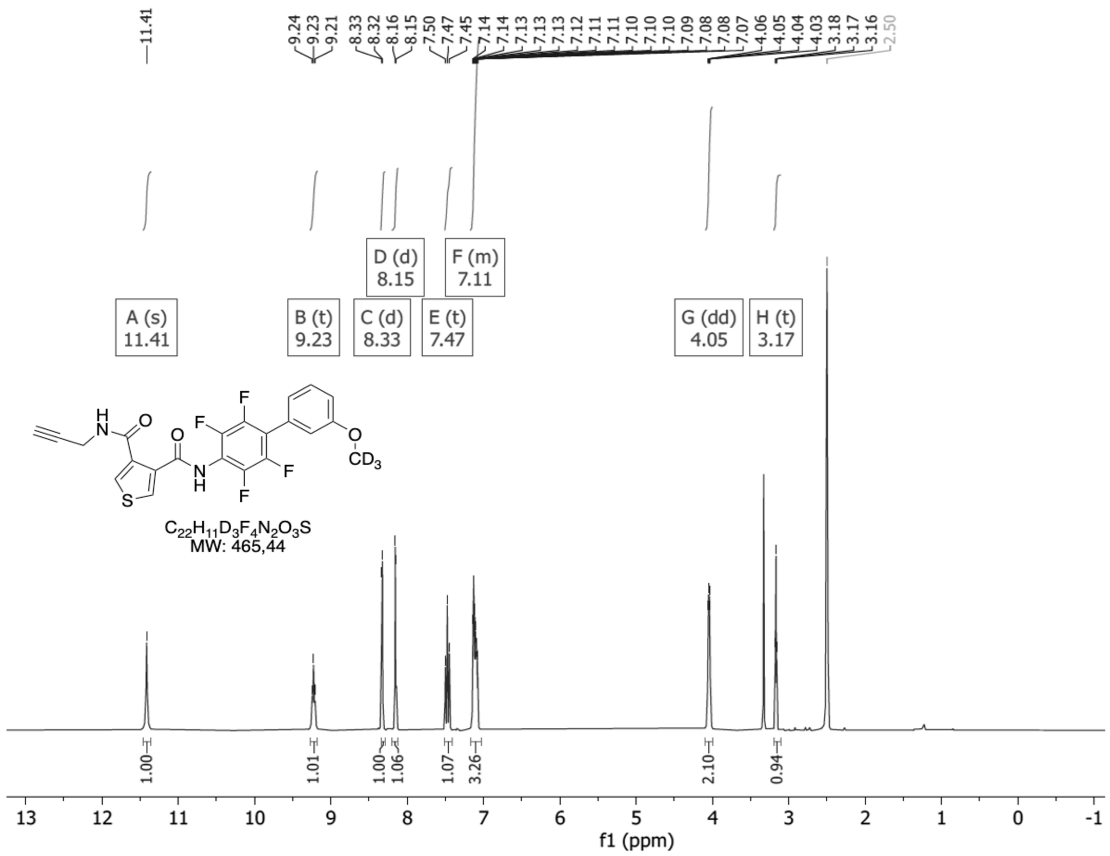

^{1}H-NMR (300 MHz, DMSO-d_6) of compound **40**

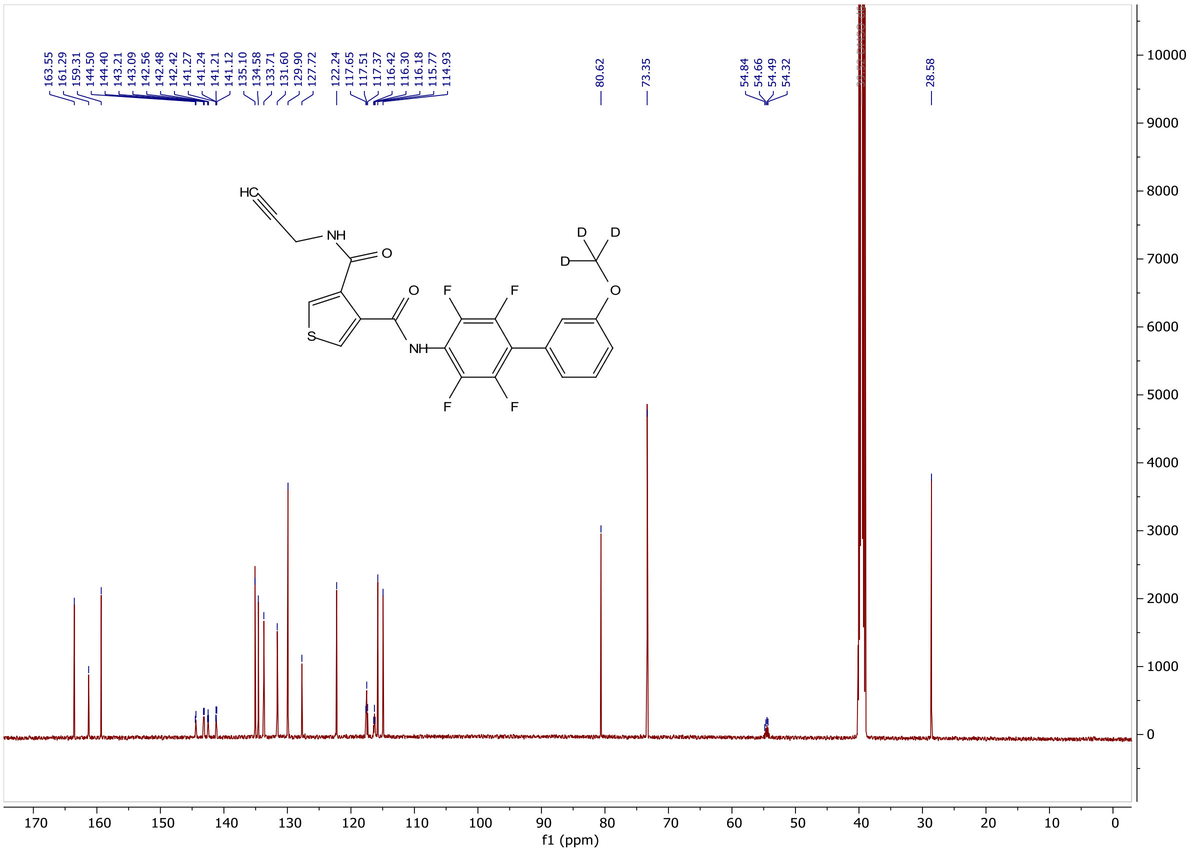

^{13}C-NMR (126 MHz, DMSO-d_6) of compound **40**

Peak ID	Compound	Time	Mass Found
5	Found	4.33	488.10,466.10

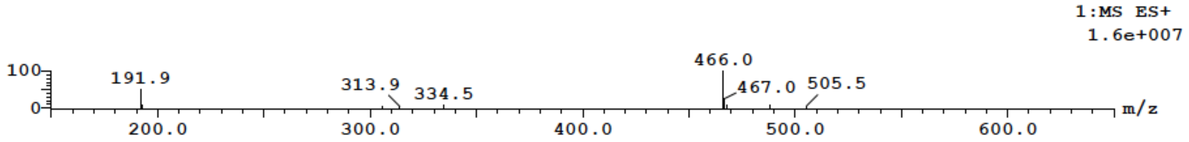

MS of compound **40**

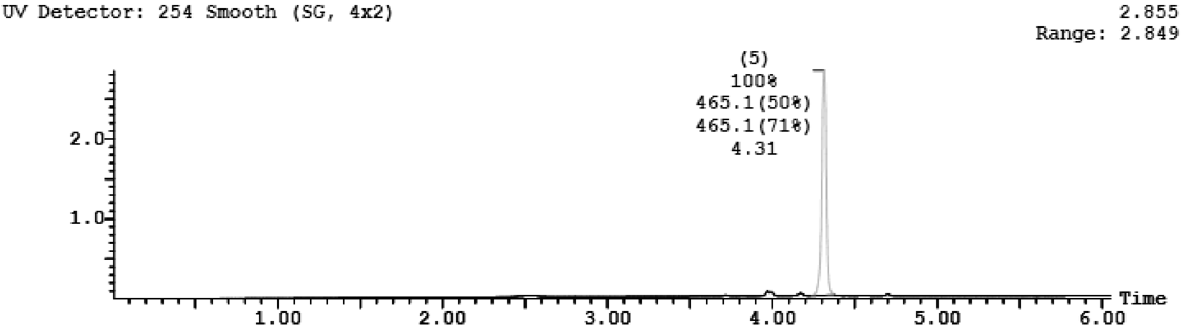

eak Number	Compound	Time	AreaAbs	Area %Total	Width	Height	Mass Found
5	Found	4.31	9e+004	100.00	0	3e+006	465.10, 465.10

Chromatographic purity analysis of compound **40**

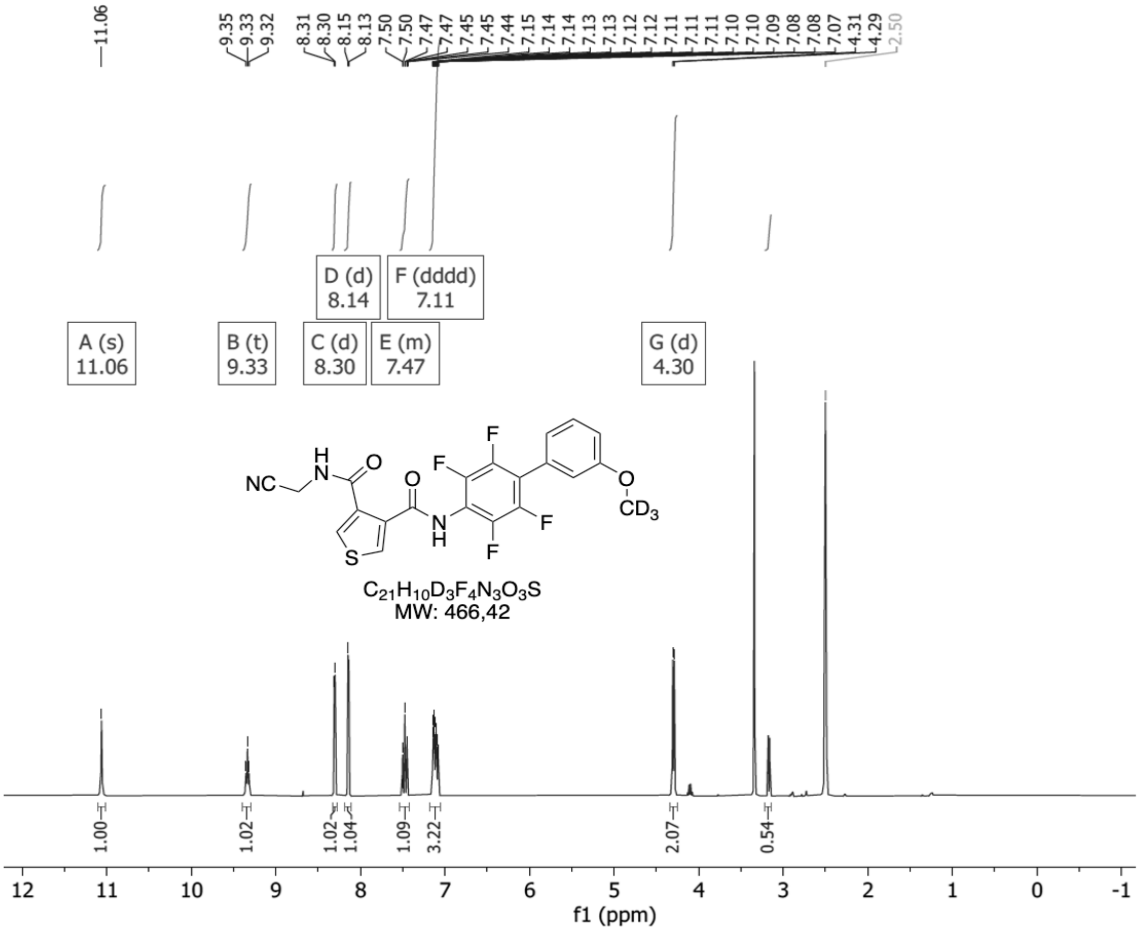

^{1}H-NMR (300 MHz, DMSO-d_6) of compound **41**

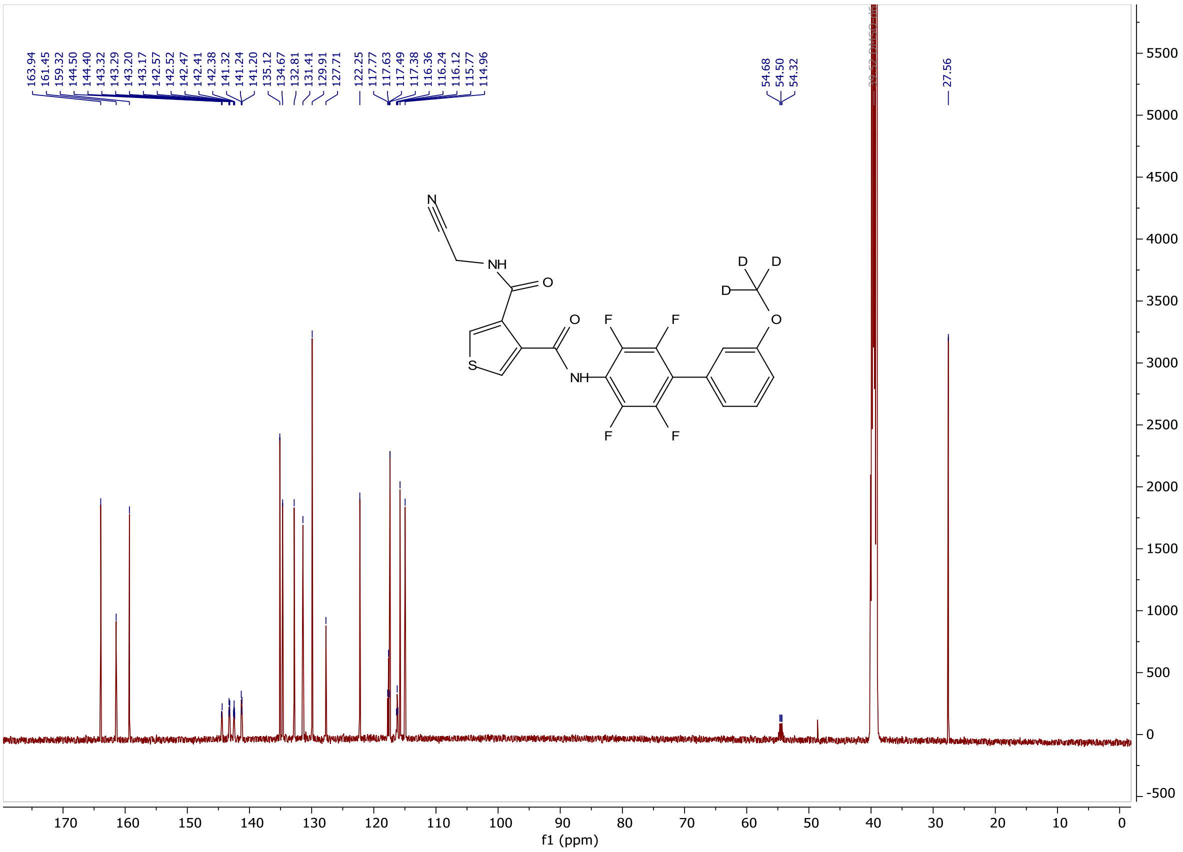

^{13}C-NMR (126 MHz, DMSO-d_6) of compound **41**

Peak ID	Compound	Time	Mass Found
3	Found	4.16	489.10,467.10

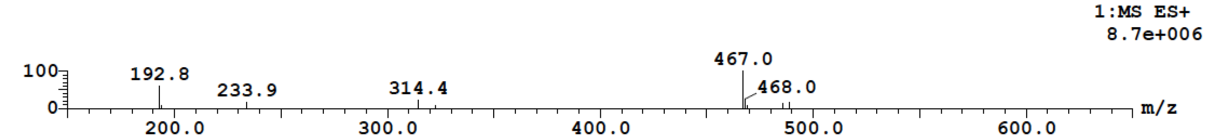

MS of compound **41**

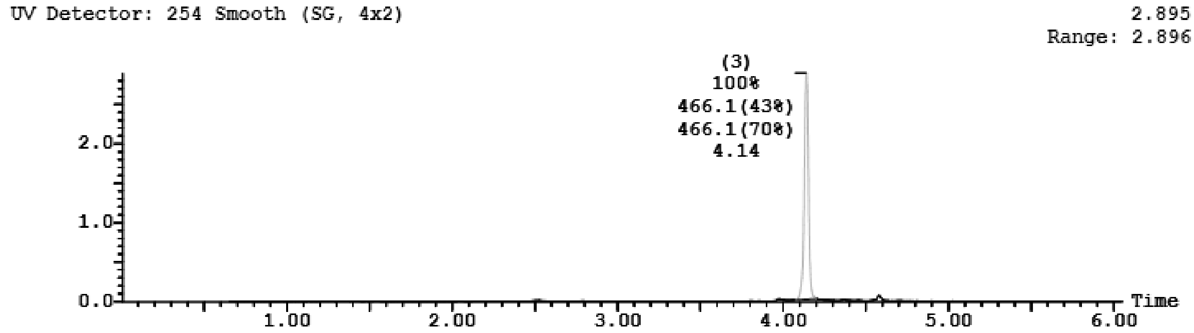

Peak Number	Compound	Time	AreaAbs	Area %Total	Width	Height	Mass Found
3	Found	4.14	9e+004	100.00	0	3e+006	466.10, 466.10

Chromatographic purity analysis of compound **41**

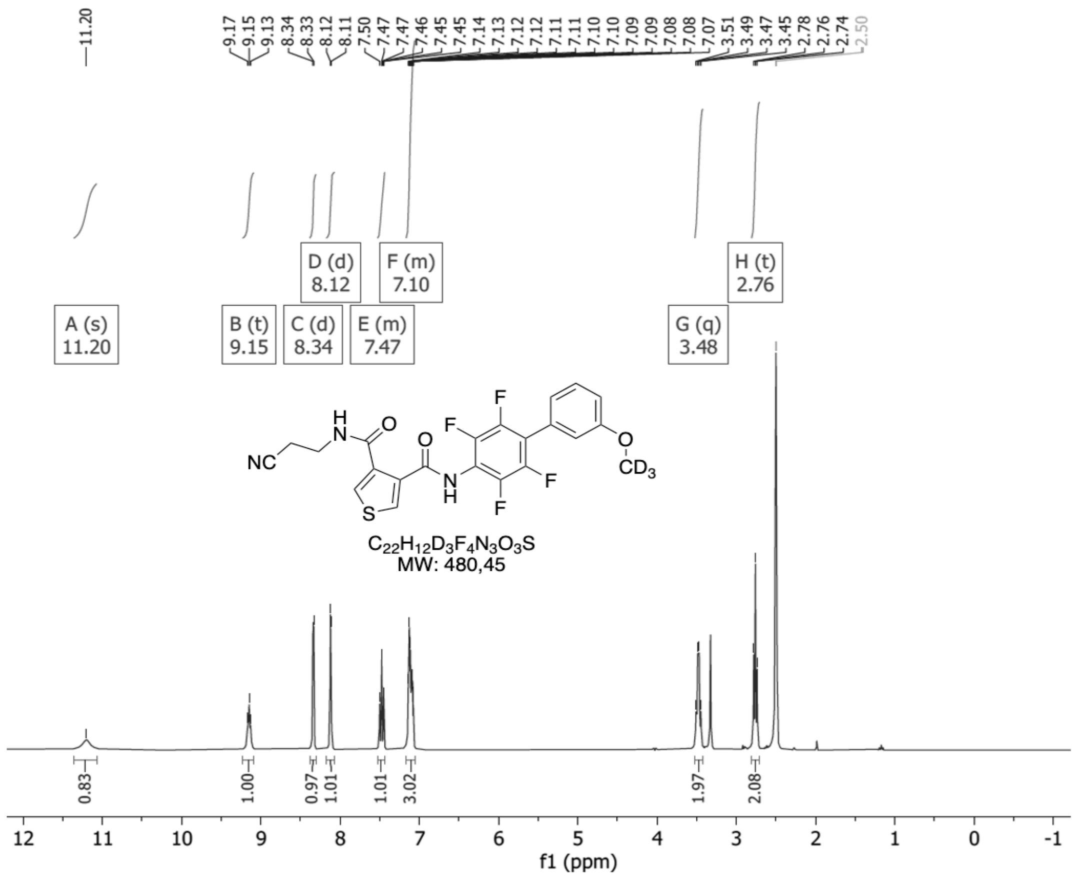

^{1}H-NMR (300 MHz, DMSO-d_6) of compound **42**

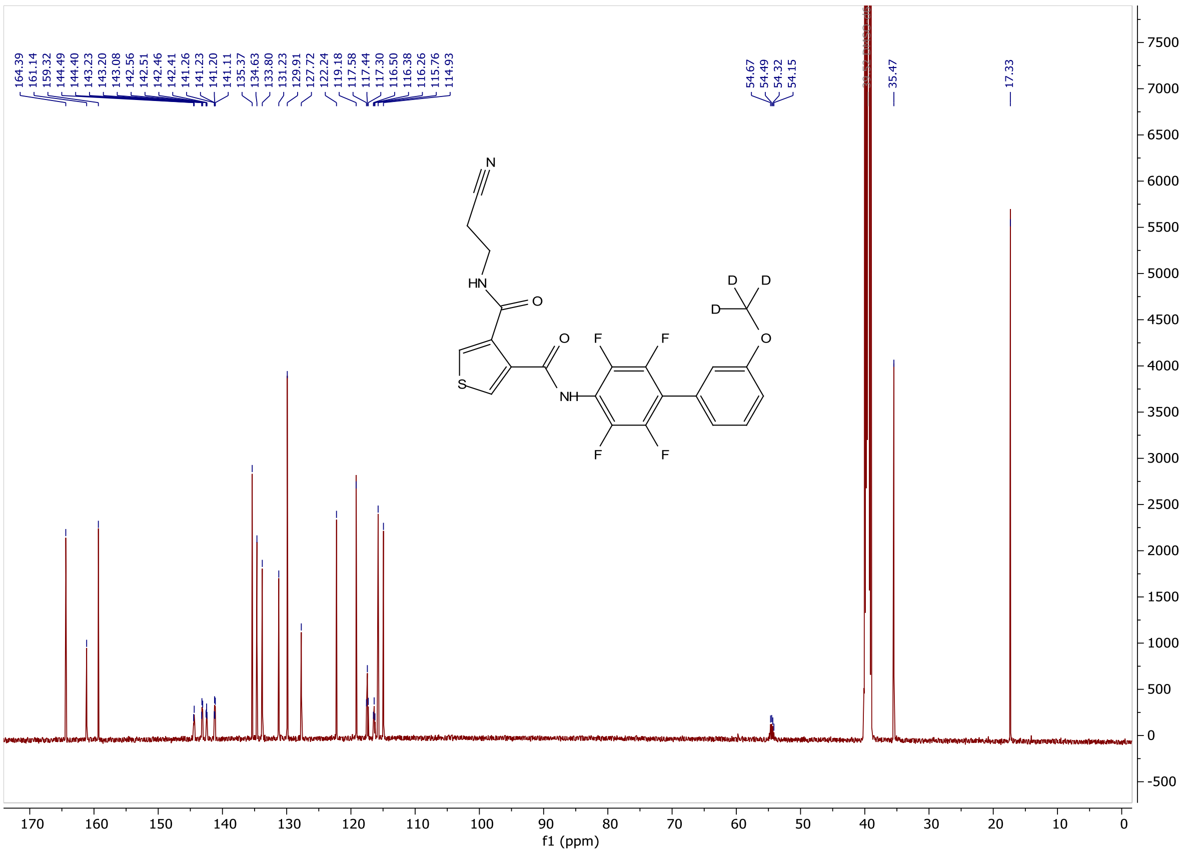

^{13}C-NMR (126 MHz, DMSO-d_6) of compound **42**

Peak ID	Compound	Time	Mass Found
3	Found	4.16	503.10,481.10

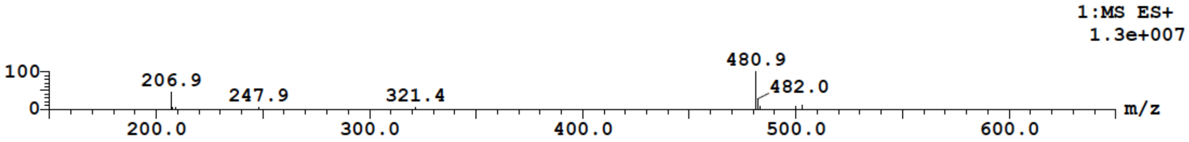

MS of compound **42**

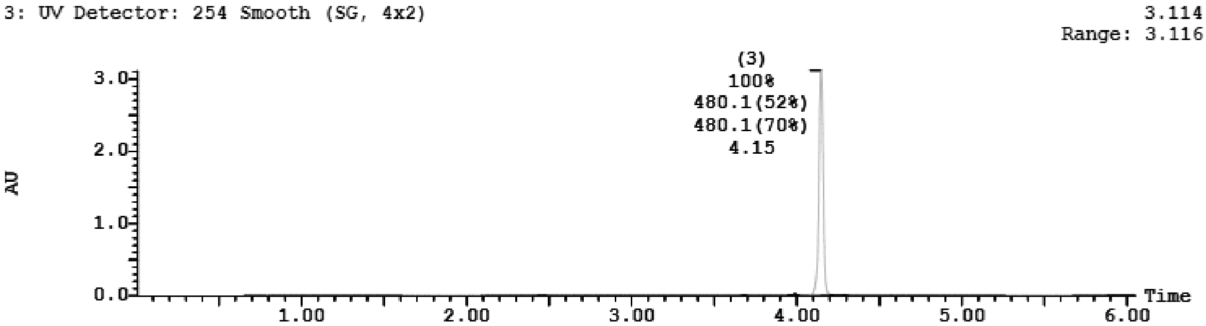

Peak Number	Compound	Time	AreaAbs	Area %Total	Width	Height	Mass Found
3	Found	4.15	9e+004	100.00	0	3e+006	480.10, 480.10

Chromatographic purity analysis of compound **42**

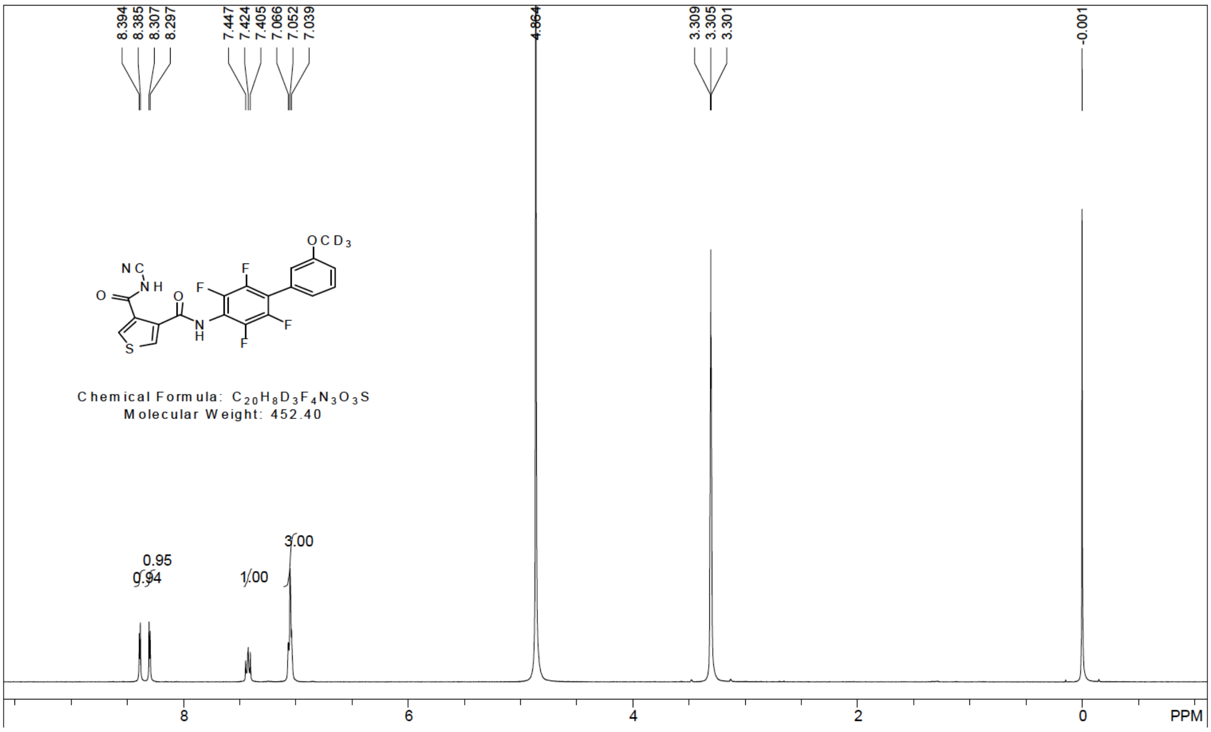

^{1}H-NMR (400 MHz, CD_3OD) of compound **43**

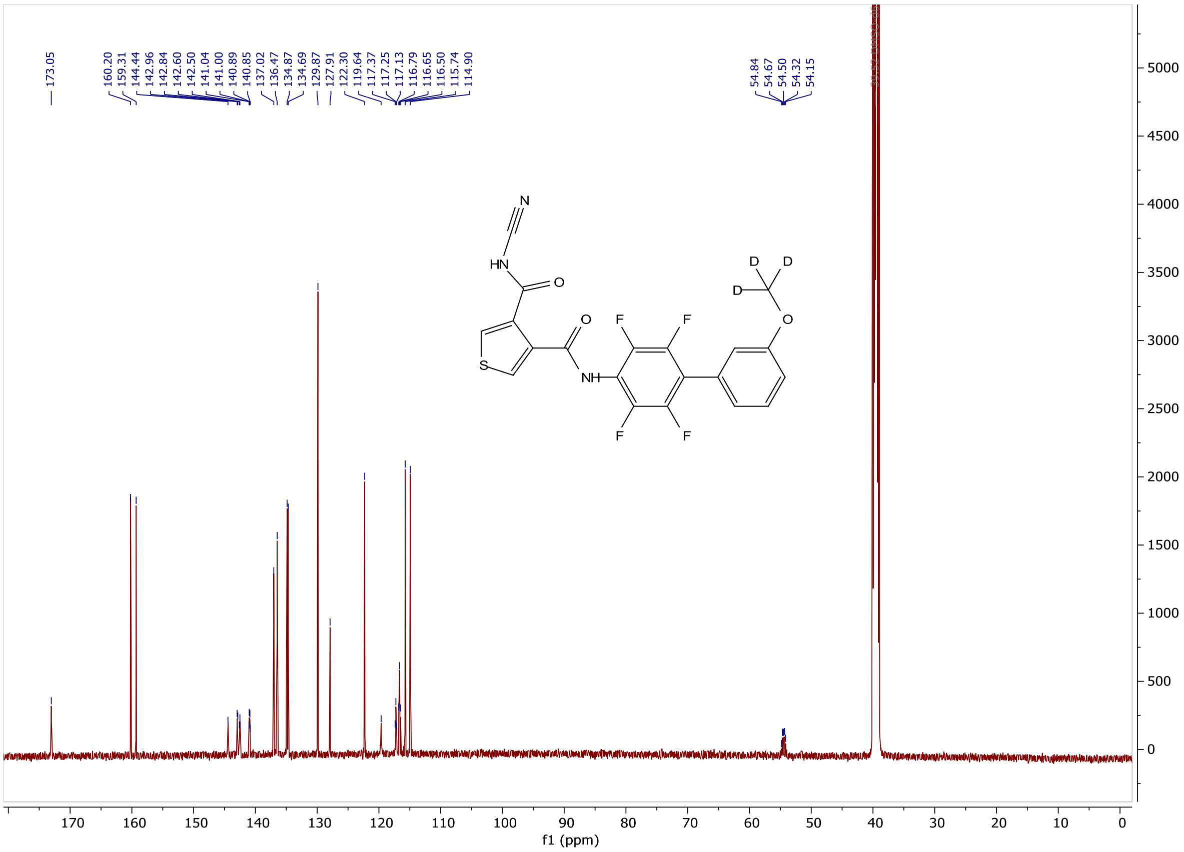

^{13}C-NMR (126 MHz, DMSO-d_6) of compound **43**

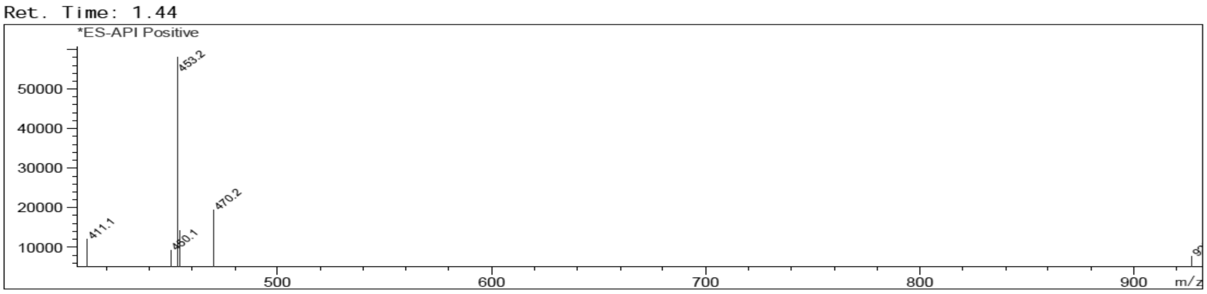

MS of compound **43**

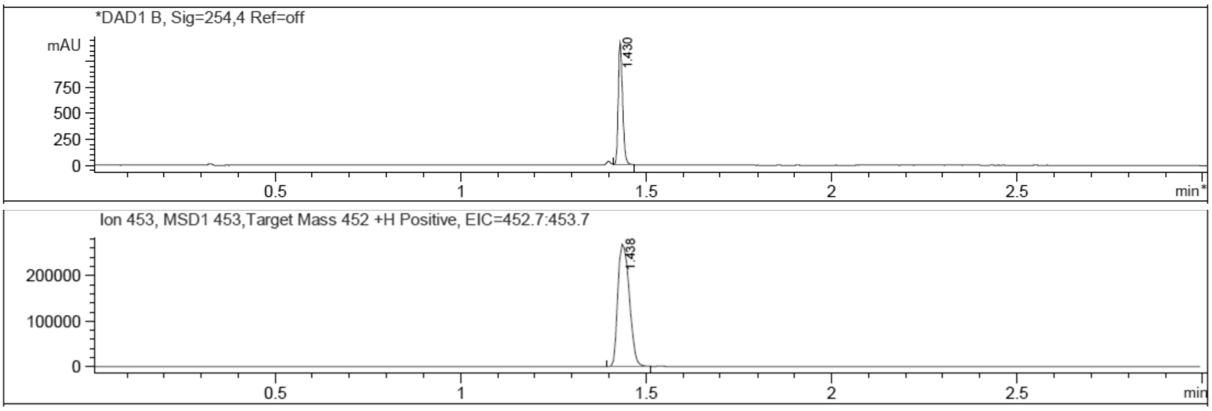

Chromatographic purity analysis of compound **43**

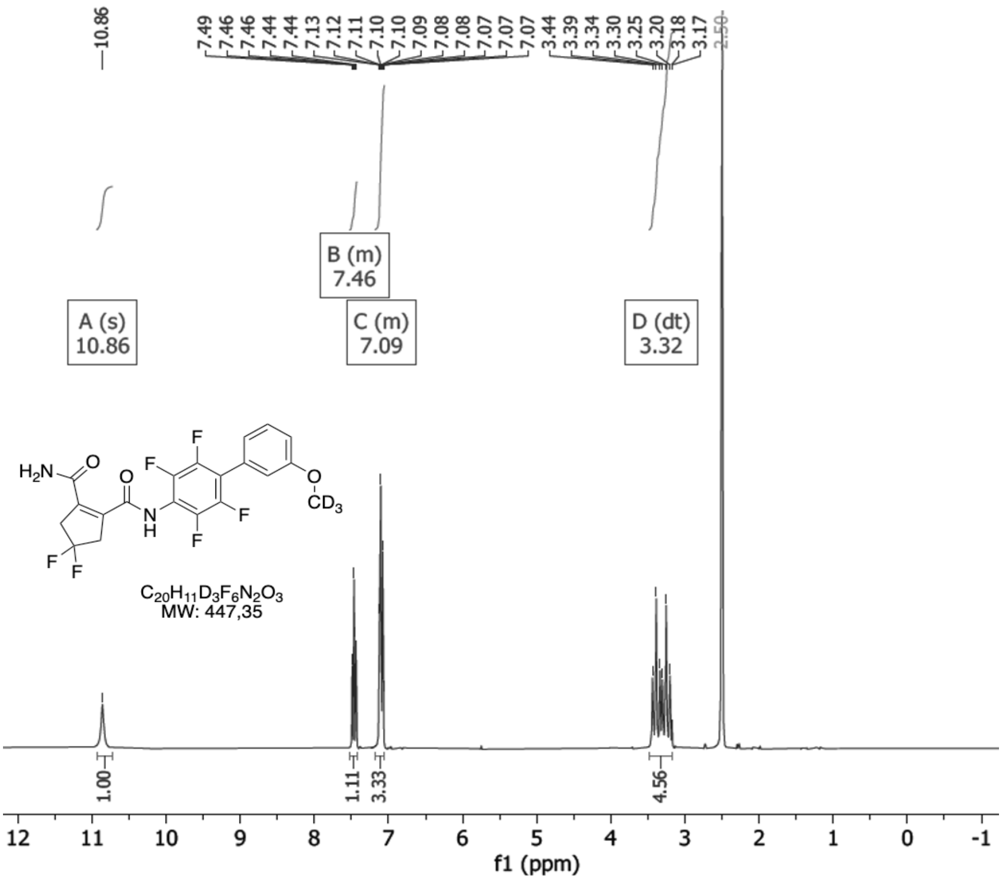

^{1}H-NMR (300 MHz, DMSO-d_6) of compound **45**

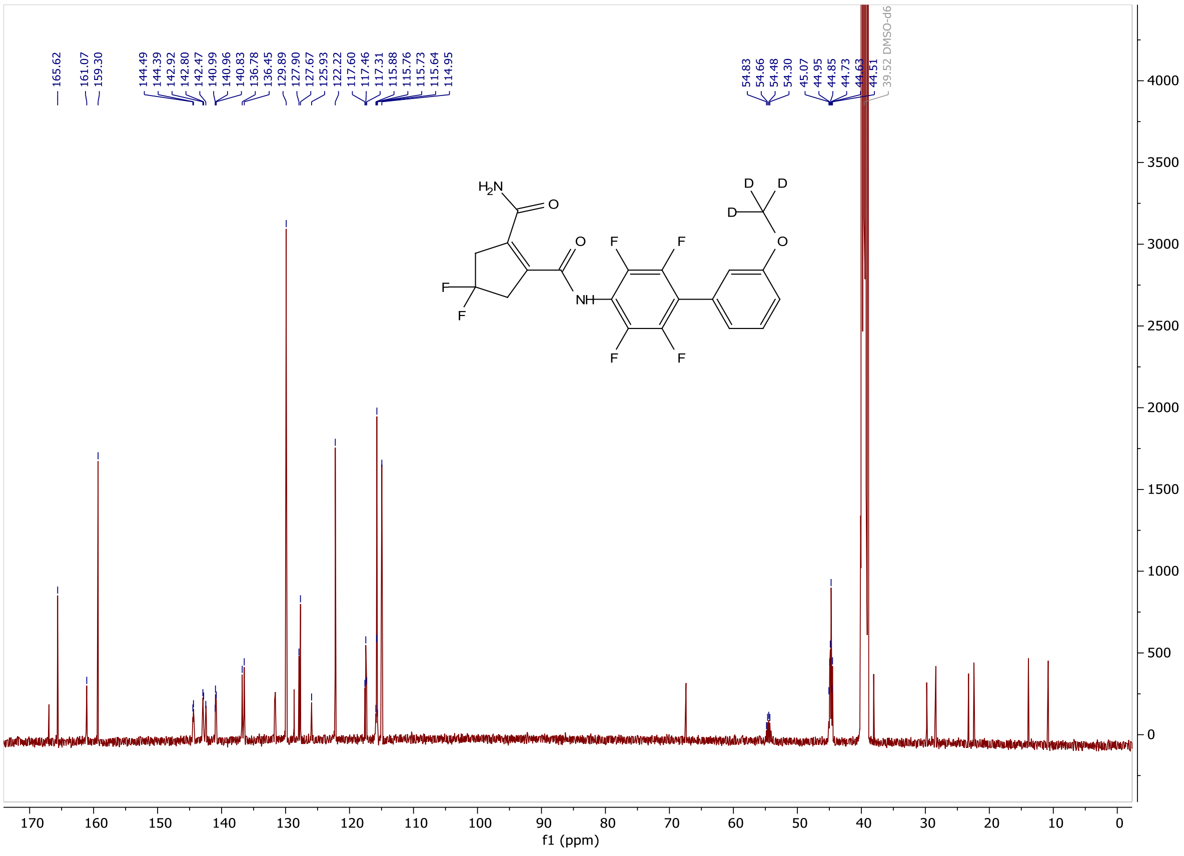

^{13}C-NMR (126 MHz, DMSO-d_6) of compound **45**

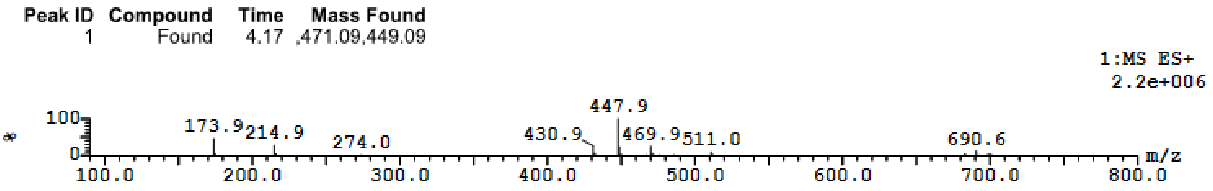

MS of compound **45**

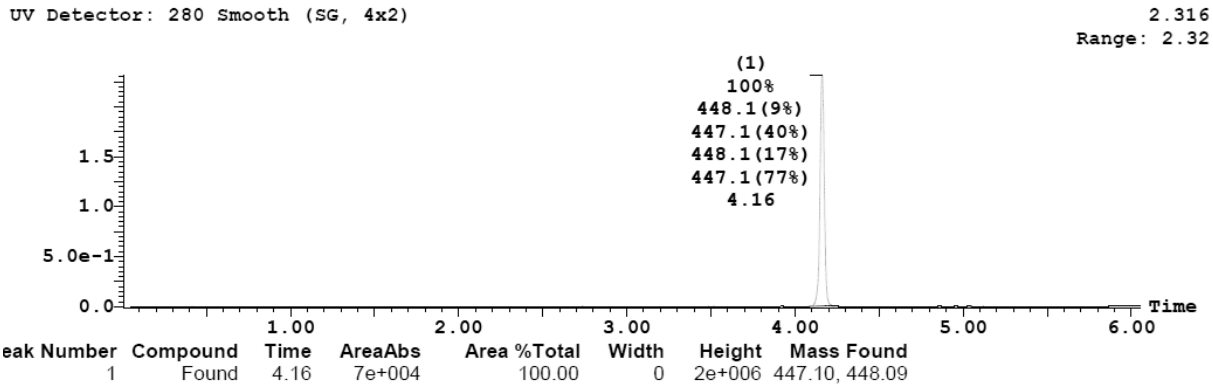

Chromatographic purity analysis of compound **45**

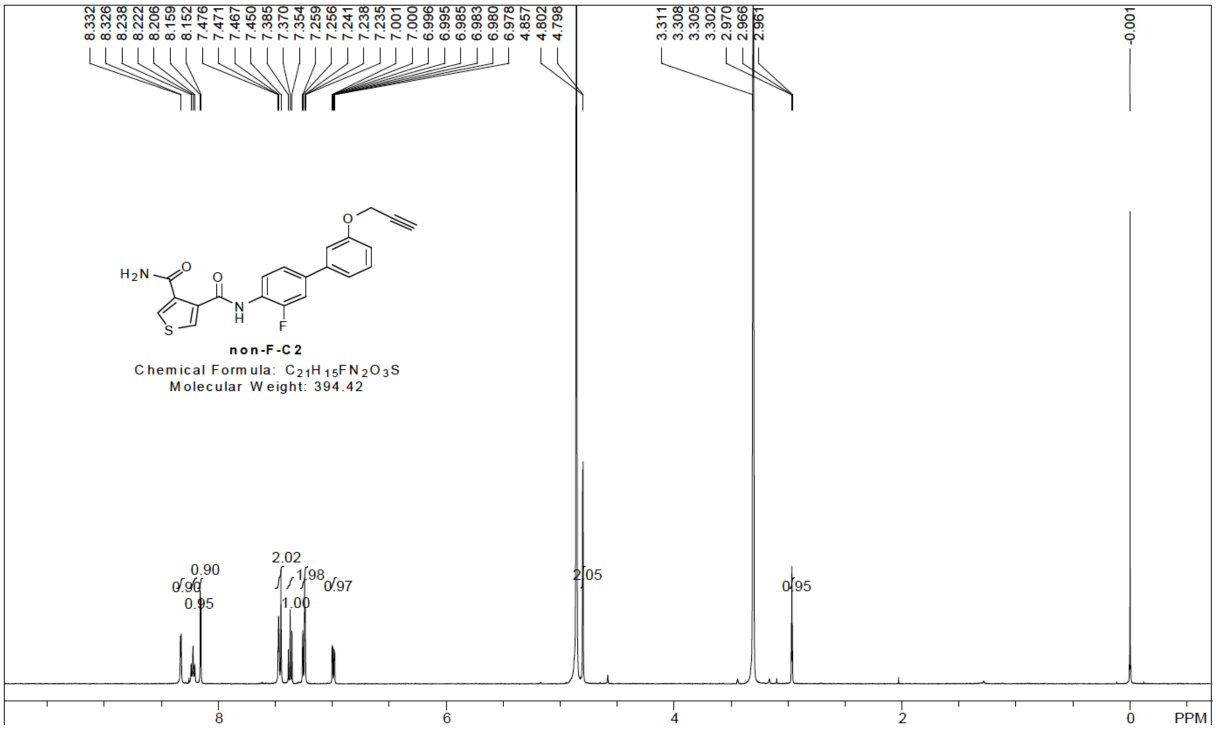

^{1}H-NMR (500 MHz, CD_3OD) of compound **47**

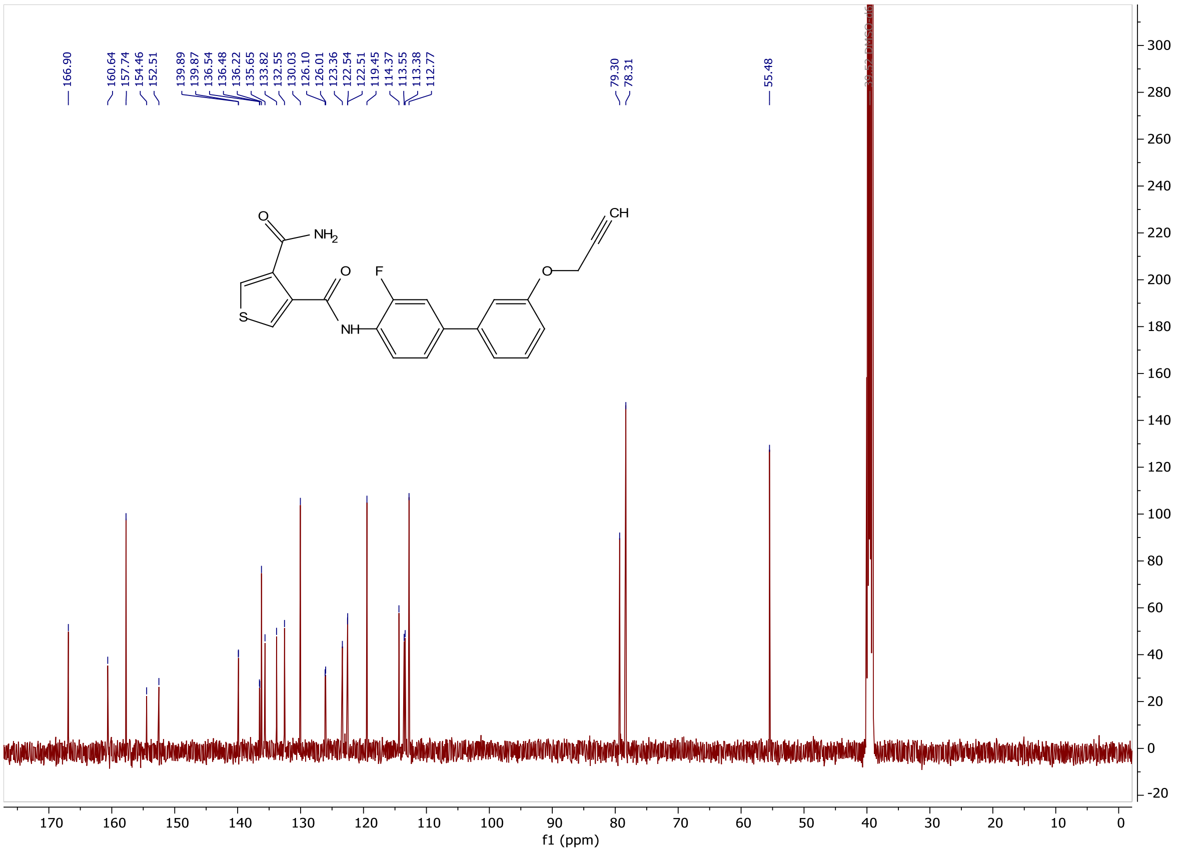

^{13}C-NMR (126 MHz, DMSO-d_6) of compound **47**

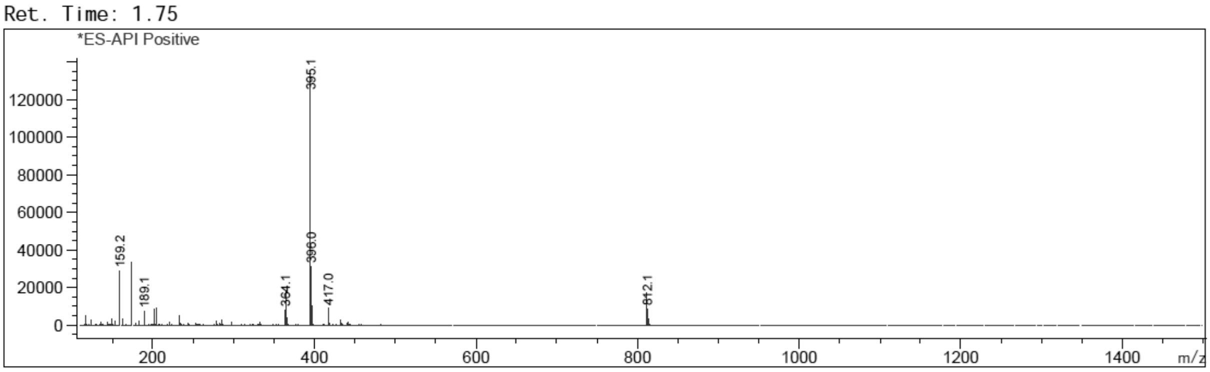

MS of compound **47**

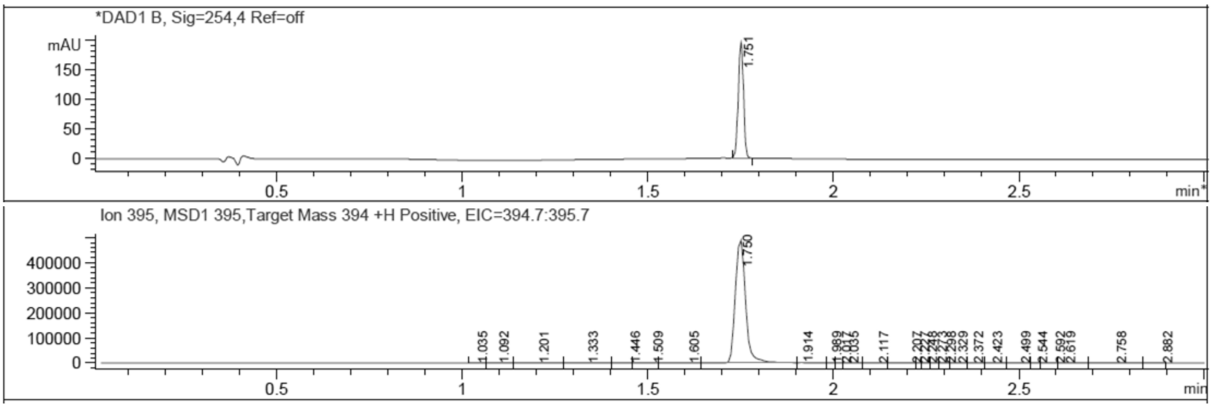

Chromatographic purity analysis of compound **47**
